# XXVII^e^ Actualités du Pharo. Alerte et réponse aux crises sanitaires: place des systèmes d'information 5-7 octobre 2022, Marseille, France

**DOI:** 10.48327/mtsi.v3i1.2023.332

**Published:** 2023-03-02

**Authors:** GISPE Organisateur :

**Affiliations:** 82 boulevard Tellène, 13008 Marseille, France, www.gispe.org

**Keywords:** Crises sanitaires, Épidémies, Pandémies, Zoonoses, Choléra, Peste, Ebola, Covid-19, Health crises, Epidemics, Pandemics, Zoonoses, Cholera, Plague, Ebola, Covid-19

Pour cette 27^e^ édition des Actualités du Pharo, le GISPE (Groupe d'intervention en santé publique et épidémiologie) a fait le choix de tirer quelques leçons des crises sanitaires infectieuses qui viennent de ravager notre monde, en particulier en milieu tropical. Parmi les nombreux angles d'approche possibles, notre Comité scientifique a décidé de s'attarder plus particulièrement sur les méthodes, les besoins et la place de l'alerte et de la réponse à ces crises sanitaires émergentes, réémergentes, épidémiques ou pandémiques, le plus souvent zoonotiques, qui bouleversent nos vies, nos sociétés, nos certitudes, notre orgueil d'hommes prétendument post-modernes, en portant un intérêt particulier aux besoins en systèmes d'information sanitaire toujours plus performants au Nord comme au Sud et à la manière de s'en servir. Au cours des sessions de conférences invitées et de communications orales et affichées, nous essaierons de faire le tour de l'existant, du nécessaire et des perspectives en cette matière depuis l'intérêt des données démographiques de routine, les apports d'une surveillance épidémiologique moderne, l'intérêt du concept de veille sanitaire, l'apport tant des techniques d'analyse biologique à haut débit que de la veille entomologique, la place de la modélisation pour l'anticipation puis la gestion de crise et bien sûr le financement de ces outils.

Cette année, une innovation supplémentaire vient compléter nos ambitions éditoriales avec la création d'une session proposée par la Société francophone de médecine tropicale et santé internationale (SFMTSI) qui a décidé de s'attacher à la problématique de la « permanence de l'accès aux soins en contexte épidémique » à travers les témoignages concrets d'experts ayant eu à défendre celle-ci dans le contexte du choléra, de la peste, d’Ébola et bien sûr de la Covid-19. Et ceci juste avant le toujours très attendu et instructif symposium du Collège des Universitaires de Maladies infectieuses et tropicales consacré aux actualités en médecine tropicale.

Mais pour patienter avant d'entrer dans le vif du sujet, n'hésitez pas à déguster le résumé de la conférence inaugurale proposée par le Professeur Anne-Marie Moulin qui croise philosophie et histoire pour comprendre ce « nouvel âge microbien» dans lequel nous sommes entrés.

Bienvenue à Marseille, mais aussi chez vous pour cette seconde édition mixte, en présence ou à distance, des Actualités du Pharo.

**Figure 1:**
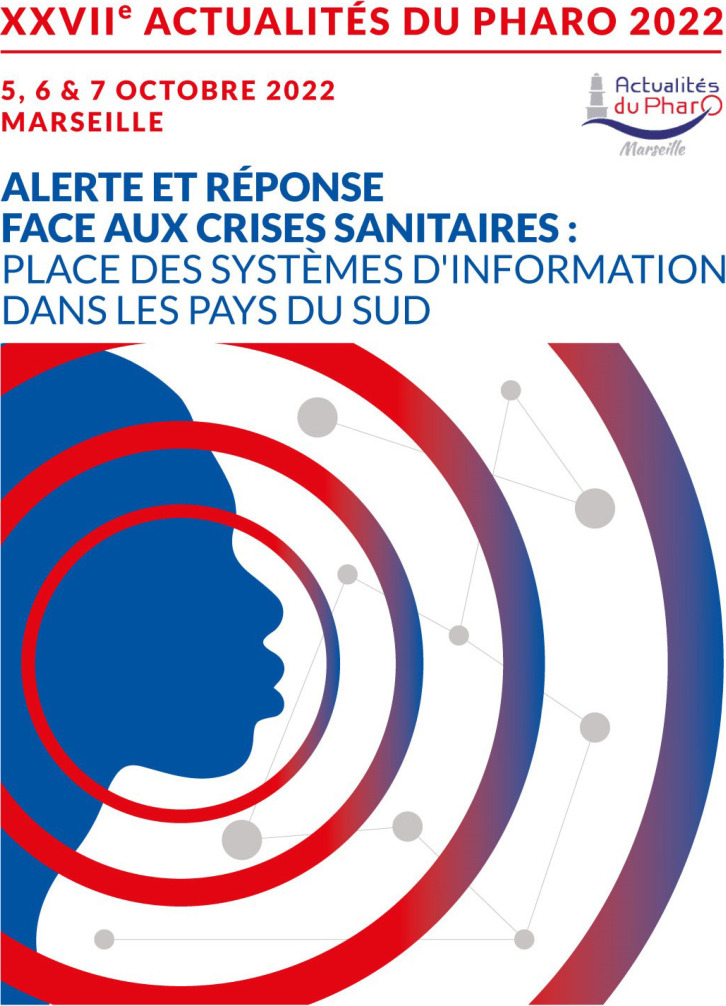
Affiche des XXVII^e^ Actualités du Pharo. Alerte et réponse aux crises sanitaires: place des systèmes d'information Poster of the XXVII^th^ Actualités du Pharo. Alert and response to health crises: the place of information systems

