# Supplementary material for: XXVIIth Actualités du Pharo. Alert and response to health crises: the place of information systems. 5-7 October 2022 Marseille, France
Source: Med Trop Sante Int. 2023 Mar 2;3(1):mtsi.v3i1.2023.332. [Article in French] doi: 10.48327/mtsi.v3i1.2023.332 (PMC10300677; doi:10.48327/mtsi.v3i1.2023.332)
Supplement: Related texts

# Research article: Croiser histoire et philosophie des épidémies: cycles ou émergences?

**Published:** 2023-03-02

**Authors:** Anne-Marie Moulin

**Affiliations:** CNRS, Sorbonne, Paris, France

**Keywords:** Épidémies, Histoire, Cycles, Émergences, Philosophie

L'histoire de l'humanité est jalonnée d’épidémies, contre lesquelles ont été inventées d'innombrables parades. Notre génération qui avait entamé un programme d’éradications se confronte à ce qu'elle considère non plus comme un cycle ou éternel retour des mêmes maux, mais comme une succession d’émergences, difficiles à prévenir et, le moment venu, à gérer. Comment comprendre ce paradoxe de la modernité? Où trouver l'explication et, plus important, comment collecter l'information pertinente et donner un sens à l'expression qui tend à être galvaudée, de santé globale, quand le droit universel à la santé s'avère largement une fiction? Dans le sillage de la démarche de l'anthropologue et médecin Paul Farmer disparu en avril 2021, infatigable dénonciateur des inégalités de santé, Anne-Marie Moulin se propose de croiser philosophie et histoire pour éclairer un avenir que certains décrivent comme un « nouvel âge microbien ».

# Research article: La veille sanitaire à l’épreuve de la pandémie de Covid-19

**Published:** 2023-03-02

**Authors:** Antoine Flahault

**Affiliations:** Institut de santé globale, Université de Genève, Genève, Suisse

**Keywords:** Veille sanitaire, Surveillance épidémiologique, Biobanques, Covid-19, Pandémie

La veille sanitaire vise à alerter et informer les politiques publiques en suivant en permanence la situation épidémiologique et son évolution sur un territoire. Connectée aux réseaux internationaux de sécurité sanitaire et coordonnée par les agences gouvernementales et intergouvernementales (ECDC, OMS), la veille sanitaire d'un territoire s'inscrit dans le contexte épidémiologique international qu'elle enrichit de ses données et de ses analyses. Cette conférence dressera un premier bilan en comparant trois modèles de veille sanitaire déployés en Europe pour la surveillance de la pandémie de Covid-19: ceux de la France, du Danemark et du Royaume-Uni. Le degré d'intégration des données de santé, la qualité méthodologique des estimations fournies, la pertinence des analyses seront discutées dans les trois pays au regard des mesures prises par leurs décideurs politiques. Leur performance, leurs insuffisances et leur contribution à l’éclairage sur la riposte contre la pandémie seront alors analysées. La conclusion de la conférence visera à évaluer l'opportunité de réfléchir à une refonte des instruments de veille sanitaire afin de fournir une information de meilleure qualité aux médias, au public et aux autorités chargées de la santé de la population.

# Research article: Surveillance pour l'alerte et la réponse aux crises sanitaires (notamment en milieu tropical)

**Published:** 2023-03-02

**Authors:** Arnaud Tarantola

**Affiliations:** Centre d'intervention en région (CIRE) Île de France, Santé publique France, Paris, France

**Keywords:** Surveillance, Alerte, Syndromique, Émergence

Les systèmes de surveillance, leur qualité et leur pertinence conditionnent la capacité à détecter et donner l'alerte ainsi que la réponse à l'alerte en santé publique. La surveillance épidémiologique vient aussi étayer la réponse en santé publique, tant dans la programmation que le suivi des programmes de prévention. Les maladies émergentes constituent l’épreuve ultime des systèmes de surveillance, qui doivent être capables de détecter sinon le premier cas tout du moins les premiers signes d'une épidémie naissante et d'en caractériser de manière rapide mais fiable les paramètres épidémiologiques. Les pays en développement présentent des défis mais disposent aussi de solides atouts en matière de surveillance et de détection des émergences, notamment des anthropo-zoonoses. Nous discuterons les finalités de la surveillance, les types de systèmes que l'on peut mettre en œuvre dans divers milieux et quelques leçons (durement) apprises.

# Research article: Alerte et pandémie: utilité des métadonnées de mobilité des opérateurs de téléphonie mobile

**Published:** 2023-03-02

**Authors:** Ousmane Ly

**Affiliations:** Consultant international en santé publique, Sherbrooke, Canada

**Keywords:** Alerte, Épidémie, Pandémie, Métadonnées, TIC

L'alerte se définit comme l'avertissement visant à prévenir un danger ou un évènement ayant un fort potentiel de risque. Les épidémies sont considérées comme de tels évènements, surtout si elles conduisent à une pandémie, ce qui a été le cas des dernières situations sanitaires mondiales. En effet, les virus du SARS-CoV-2 et de la variole du singe ont conduit à des pandémies, conformément aux règlements sanitaires internationaux établis par l'Organisation mondiale de la Santé (OMS). Le contrôle des épidémies pour qu'elles ne conduisent pas à des pandémies passe nécessairement par la mise en place de systèmes d'alerte précoce (SAP). Ces systèmes sont au cœur des mesures permettant de limiter la perte de vies et de moyens de subsistance due aux aléas et aux catastrophes. Ils sont constitués d'une série de mécanismes de surveillance organisée ou d'actions qui collectent des informations sur les aléas potentiels dans un lieu donné, afin de déclencher en temps opportun des interventions coordonnées. Les technologies de l'information et de la communication (TIC) offrent des opportunités certaines pour améliorer les systèmes d'alertes. Cela s'est confirmé par l'utilisation des métadonnées de mobilité des opérateurs de téléphonie mobile à Kinshasa durant la Covid-19. Ces données anonymes et agrégées des opérateurs de réseaux mobiles (ORM) constituent une source clé pour comprendre la mobilité de la population. Elles fournissent des informations qui peuvent améliorer la prise de décision et la planification de scénarios pendant l’épidémie de Covid-19. Ces données ont été analysées en temps quasi réel pour fournir un aperçu des schémas de mobilité à travers la République démocratique du Congo (RDC). Cela dans le but de mettre à la disposition des décideurs des informations sur la mobilité des populations, permettant ainsi de prédire précocement les zones d’éclosions de la maladie.

Aujourd'hui l'avertissement ou l'alerte bénéficie de l’évolution technologique au profit des systèmes de santé pour mieux outiller la lutte contre les épidémies, et surtout la prévention précoce des pandémies. Cela passe nécessairement par la mise en place de systèmes d'alerte précoce sanitaire (SAPS) utilisant le potentiel immense des réseaux sociaux, l'opportunité de disponibilité des métadonnées de mobilité, la puissance de calcul des systèmes informatiques, la précision de la géolocalisation, le traitement automatisé des données avec les technologies d'intelligence artificielle, etc. Il en résulte un meilleur contrôle des épidémies afin d’éviter la survenue récurrente de pandémies.

# Research article: Ébola à l'horizon 2030, chronique d'une maladie épidémique menaçante à l’épreuve de l'innovation centrée sur le patient

**Published:** 2023-03-02

**Authors:** Denis Malvy

**Affiliations:** Unité de médecine tropicale et santé internationale, CHU de Bordeaux, INSERM 1219, Bordeaux, France

**Keywords:** Ébola, Épidémie, Réponse, Innovation, Crises

Le virus Ébola est l'agent causal de deux récentes urgences de santé publique de portée internationale, l’épidémie de 2018-2020 dans les provinces du nord-est de la République démocratique du Congo (RDC) et l’épidémie de 2013-2016 qui a sévi en Afrique de l'Ouest et a été responsable de 28 616 cas notifiés et 11 310 décès. Ce virus a été identifié pour la première fois en 1976 en RDC, et cette contrée d'Afrique centrale et de vaste étendue a désormais connu quatorze épidémies. Les épidémies qui ont précédé celle d'Afrique de l'Ouest (avec ses expansions urbaines) avaient émergé dans des environnements reculés. Elles étaient associées à une production moyenne de moins de 300 cas notifiés, avec un taux de létalité très élevé dépassant 70% et une extinction rapide accompagnée des mesures de riposte restreintes à des procédures d'isolement.

Les épidémies massives de 2013-2016 et 2018-2020 ont produit plusieurs milliers de personnes considérées comme guéries. Ces personnes dénommées « vainqueurs » d’Ébola sont susceptibles de connaître des manifestations séquellaires post-infectieuses ou le fardeau d'une stigmatisation sociale comme double peine du survivant. À titre emblématique, l’épidémie de 2018-2020 dans la région des Grands Lacs s'est inscrite dans un contexte critique de niveau de soin sous-dense, d'instabilité sociale et politique majeure, de réticence et déni de la part des populations affectées, et de guerre civile. Tous ces éléments concourent à la défiance et même à la violence vis-à-vis des équipes de professionnels de santé engagées dans la riposte. Cette situation sanitaire extrême aura néanmoins connu d'une part, le déploiement d'un vaccin expérimental dans l'objectif d'interrompre les chaînes de transmission chez les contacts de patients excréteurs et d'autre part, la mise en œuvre d'un essai clinique pour évaluer l'efficacité de traitements spécifiques prometteurs.

Le cadre emblématique de la réponse à Ébola est figuré par l'infrastructure de prise en charge individuelle (traitement et isolement) du patient mise en œuvre par les équipes de médecine humanitaire et dénommée centre de traitement Ébola (CTE). En 2019 et malgré les infortunes infligées de la part de groupes factieux, le CTE a connu une évolution majeure en termes d'innovation architecturale et de positionnement résolu au plus près des communautés exposées. Ces mesures d'innovation ont permis non seulement une densification du soin de support, mais aussi l'accès à des membres de la famille comme visiteurs auprès du patient, sans déroger aux principes de sécurité vis-à-vis du risque viral. Le CTE cesse d’être un lieu exclusif de quarantaine, mais devient un espace capable de restauration de confiance intégrant la contrainte de l'urgence, et dans lequel se construit un projet de soin visible où i) le meilleur niveau de prise en charge est proposé, ii) l'information disponible pour le patient hospitalisé et son entourage est donnée, iii) et enfin le meilleur niveau d'information est produit par la recherche clinique au bénéfice des patients à venir. Cet espace de conciliation entre bénéfice individuel et obligation de santé publique développé en contexte d'urgence humanitaire majeure et de guerre rejoint de manière singulière le concept de la médecine centrée sur le patient et l'accès à l'information soutenant la décision médicale, développé au niveau international en situation d'accès au droit à la santé. À l'horizon 2030, l'expérience d’Ébola en matière d'innovation dans le projet et l'organisation du soin est à porter à la gestion des futures expressions de maladies menaçantes à propension épidémique au Nord comme au Sud.

# Research article: Financement des institutions de veille sanitaire par l'AFD: vers une approche intégrée

**Published:** 2023-03-02

**Authors:** Pierre Crozier

**Affiliations:** Agence française de développement (AFD), Paris, France

**Keywords:** AFD, Information sanitaire, Surveillance épidémiologique, One Health

Le Groupe AFD poursuit ses actions en faveur de l'amélioration de la sécurité sanitaire aux niveaux national et régional, selon une approche « One Health », pour laquelle son expertise, ses outils techniques et financiers et ses modalités d'intervention avec des partenaires de confiance lui offrent une réelle plus-value. Ainsi, l'AFD finance de nombreux projets qui ciblent le soutien à la surveillance épidémiologique intégrée (humaine, animale, y compris entomologique, et environnementale), en lien avec le renforcement des laboratoires et de réseaux intégrés au niveau national et régional, ainsi que le soutien à l'innovation et aux transferts technologiques (Sud-Sud et Nord-Sud).

# Research article: Émergence et diffusion de la résistance aux antibiotiques: problématique dans les pays du Sud

**Published:** 2023-03-02

**Authors:** Abdoul Salam Ouedraogo

**Affiliations:** Laboratoire de bactériologie et de virologie, CHU Souro Sanou, Bobo-Dioulasso, Burkina Faso

**Keywords:** Résistance, Antibiotiques, Pays à ressources limitées

L’émergence et la diffusion de la résistance aux antibiotiques représentent une menace majeure de santé publique. Les données récentes de la littérature abondent de descriptions de bactéries multirésistantes voire toto-résistantes aux antibiotiques dont le nombre ne cesse de croître, aussi bien dans les pays industrialisés que dans les pays en développement.

À l’échelle mondiale, la principale cause de cette émergence de la résistance est une consommation non raisonnée des antibiotiques. Dans les pays à ressources limitées, d'autres facteurs plus spécifiques, socio-économiques et comportementaux, contribuent à exacerber cette émergence de la résistance aux antibiotiques. Parmi eux, on peut citer: i) certaines pratiques sociétales fréquentes comme l'automédication; ii) une filière médicale défaillante avec des prescripteurs insuffisamment formés et des outils diagnostiques peu performants; iii) ou encore, une filière du médicament non contrôlée avec des antibiotiques en vente libre, stockés inadéquatement, contrefaits ou/et périmés.

La situation est pourtant alarmante dans ces pays à ressources limitées où les conditions d'hygiène déplorables facilitent la transmission des infections bactériennes tant au niveau interhumain qu'animal et environnemental.

Dans ces pays, les maladies infectieuses, la pauvreté et la malnutrition sont endémiques. Les diarrhées, les infections respiratoires, les méningites bactériennes et les autres maladies infectieuses ont en effet augmenté la consommation non rationnelle d'antibiotiques tant dans le cadre de traitements symptomatiques que de la prophylaxie. En outre, l'utilisation des antibiotiques de dernière génération dans la filière animale contribue à aggraver la situation.

Dans ce contexte d’émergence de la résistance aux antibiotiques, des interventions urgentes sont nécessaires parmi lesquelles: i) la prévention des infections par la promotion de l'hygiène individuelle, collective et environnementale; ii) la sécurisation de la filière des médicaments en général et des antibiotiques en particulier en entreprenant des réformes drastiques visant à contrôler rigoureusement la mise sur le marché des antibiotiques, la règlementation de leur dispensation aux populations, voire à la sanctuarisation des antibiotiques à large spectre aux structures hospitalières; iii) la prévention de la transmission de la résistance qui permettra, grâce au dépistage des patients et du personnel colonisés ou infectés, de réduire la transmission des micro-organismes résistants d'une personne à une autre mais aussi la diffusion à plus grande échelle à travers les établissements de soins; iv) la surveillance de la résistance au niveau biologique, essentielle pour connaître l'ampleur du problème et faciliter la mise à jour les listes de médicaments essentiels et les directives nationales de traitement, ainsi qu’à évaluer les effets des différentes stratégies de traitement mises en place. Pour être efficaces, toutes ces stratégies d'intervention doivent être intégrées et cibler simultanément les décideurs, les prescripteurs et les utilisateurs.

# Research article: Capacité des programmes de lutte antivectorielle dans l'alerte et la détection précoces des épidémies de maladies vectorielles en Afrique de l'Ouest

**Published:** 2023-03-02

**Authors:** Abdoul-Aziz Maiga

**Affiliations:** Entomologie médicale, Université Joseph Ki-Zerbo, Ouagadougou, Burkina Faso

**Keywords:** Développement de capacités, Alerte et détection précoces, Maladies vectorielles, Programmes de lutte antivectorielle, Afrique de l'Ouest

En Afrique de l'Ouest, les facteurs de risques sociaux, environnementaux, urbains et globaux de l’émergence et de la réémergence des maladies vectorielles s'accroissent, favorisant ainsi l'exposition des populations aux piqûres des vecteurs et donc à la transmission des pathogènes.

L'alerte et la détection précoces des épidémies permettent de limiter la propagation des pathogènes, et donc de sauver des vies humaines et du bétail ainsi que de préserver l’économie des populations.

Cette communication révèle les forces et faiblesses des programmes nationaux de lutte antivectorielle en Afrique de l'Ouest, et les goulots d’étranglements affectant la bonne implémentation des activités de surveillance des vecteurs qui représente le principal pilier devant permettre: i) l'adoption des mesures appropriées de prévention, ii) l'alerte et la détection précoces des épidémies et iii) la réponse (contrôle) appropriée (basée sur l’évidence) en un temps raisonnable à une éventuelle épidémie, afin d'en réduire l'incidence.

# Research article: Place des outils d'analyse biologique à haut débit (« omics ») dans l'alerte et la surveillance

**Published:** 2023-03-02

**Authors:** Pierre-Édouard Fournier

**Affiliations:** Institut hospitalo-universitaire Méditerranée Infection, UMR Vitrome, Marseille, France

**Keywords:** Génomique, Métagénomique, Transcriptomique, Alerte, Surveillance

La pandémie de Covid-19 a mis en évidence les risques liés aux réservoirs zoonotiques d'agents pathogènes pour l'homme ainsi que la facilité de propagation des maladies infectieuses liée à la mondialisation croissante des échanges de population. Dans ce contexte, les outils d'analyse biologique à haut débit (« omics ») ont démontré tout leur potentiel pour permettre de comprendre l’épidémie. La génomique, par le séquençage massif des génomes des souches de SARS-CoV-2, a permis de comprendre les mécanismes de diffusion du virus et d'appréhender son évolution. À ce jour, plus de 11 millions de séquences virales ont été produites dans le monde. En parallèle, les outils de séquençage à haut débit ont été adaptés à la caractérisation des flores microbiennes complexes par l'identification de tout acide nucléique dans un échantillon donné (métagénomique). Initialement réservée à l’étude du microbiote, la métagénomique a été rapidement adaptée au diagnostic rapide, permettant sans a priori de détecter dans un échantillon clinique tout type de micro-organisme. Enfin, la métatranscriptomique, dont l'objet est d'identifier les gènes transcrits par un organisme donné, permet à présent de détecter des profils spécifiques d'infection bactérienne, virale ou parasitaire, allant jusqu’à caractériser le type d'agent en cause. Outre leur performance technologique et la quantité de séquences produites, les techniques de séquençage à haut débit peuvent être mises en œuvre très rapidement, une réaction de séquence pouvant être produite à partir de tout type d’échantillon, dans tout type d'environnement, et en moins de 10 heures. Ces caractéristiques font des « omics » des outils particulièrement adaptés à la surveillance et à l'alerte des risques infectieux.

# Research article: Modélisation pour l'anticipation et la gestion de crise épidémique

**Published:** 2023-03-02

**Authors:** Gaëtan Texier

**Affiliations:** Centre d’épidémiologie et santé publique des armées, UMR Vitrome, Marseille, France

**Keywords:** Épidémie, Anticipation, Modélisation, Crises

Présentation des apports des modèles (compartimentaux,multi-agents,statistiques et IA…) dans le cadre de l'anticipation et la gestion de crise épidémique.

# Research article: L'information sanitaire issue du système de soins: passer d'une logique gestionnaire à une démarche de santé publique

**Published:** 2023-03-02

**Authors:** Gérard Salem, Florence Fournet

**Affiliations:** Université Paris-Nanterre, France; Entomologie médicale, Institut de recherche pour le développement (IRD), France

**Keywords:** Système d'information, Système de soins, Offre, Besoins

Chacun sait que l'activité enregistrée dans les structures de soins ne renseigne que peu sur l’état de santé de la population, sur la fraction de la population y ayant eu accès, celle qui a été enregistrée, selon des codes de qualité variable et souvent peu comparables. Les données collectées servent donc essentiellement à la gestion des structures de soins, dans une logique de l'offre et de la demande. La diversification de l'offre de soins dans les villes africaines – offre privée, caritative, voire association d'une offre « moderne » et « traditionnelle », rend l'entreprise encore plus délicate.

De nombreux exemples montrent pourtant qu'on pourrait mieux utiliser ces données en cherchant à évaluer la véritable accessibilité aux soins, et la satisfaction des besoins.

# Research article: Mesurer les niveaux et tendances de la mortalité en population générale: comment pallier l'absence d'un état civil fonctionnel?

**Published:** 2023-03-02

**Authors:** Géraldine Duthe

**Affiliations:** Institut national d’études démographiques (INED), France

**Keywords:** Démographie, Sources, Enquête, État civil, Estimation

Si d'importants progrès en matière de santé ont été menés depuis les années 1950 dans les pays du Sud, il n'en a pas été de même pour ce qui est de l'enregistrement systématique des décès et de leurs causes dans les pays à revenu faible et intermédiaire. En dehors de l'Amérique latine, les statistiques d’état civil sont toujours très incomplètes voire inexistantes, et d'importants enjeux portent sur la mesure des indicateurs de santé disponibles. Nous présenterons les principales sources de données alternatives qui permettent d'estimer la mortalité en population générale (recensements, enquêtes basées sur des échantillons, suivis démographiques au niveau local), les estimations associées, leurs intérêts et leurs limites. Aujourd'hui, si la mortalité des enfants est relativement bien estimée, il n'en est pas de même pour celle des adultes et pour la mortalité par cause. Or avec la transition sanitaire et le vieillissement de la population, les besoins de connaissance ont changé. Cette situation, pourtant dénoncée de longue date, ne permet pas d’étudier de manière détaillée les problèmes de santé adulte, ni d’évaluer rapidement l'impact d'une crise sanitaire telle que la pandémie de Covid-19. Nous terminerons cet exposé sur les initiatives actuelles pour remédier à cette situation et les défis qu'elles posent en matière d’éthique notamment.

# Research article: La surveillance des zoonoses au cœur de la prévention des crises sanitaires

**Published:** 2023-03-02

**Authors:** Marisa Peyre

**Affiliations:** Unité de recherche ASTRE, Centre de recherche agronomique pour le développement international (CIRAD), France

**Keywords:** Surveillance, Émergence, Prévention, Communautés, One Health

La crise Covid-19 en cours a mis en évidence l'importance de prévenir les risques d’émergence de maladies plutôt que d'avoir à assumer leurs conséquences sanitaires, sociales et économiques. La mise en place de stratégies de prévention, impliquant la détection précoce des évènements d’émergence par le biais de systèmes de surveillance efficaces au niveau local coûterait 100 fois moins cher que les coûts engendrés par une pandémie. Plutôt que de réagir aux émergences de maladies zoonotiques, il faut donc les éviter. Pour cela, il faut réduire les risques et co-construire des solutions de prévention avec les acteurs en première ligne face à ces risques (communautés locales, éleveurs, chasseurs, agents de l'environnement, de la santé etc.). Il faut réagir vite: des actions rapides doivent être menées à la source pour éviter la propagation de la maladie aux premiers signes d’émergence. Pour réagir rapidement, il faut avoir des données fiables et des systèmes de surveillance efficaces du local au global. Pour que les actions soient menées, il faut qu'elles soient comprises et acceptées, ce qui implique l'engagement de tous les acteurs de la surveillance mais aussi la sensibilisation de la société civile dans un dialogue science-société-politique continu. Des solutions et des exemples pour mettre cela en pratique, notamment dans le cadre de l'initiative internationale PREZODE, seront présentés lors de cette conférence.

# Research article: Le suivi des évolutions sanitaires en temps réel sur la base des registres de décès à Antananarivo

**Published:** 2023-03-02

**Authors:** Bruno Masquelier

**Affiliations:** Démographe, Université de Louvain, Louvain, Belgique

**Keywords:** Registres de décès, Causes de décès, Madagascar, Covid-19

Dans la plupart des pays africains, les tendances de la mortalité par cause sont estimées à partir de modèles épidémiologiques car la couverture du système d'enregistrement des faits d’état civil reste faible et les statistiques disponibles sur les causes de décès issues des structures de soins ne sont pas représentatives au niveau national. Dans ce contexte, le système de notification des décès mis en place à Antananarivo est relativement unique car il permet de suivre avec précision l’évolution de la mortalité et la charge des principales maladies. Dans cette communication, nous évaluerons d'abord la performance de ce système de notification à travers six dimensions: l'exhaustivité de l'enregistrement des décès, la qualité de la déclaration des causes de décès, la qualité de la déclaration de l’âge et du sexe, la cohérence interne des données, le niveau de détail des causes de décès, ainsi que leur actualité. Deux illustrations seront ensuite fournies pour démontrer l'intérêt de tels systèmes de notification des décès: l'analyse de la saisonnalité des décès par cause, et le suivi quasiment en temps réel des perturbations introduites par la pandémie de Covid-19. Le cas relativement unique d'Antananarivo sera discuté au regard de la situation dans les autres centres urbains de Madagascar et dans les autres pays africains où les lacunes en matière de données sur les causes de décès restent colossales.

# Research article: Alerte et réponse face aux crises: l'exemple de l'ONG Santé Diabète au Mali

**Published:** 2023-03-02

**Authors:** Stéphane Besançon

**Affiliations:** Santé Diabète, Grenoble, France

**Keywords:** Santé, Afrique, Mali, Diabète, Covid-19

Comme dans de nombreux pays en développement, le Mali connaît depuis de nombreuses années une prévalence très élevée des maladies non transmissibles dont le diabète. Ces très nombreux patients atteints de diabète sont particulièrement vulnérables en cas de crises qu'elles soient liées à des conflits ou des crises sanitaires.

Dans les 10 dernières années le Mali a connu 2 crises majeures, l'une liée au conflit de 2012 et l'autre liée à la Covid-19. Durant ces deux crises, les patients atteints de diabète n'ont pas été ciblés par les interventions des partenaires techniques et financiers. Dans ce contexte, l'ONG Santé Diabète a développé des mécanismes d'alerte et de réponses.

Après avoir mené une étude RAPIA en 2004, Santé Diabète, en collaboration avec le ministère de la Santé du Mali et les spécialistes locaux, a développé de 2005 à 2012 une stratégie globale pour lever 5 obstacles au développement de la prise en charge du diabète au Mali. C'est sur cette première construction d'une prise en charge du diabète qu'est survenu le conflit de 2012 dans le pays. Afin de répondre à cet enjeu pour les patients, nous avons lancé une alerte internationale et une réponse humanitaire au Mali. Nous présenterons cette réponse et la publication internationale issue des résultats. Ensuite nous présenterons de la même manière l'alerte et la réponse Covid-19 Diabète mise en place au Mali lors de la crise Covid en 2020.

# Research article: Surveillance par les observatoires communautaires dans l'alerte de l’émergence d’évènements sanitaires à potentiel de crise

**Published:** 2023-03-02

**Authors:** Simon Kabore

**Affiliations:** Réseau accès aux médicaments essentiels (RAME), Ouagadougou, Burkina Faso

**Keywords:** Observatoires, Sécurité sanitaire, Santé communautaire, Alerte précoce, Crises sanitaires

## Problématique

Les systèmes de santé en Afrique demeurent faibles non seulement à cause du faible investissement financier des budgets des États, mais également à cause de la verticalité des interventions sanitaires ne prenant pas suffisamment en compte les contributions des populations. Les nombreux dysfonctionnements qui les minent ne sont pas bien maîtrisés pour permettre des prises de mesures idoines.

Les observatoires communautaires sont nés dans ce contexte pour produire des évidences sur les dysfonctionnements du système de santé et orienter la prise de mesure efficace. Ils ont bénéficié de l'introduction des acteurs communautaires dans les services de santé, favorisée par la pandémie du VIH. Ils se sont révélés de véritables dispositifs d'alerte contre des ruptures de médicaments, mais aussi de violations de droits humains et de violences basées sur le genre à l'endroit de groupes vulnérables au sein des communautés.

## Interventions

La persistance des maladies à potentiel épidémique et l'apparition de la Covid-19 les ont interpellés sur un nouveau défi: la surveillance des évènements sanitaires critiques au sein des communautés. Pour cela, ils ont dû subir une mutation qui a consisté à: élargir la base des intervenants, en recrutant de nouveaux acteurs communautaires opérant dans d'autres secteurs que celui de la santé; renforcer l'ancrage communautaire en réduisant les champs d'action des organisations; renforcer les capacités sur des pathologies hors des spots de la communauté internationale; imaginer d'autres sources de motivation que les financements des bailleurs de fonds étrangers; accroître la résilience des communautés par la production communautaire de produits et matériels de protection individuels ou collectifs.

## Résultats

i) Détection précoce des crises sanitaires; ii) Renforcement de la résilience communautaire face aux crises sanitaires.

## Défis

i) Reconnaissance institutionnelle des observatoires communautaires en tant qu'outils de veille sanitaire par les systèmes de santé; ii) Appropriation par les communautés de la surveillance épidémiologique à base communautaire au détriment du financement par projet.

# Research article: Architecture mondiale de la préparation et de la riposte aux épidémies et pandémies: quel rôle pour l'Afrique?

**Published:** 2023-03-02

**Authors:** Socé Fall

**Affiliations:** Organisation mondiale de la Santé, Genève, Suisse

**Keywords:** Préparation et réponse, Épidémies, Architecture, Afrique

Le risque d’épidémies s'est accru depuis les dernières années à cause du changement climatique, de l'urbanisation rapide et des déplacements internationaux. La région africaine porte le plus lourd fardeau et compte plus de 100 urgences sanitaires par an, dont plus de 80% sont dues à des maladies infectieuses.

Les urgences sanitaires liées au climat sont en hausse dans la région, représentant plus de la moitié des évènements de santé publique enregistrés au cours des 20 dernières années. En plus de la sécheresse, des inondations et autres crises climatiques, les conflits et l'instabilité politique, comme rencontrées actuellement dans la région du Sahel et la corne de l'Afrique, augmentent de manière considérable le nombre de personnes ayant besoin d'aide humanitaire et d'assistance sanitaire.

La réponse aux épidémies nécessite des services de santé publique nationaux et locaux résilients étroitement intégrés aux soins de santé primaires et aux communautés autonomes. Il est alors essentiel, dans le cadre de la nouvelle architecture mondiale de la préparation et de la réponse aux épidémies, de renforcer les capacités aux niveaux national et local pour alerter, vérifier et répondre de manière rapide et efficace aux épidémies, particulièrement dans la région africaine. Le renforcement du système de coordination et du système d'information aux niveaux national et régional est crucial pour une meilleure gestion des épidémies et afin de permettre une meilleure prédiction et préparation aux futures épidémies, souvent récurrentes dans certaines régions. Par ailleurs, il est important de disposer dans chaque pays d’équipes multidisciplinaires, formées et prêtes à être mobilisées au niveau national. Ces équipes pourront également être déployées pour apporter un support aux autres pays de la région africaine. Un appui international spécifique pourra être considéré si nécessaire.

L'expertise et l'expérience acquises par les pays de la région africaine lors de la réponse aux épidémies majeures telles que le paludisme, la variole, la maladie à virus Zika et les maladies hémorragiques devraient être partagées et capitalisées à travers les réseaux et communautés de pratiques pour mieux faire face aux futures épidémies.

Au-delà du renforcement des capacités nationales, un investissement dans la recherche et développement permettrait plus d’équité dans l'accès aux vaccins et innovations diagnostiques et thérapeutiques en Afrique. Pour cela des mécanismes de financement durables devront être trouvés, et une solidarité régionale mise en place.

# Research article: Apports de l'imagerie cérébrale dans la compréhension et la prise en charge de la Covid longue

**Published:** 2023-03-02

**Authors:** Éric Guedj

**Affiliations:** Service de médecine nucléaire, Hôpitaux universitaires de Marseille, CHU Timone, Marseille, France

**Keywords:** Imagerie cérébrale, Covid-19, TEP, Alerte

Dix à 15% des patients touchés par la Covid-19 présentent des symptômes persistants plus de 3 mois après l'infection initiale, avec notamment de la fatigue intense, des troubles cognitifs et des troubles de l'olfaction. L'affection a été dénommée « Covid longue », et elle est maintenant reconnue par l'OMS. Cette conférence présentera les apports de l'imagerie cérébrale pour une meilleure compréhension et prise en charge de cette maladie, en montrant notamment comment ces travaux de recherche en imagerie ont permis de lancer l'alerte auprès des différentes instances nationales et internationales sur la réalité vécue par les patients dès le début de la pandémie.

# Research article: Impact de la Covid-19 sur l'accès aux soins en Afrique subsaharienne

**Published:** 2023-03-02

**Authors:** Jean-Philippe Chippaux

**Affiliations:** Institut de recherche pour le développement (IRD), France

**Keywords:** Covid-19, Impact, Santé, Offre de soins

Le nombre de cas et de décès de la Covid-19 en Afrique subsaharienne, à l'exclusion de l'Afrique du Sud qui représente près de la moitié des cas de Covid-19 notifiés, a été inférieur à ceux des autres continents pour des raisons qui restent à éclaircir. Cette étude vise à évaluer l'impact de la pandémie sur l'offre de soins et le recours à celle-ci par la population des pays d'Afrique subsaharienne (hors Afrique du Sud).

L'interrogation de PubMed^®^ avec les mots-clés « Covid, Africa, Public health, Impact » du 31 mars 2020 au 26 mars 2002, a permis d'identifier 1 151 articles. Après une sélection sur le contenu et la méthodologie, 105 articles ont été retenus. L'impact a été évalué sur la base de comparaisons avec les mois précédant l'apparition de la Covid-19 ou une saison identique les années antérieures.

Dès les premiers cas diagnostiqués, en mars 2020, une baisse significative des activités de santé touchant tous les secteurs, souvent associée à une réduction de la qualité des soins, et la fermeture de services ont été rapportées. Certaines activités ont été particulièrement touchées comme les vaccinations de routine, la santé maternelle et reproductive, les dons de sang, le dépistage du VIH et la distribution des antirétroviraux, la prévention de la rage, les programmes de contrôle des maladies endémiques, notamment la rougeole, la tuberculose, le paludisme et les maladies tropicales négligées. La distribution de médicaments à large échelle et de moustiquaires imprégnées ainsi que les interventions de santé publique ont été interrompues dans de nombreux pays, faisant courir le risque d'une recrudescence de ces maladies. L'approvisionnement en produits de santé et leur utilisation ont été profondément perturbés à cause des restrictions de transport ou de reports de consultation entraînant des effets néfastes sur les maladies chroniques – hypertension, diabète, cancers – et des ruptures de stocks. La démotivation ou la démoralisation du personnel de santé ont été rapportées. Une vulnérabilité particulière a été observée chez les handicapés, les PVVIH, les habitants de bidonvilles, les personnes déplacées et les professionnels du sexe.

Cependant, l'impact sur les prestations de santé a été très hétérogène selon les localités – plus élevé en milieu urbain que rural – avec une forte majoration les premiers mois de la pandémie, puis en fonction des vagues épidémiques qui n'ont pas été synchrones dans les différents pays. L'accès aux structures de santé a été considérablement réduit en raison des mesures de confinement et de restriction des transports prises par les autorités, du manque de ressources humaines et matérielles, notamment les équipements de protection individuelle. L'augmentation des coûts, s'ajoutant à l'appauvrissement de la population, la peur d’être contaminé et diagnostiqué positif ou stigmatisé ont également dissuadé les patients de se rendre dans les centres de santé. Plusieurs études soulignent une reprise progressive entre les vagues épidémiques et une certaine capacité d'adaptation de l'offre de soins, voire une bonne résilience des services de santé ou des patients eux-mêmes.

Quelques articles formulent des recommandations visant à atténuer l'impact des épidémies: mise en œuvre précoce des interventions, développement de stratégies d'information appropriées, régulation des flux de patients, approvisionnement en produits de santé, notamment en équipements de protection individuelle, formation du personnel de santé à l'accueil et à la prise en charge des patients, amélioration des techniques diagnostiques et thérapeutiques alternatives comme la dispensation appropriée des médicaments, l'utilisation d'autotests, de drones ou de la télémédecine, organisation du rattrapage des vaccinations de routine et adaptation de la législation sur les violences familiales.

# Research article: Organisation des soins au cours de l’épidémie de peste pulmonaire urbaine à Madagascar: cas du CHU Joseph Raseta Befelatanana

**Published:** 2023-03-02

**Authors:** Mamy Randria

**Affiliations:** Service des maladies infectieuses, CHU Joseph Raseta Befelatanana, Antananarivo, Madagascar

**Keywords:** Épidémie, Peste, Soins, Prévention

Madagascar est un pays endémique de la peste mais pour la première fois en 2017, une épidémie de peste pulmonaire sévissait en milieu urbain dans les deux plus grandes villes du pays.

Le CHU Joseph Raseta Befelatanana se trouvant dans la capitale est un grand hôpital à vocation médicale de référence nationale où se trouvent les spécialités médicales. Il était l’épicentre de la gestion des cas de peste lors de l’épidémie. Par ailleurs, il devait aussi assurer la prise en charge des autres patients non pesteux. Pour assurer les soins et la sécurité des patients, le circuit des patients a été réorganisé au sein de l'hôpital. Ce circuit comprenait un pré-triage, deux triages et des secteurs d'hospitalisation distincts (cas suspect et confirmé de peste, non-cas).

Tous les patients passaient obligatoirement au niveau du pré-triage, tous les cas suspects et confirmés de peste ayant rempli la définition des cas suivaient le circuit prédéterminé. Les non-cas prenaient le circuit habituel. L'ensemble du personnel soignant a été formé sur le diagnostic et le traitement de la peste et sur les mesures de prévention et contrôle des infections axées plus spécifiquement sur la peste pulmonaire. Au niveau des secteurs de traitement et d'isolement des cas de peste, les soins étaient assurés par des équipes formées et portant des équipements de protection individuelle adaptés. Ces équipes étaient essentiellement composées par les soignants de l'hôpital même qui participaient tous aux soins selon les tours de garde établis. La prise en charge des enfants était assurée par les équipes de la pédiatrie. Un avis spécialisé (infectiologue ou pneumologue) était en permanence possible. L'obtention rapide de la confirmation du diagnostic était cruciale pour l'acceptation de la prise en charge par les patients et pour la gestion des cas de décès sans conflit avec les familles.

La prise en charge des patients non infectés se poursuivait normalement dans tous les services. Les visites étaient interdites dans tous les secteurs de l'hôpital pour éviter une éventuelle contamination des malades non pesteux par les visiteurs. Une adaptation et une amélioration permanentes de l'organisation mise en place se sont avérées nécessaires. L'aspect logistique a également tenu une place importante: extension rapide des secteurs de soins, gestion des équipements de protection individuelle et de la chimioprophylaxie.

# Research article: Accès aux structures de santé en contexte épidémique: l'exemple de l’épidémie de maladie à virus Ébola en Afrique de l'Ouest, 2013-2016

**Published:** 2023-03-02

**Authors:** René Migliani

**Affiliations:** Professeur agrégé du Val-de-Grâce, Université de Bordeaux II, Bordeaux, France

**Keywords:** Ébola, Recours aux soins, Épidémie, Afrique de l'ouest, Crise sanitaire

L’épidémie de maladie à virus Ébola, entre son émergence en 2013 en Guinée et sa fin officielle en avril 2016, a été historiquement la plus grave. Elle a touché quasi exclusivement 3 pays d'Afrique de l'Ouest: la Guinée, le Libéria et la Sierra Leone. Au total, 28 616 cas ont été notifiés dont 11 310 (39,5%) sont décédés.

Inconnue en Guinée, cette grave infection dont l’étiologie n'est pas comprise va influer négativement sur l'adhésion aux mesures préventives qui se heurtent aux coutumes traditionnelles. En effet, elle fait peur aux communautés et aux soignants qui vont payer un lourd tribut. Les patients et des soignants de première ligne vont fuir les structures de santé qu'ils perçoivent comme des lieux concentrant l’épidémie. La baisse du recours aux soins va dépasser 50% pour les consultations et hospitalisations en Guinée en 2014, en étant plus marquée en zone urbaine. Cette baisse a concerné toutes les activités médicales dont la lutte contre les principales endémies (paludisme, tuberculose, infections à VIH-sida, maladie du sommeil), les activités maternelles et infantiles (surveillance des grossesses, accouchements et vaccinations des enfants), et la prise en charge des maladies graves comme les cancers et les urgences.

L'impact sanitaire indirect de l’épidémie va être supérieur à son impact direct en termes de mortalité. Dans les 3 pays les plus touchés le nombre de décès supplémentaires, estimé pour seulement 4 maladies infectieuses d'importance (tuberculose, VIH-sida, rougeole, paludisme), égal à 18 791, est supérieur de 40% au total du nombre de décès liés à la MVE. Par ailleurs la mortalité maternelle pour 100 000 naissances vivantes (NV) a augmenté de 11 à 74% selon le pays par rapport à la période pré-épidémique et celle des enfants de moins de 5 ans pour 1 000 NV de 10 à 119%.

Ce sont avant tout les difficultés de communication sur les risques avec les populations et les soignants, qui ont contribué à l'ampleur et la gravité de cette épidémie et au fort coût sanitaire indirect par défaut de recours aux soins. En plus des stratégies de contrôle bien codifiées maintenant, auxquelles s'est ajoutée la vaccination anti-Ébola, il est fondamental de placer au centre de la gestion de crise la communication par une écoute systématique des communautés et d'intégrer davantage le maintien de la capacité à fournir des soins médicaux curatifs et préventifs pour les personnes non atteintes lors des épidémies à virus Ébola.

# Research article: Accès aux soins et lutte contre le choléra lors de l’épidémie d'Haïti en 2010

**Published:** 2023-03-02

**Authors:** Renaud Piarroux

**Affiliations:** CHU de la Pitié-Salpêtrière, Faculté de médecine Sorbonne Université, Paris, France

**Keywords:** Choléra, Accès aux soins, Épidémie, Système de santé, Haïti

Assurer l'accès aux soins à un maximum de personnes est la première mesure à prendre en cas d’épidémie. En contexte d'urgence, cela permet de sauver un maximum de vies. Parallèlement, il importe de mettre en place des mesures limitant la propagation de l’épidémie. Enfin, il est nécessaire de veiller à ce que l’épidémie et sa prise en charge ne constituent pas un obstacle supplémentaire à l'accès aux soins pour les autres pathologies. Ce problème était d'autant plus complexe à gérer en Haïti, qu'il s'agit d'un des pays les plus pauvres et inégalitaires du monde, avec 59% de la population vivant sous le seuil de pauvreté.

Nous présentons ici un retour d'expérience sur la situation sanitaire d'Haïti et sur la gestion de l’épidémie de choléra qui frappa ce pays 9 mois après un tremblement de terre extrêmement destructeur, responsable de 100 000 à 300 000 décès. L’épidémie de choléra fut marquée par une première vague d'une intensité exceptionnelle (avec un pic de contamination de plus 30 000 cas diagnostiqués en une seule semaine), suivie de vagues saisonnières rythmées par les pluies. Ce n'est qu'après 6 ans, avec l'intensification des mesures prises pour casser les chaînes de transmission autour des cas, que l’épidémie put enfin être contrôlée, avant d’être totalement stoppée 2 ans plus tard. La phase initiale explosive de l’épidémie a été caractérisée par un accès aux soins très inégal, où de grands centres de prise en charge dans les zones urbaines contrastaient avec l'absence totale de structures de soins dans de nombreuses zones rurales, résultant en une très forte mortalité. L'installation d'unités de traitement plus légères et plus facilement déployables a ensuite permis d'améliorer l'accès aux soins. Cela a grandement facilité la mise en place d'une stratégie de lutte basée sur l'interruption de la transmission autour des cas par la sécurisation de l'approvisionnement en eau et le traitement prophylactique des sujets contacts. La raréfaction du nombre de foyers épidémiques à partir de 2017 a rendu possible le renforcement de la stratégie d’élimination, finalement couronnée de succès début 2019. Tout au long du suivi de l’épidémie et du contrôle de la lutte, les missions menées sur le terrain ont montré combien l'accès aux soins était limité pour une frange importante de la population, non seulement dans les bidonvilles des grands centres urbains, mais aussi, et surtout, dans les zones rurales isolées, en particulier dans les mornes.

# Research article: Accès aux soins dans le contexte de l’épidémie de Covid-19: le cas du Sénégal

**Published:** 2023-03-02

**Authors:** Babacar Ndiaye

**Affiliations:** Institut Pasteur de Dakar, Dakar, Sénégal

**Keywords:** Covid-19, Soins, Sénégal

## Introduction

L’épidémie au SARS-CoV-2 déclarée comme urgence mondiale par l'OMS s'est rapidement propagée dans le monde avec une mortalité très élevée, mettant à rude épreuve les systèmes de santé mondiaux.

Les leçons apprises des précédentes épidémies telles que la fièvre jaune, Ébola et le choléra ont servi au continent africain à montrer une capacité de résilience importante vis-à-vis de la pandémie à Covid-19.

Les mesures prises (confinement, restriction des déplacements), la peur et la stigmatisation engendrées par la Covid-19 ont eu des effets considérables sur l'efficacité du système de soins au Sénégal.

## Méthodologie

Nous avons recueilli les informations issues des rapports officiels du ministère de la Santé (MSAS) et des organisations internationales, les enquêtes de l'Agence nationale de la statistique et de la démographie (ANSD) et les publications portant sur l'offre de soins au Sénégal depuis le début de l’épidémie au Sénégal (mars 2020) jusqu’à nos jours.

Nous avons étudié la prise en charge des maladies chroniques ou infectieuses, la santé mère-enfant, le diagnostic paraclinique ainsi que la disponibilité des produits et dispositifs médicaux nécessaires à une prestation sanitaire de qualité.

## Résultats

Les mesures prises pour limiter la propagation du virus ont eu d'importantes répercussions sur le suivi des patients dans les structures spécialisées et en zones rurales. Ainsi, il a été observé entre autres: i) une diminution de la fréquentation des structures de santé de la reproduction et en pédiatrie; ii) des bouleversements du suivi des maladies chroniques ou infectieuses; iii) des reprogrammations d'interventions chirurgicales; iv) une diminution de l'activité des services de diagnostic paraclinique; v) des ruptures de certains produits pharmaceutiques essentiels.

Face à cette situation, d'importantes mesures ont été prises: i) le renforcement des hôpitaux en termes de logistique; ii) le déploiement d'hôpitaux de campagne; iii) une dotation multi-mois en ARV pour les patients vivants avec le VIH; iv) une interdiction de l'automédication aux produits pharmaceutiques jugés efficaces contre la Covid-19.

## Conclusion

L'Afrique, malgré ses moyens limités, a montré une forte capacité de résilience face à la pandémie à Covid-19. Le Sénégal a pu obtenir des résultats probants grâce à des stratégies diverses et des innovations remarquables. Ceci doit être pérennisé afin de renforcer la résilience du système de santé en cas de nouvelles crises sanitaires.

# Research article: Évaluation des performances de l'utilisation du District Health Information Software 2 (DHIS 2) par les formations sanitaires de la ville de Maroua au Cameroun

**Published:** 2023-03-02

**Authors:** Lea Melataguia Mekontchou, Julienne Ekodi, Pierre René Binyom, Chanceline Bilounga Ndongo, Andy Michael Mbesse Njie, Danielle Laure Abada, Mathieu Ari, Yvan Paul Deffo, Boniface Ngangoue, Patricia Mendjime, Armel Evouna Mbarga, Linda Esso Endall, Georges Alain Etoundi Mballa, Peter Ebongue Mbondji, Magloire Biwole Sida, Georges Nguefack-Tsague

**Affiliations:** Faculté de médecine et des sciences biomédicales, Université de Yaoundé I, Cameroun

**Keywords:** Performance, DHIS2, Formation sanitaire, Maroua

## Introduction

Le Système d'information sanitaire (SIS) est un processus de recueil de l'information sanitaire au niveau périphérique, régional et central du système de soins, son stockage, son traitement pour la production de résultats et enfin sa transmission vers des organismes habilités à la prise de décision. Au Cameroun, le ministère de la Santé publique a adopté le District Health Information Software 2 (DHIS2) comme outil numérique de gestion des données du SIS. L'objectif de cette étude était d'identifier les différents facteurs pouvant être associés à la performance des formations sanitaires (FOSA) de la ville de Maroua quant à l'utilisation du DHIS2.

## Méthodes

Il s'agissait d'une étude transversale analytique conduite entre avril et mai 2021 dans les FOSA de la ville de Maroua. Les données ont été collectées à l'aide d'un questionnaire structuré et analysées avec le logiciel STATA 14. La régression logistique multiple a été utilisée afin de déterminer les facteurs associés à la performance des FOSA. Les valeurs p inférieures à 0,05 ont été considérées comme statistiquement significatives.

## Résultats

Sur les 42 FOSA enquêtées, 28 (66,67%) étaient du secteur public, et 31 (73,81%) des centres de santé. L’âge des répondants variait entre 20 ans et 55 ans; 76,19% (32) étaient des hommes; 42,50% (17) des aides-soignants; et 50% (20) des infirmiers. Les FOSA ayant une mauvaise performance représentaient 59,52% (25); dont 78,75% chez les FOSA gérées par les hommes (p = 0,029). Les autres facteurs associés à la mauvaise performance des FOSA étaient le manque de formation (RC = 10,66; IC = (1,21; 93,69); p = 0,007); l’électrification non permanente (RC = 13,09 (1,40; 122,23); p = 0,006); les connaissances sur le DHIS2 (RC = 2,8 (0,7; 11,08); p = 0,013); la rédaction des messages dans le DHIS2 (RC = 7,5 (1,77; 31,68); p = 0,004); et l'analyse des données sur les programmes prioritaires (RC = 5,71 (1,44; 22,62); p = 0,01).

## Conclusion

Les FOSA de la ville de Maroua témoignent de mauvaises performances quant à l'utilisation du DHIS2. Il serait souhaitable de programmer des séances de formation et des supervisions afin de renforcer les capacités du personnel gestionnaire de données DHIS2.

# Research article: Analyse de la répartition spatiale des cas de Covid-19 au Bénin

**Published:** 2023-03-02

**Authors:** Marc Fiogbé, Gilles Cottrell, Aurore Atchade, Emmanuel Yovo, Daleb Abdoulaye Alfa, Sandrine Hounsa, Jean-Yves Le Hesran, Agnès Yadouleton, Raquel Gonzalez, Jean Gaudart, Cédric Bationo, Achille Massougbodji, Emmanuel Bonnet

**Affiliations:** Institut de Recherche clinique du Bénin, 04 BP 1114 Abomey-Calavi, République du Bénin

**Keywords:** Covid-19, Distribution spatiale, Hot spots, Bénin

## Contexte

Depuis mars 2020 le Bénin, à l'instar du reste de l'Afrique subsaharienne et d'autres régions du monde, fait face à une situation de crise sanitaire due à la pandémie de Covid-19. Pour y répondre, le Bénin a opté pour des mesures de ripostes adaptatives en fonction de l’évolution de la pandémie. Le projet STREESCO s'inscrit dans cette dynamique en renforçant la surveillance épidémiologique sur trois sites sentinelles au Bénin. Nous présentons ici les résultats préliminaires issus de l'analyse de la répartition spatio-temporelle des cas de Covid-19.

## Méthodes

L'analyse de la dynamique spatiale des cas Covid-19 s'est effectuée en 2 phases. Une première phase a consisté en la mise en place d'un dispositif de surveillance épidémiologique sur trois sites sentinelles, à savoir Cotonou (capitale économique), Allada (ville secondaire au Sud) et Natitingou (ville située dans le Nord du pays avec une forte connexion avec les pays de l'Hinterland). Il s'agissait en sus des données socio-démographiques de recueillir des données sur le quartier de résidence, l’état de santé, notamment le statut virologique de cas suspects, cas contacts et agents de santé impliqués dans la lutte contre la Covid-19. Une première analyse de la distribution spatiale des cas de Covid-19 a été cartographiée.

Dans un second temps, des analyses spatio-temporelles ont été réalisées en utilisant la statistique de balayage spatial implémentée dans SatScan par Kulldorff. Cette méthode permet de détecter les régions où l'incidence de la maladie est plus élevée que prévu dans le temps et l'espace en leur attribuant un risque relatif, produisant ainsi une liste de clusters spatio-temporels qui peuvent être utilisés pour identifier les phases épidémiques dans la zone d’étude.

## Résultats

La surveillance épidémiologique mise en place a permis d'analyser des données de 4 178 participants répartis sur les trois sites comme suit: Cotonou (2 188), Allada (1 036), Natitingou (954). L'analyse spatiale fait ressortir une distribution spatiale des cas Covid-19 différente par site. On note notamment à Cotonou des clusters de cas positifs.

## Conclusion

Ces analyses mettent en évidence une incidence des cas Covid-19 variable en fonction des zones géographiques. Ces analyses méritent d’être approfondies en complétant cette zone géographique avec les données des autres sites de dépistage. Une analyse des facteurs de risque de la Covid-19 en lien avec la localisation géographique permettra de mieux apprécier la distribution spatiale des cas Covid-19 au Bénin.

# Research article: La surveillance nationale de la variole du singe en République centrafricaine, 2001-2021

**Published:** 2023-03-02

**Authors:** Camille Besombes, Festus Mbrenga, Ella Gonofio, Jordi Landier, Antoine Gessain, Arnaud Fontanet, Emmanuel Nakouné

**Affiliations:** Unité d’épidémiologie des maladies émergentes, Institut Pasteur, 25-28 rue du Docteur Roux, 75015 Paris, France

**Keywords:** Investigation d’épidémie, Système de surveillance nationale, Variole du singe, Monkeypox, Afrique centrale

## Contexte

La variole du singe est une zoonose survenant dans les zones forestières d'Afrique centrale et d'Afrique de l'Ouest, avec une présentation clinique similaire à la variole et une mortalité élevée. La description des manifestations épidémiologiques et cliniques de la variole du singe reste rare, d'autant plus en République centrafricaine (RCA) qui est pourtant le quatrième pays le plus concerné par cette zoonose (Bunge EM *et al.*, 2022). Nous rapportons ici l'analyse des données recueillies par le système national de surveillance mis en place en RCA depuis 2001.

## Matériel et méthodes

La surveillance nationale de la variole du singe en RCA est basée sur un système de formation continue du personnel des centres de santé et sur un système d'alerte national: chaque cas suspect de variole du singe est notifié à l'Institut Pasteur de Bangui, le centre de référence régional. Lorsqu'un cas de variole du singe est confirmé par PCR, une équipe d'investigation est envoyée sur le terrain pour mener l'enquête épidémiologique et clinique chez l'humain et une investigation des potentiels réservoirs animaux, selon une approche « Une seule santé » (One Health). Les données épidémiologiques, cliniques et virologiques obtenues par la surveillance nationale de la variole du singe en RCA ont été analysées pour étudier la fréquence et les caractéristiques des épidémies ainsi que la gravité clinique de la maladie.

## Résultats

Quatre-vingt-quinze épidémies suspectes de variole du singe ont été signalées en RCA entre 2001 et 2021, dont 40 ont été attribuées à la variole du singe, 32 à la varicelle et 23 n'ont pas eu d’étiologie confirmée. Le nombre d’épidémies est en augmentation depuis 2018, hormis une baisse transitoire en 2020, avec une taille d’épidémie variant sur l'ensemble de la période de 1 à 13 pour les cas confirmés, aucun foyer ne dépassant 25 cas (cas confirmés et suspects). Deux régions forestières étaient principalement concernées, la Lobaye et le Mbomou (respectivement 40% et 25% des épidémies). L’âge médian (IQR) des 99 cas confirmés était de 15,5 (5,5-28) ans, avec seulement 2 cas nés avant 1980, et donc vraisemblablement vaccinés contre la variole. Le taux de létalité était de 12,1% (12/99), atteignant 17,4% (8/46) chez les enfants. Parmi les 498 sujets ayant des résultats sérologiques, 342 (68,5%) étaient positifs pour les IgG (immunoglobulines) anti-monkeypox virus.

## Conclusion

L'augmentation récente du nombre d’épidémies de variole du singe en RCA doit être considérée avec prudence, compte tenu de la diminution de la couverture vaccinale contre la variole et de la létalité élevée associée à cette maladie, en particulier chez les enfants. Une meilleure compréhension des sources zoonotiques d'infection et des environnements concernés pourrait aider à concevoir des mesures de prévention adaptées.

# Research article: Identification des villages à risque (IVR) de Trypanosomiase humaine africaine: une approche géographique pour évaluer le risque de réémergence de la THA dans les foyers historiques

**Published:** 2023-03-02

**Authors:** Jérémi Rouamba, Bamoro Coulibaly, Pacôme Adingra, Colette Kouakou, Adama Ouattara, Débora Krouba, Ramadan Kalki, Mamady Camara, Dramane Kaba, Dr. Lingue Kouakou, Mallaye Peka, Vincent Jamonneau, Philippe Solano, Fabrice Courtin

**Affiliations:** Institut de recherche pour le développement (IRD), 688 avenue Joseph Ki-Zerbo, Ouagadougou, Burkina Faso

**Keywords:** Histoire, Géographie, Afrique, Trypanosomiase, Risque

La Trypanosomiase humaine africaine (THA) est une maladie tropicale négligée due à un trypanosome, transmis à l'homme lors du repas sanguin d'une mouche tsé-tsé infectée. Durant les xix^e^ et xx^e^ siècles, elle a constitué un problème majeur de santé publique qui a nécessité la création de services de lutte spécifiquement dédiés. D'un peu plus de 25 000 cas en 2000 à moins de 1000 cas dépistés en 2020, la THA est en passe d’être éliminée comme problème de santé publique, tel que souhaité par l'OMS. Cependant, certains foyers historiques (parfois à l’échelle d'un pays) où la mouche tsé-tsé est présente, n'ont pas été investigués parfois depuis plus de 30 ans, comme par exemple en Sierra Leone, au Libéria et en Gambie. La méthode IVR (Identification des villages à risque) est basée sur une approche géographique du système pathogène de la THA, qui permet, via la construction d'un Système d'information géographique alimenté par des données historiques, géographiques, entomologiques et parasitologiques, d'avoir un aperçu de la situation de la THA, et d’évaluer le risque de réémergence, sur une grande superficie. Les résultats obtenus fournissent des éléments utiles pour la mise en place d'un système de surveillance épidémiologique ciblé, permettant de détecter tout risque de réémergence en vue d'atteindre l'objectif d’élimination. Cette communication orale vise à présenter la méthode en ellemême, ainsi que les résultats obtenus par l'application de cette méthode dans les pays investigués: Burkina Faso, Côte d'Ivoire, Guinée, Sénégal, Guinée-Bissau, Niger, Tchad.

# Research article: Un système d'information pour la recherche interventionnelle en milieu tropical isolé: exemple de l’étude Malakit

**Published:** 2023-03-02

**Authors:** Yann Lambert, Muriel Galindo, Alice Sanna, Louise Mutricy, Stephen Vreden, Martha Suarez-Mutis, Maylis Douine

**Affiliations:** Centre d'investigation clinique Antilles-Guyane, INSERM 1424, Centre hospitalier de Cayenne Andrée-Rosemon, Cayenne, France

**Keywords:** Données mobiles, Amazonie, Paludisme, Orpaillage

## Contexte

En Guyane, la population travaillant sur les sites d'orpaillage est la plus touchée par le paludisme. Sa mobilité importante sur le plateau des Guyanes et son recours fréquent à une automédication inadaptée font craindre l’émergence et la diffusion régionale de parasites résistants. Conçue comme une réponse à un défaut d'accès aux soins en situation isolée, Malakit est une intervention innovante reposant sur la réalisation du diagnostic et du traitement du paludisme par les orpailleurs eux-mêmes à l'aide d'un kit et d'une formation initiale assurée par des médiateurs.

## Méthodes

L'intervention Malakit a été évaluée entre avril 2018 et mars 2020 et mise en place sur 5 sites situés aux frontières de la Guyane avec le Brésil et le Suriname, imposant de fortes contraintes au système d'information de l’étude: isolement en forêt amazonienne, climat tropical, absence d'alimentation électrique et de connexion Internet fiables, multilinguisme et multiculturalité. Le système d'information de Malakit a été développé comme un « patchwork » de logiciels libres existants, de services commerciaux et d'outils développés en interne. La collecte des données des participants par les médiateurs a reposé sur l'application ODK Collect via des tablettes Android. Un package R et un tableau de bord ont été développés pour télécharger, déchiffrer, agréger, suivre et nettoyer les données selon les retours des médiateurs et de la supervision de terrain.

## Résultats

Entre avril 2018 et mars 2020, neuf médiateurs ont saisi un total de 4 863 formulaires, soit une moyenne de 202 enregistrements par mois. Les retours et commentaires libres des médiateurs ont été essentiels pour adapter et améliorer la collecte et le suivi des données mobiles. Peu de problèmes techniques ont été signalés. La saisie électronique par un médiateur durait en médiane 5 minutes, permettant une économie de temps des participants au profit de leur formation à l'utilisation du kit. La qualité des données était satisfaisante avec seulement 3% des enregistrements nécessitant une correction.

## Conclusions

Le développement du système d'information du projet Malakit a été une source d'innovation, à l'image de l'intervention elle-même. Cette expérience confirme qu'il est possible de produire des données de qualité et d’évaluer une intervention de santé publique en adaptant les outils aux contraintes du terrain et à l'expérience des médiateurs.

# Research article: Épidémies de Covid-19 en Guyane de mars 2020 à mai 2022: des variants, des vaccins et des peuples

**Published:** 2023-03-02

**Authors:** Loïc Epelboin, Mélanie Gaillet, Céline Michaud, Margot Oberlis, Estelle Jacoud, Nelly Lupo, Evrard Baduel, Florence Huber, Bastien Bidaud, Fredrik Terlutter, Jean-Yves Cattin, Guillaume Icher, Sophie Robin, Pedro Clauteaux, Cyril Rousseau, Félix Djossou, Nicolas Vignier

**Affiliations:** Unité des maladies infectieuses et tropicales, CIC INSERM 1424, Centre hospitalier de Cayenne Andrée-Rosemon, Cayenne, France

**Keywords:** Guyane, Amazonie, Covid-19, Vaccinosepticisme

La Guyane, territoire ultramarin situé en Amérique du Sud, est la région de France avec la couverture vaccinale contre la Covid-19 la plus faible de France.

Les épidémies qui ont successivement touché ce territoire amazonien ont différé de l'Hexagone par de multiples aspects: épidémies décalées, virus différents, atteinte hétérogène sur le territoire, mesures sanitaires differentes parfois « innovantes » (interruption des vols intérieurs et vers les pays voisins, limitation des vols outre-Atlantique, fermeture des frontières, confinement localisé ou généralisé, couvre-feu à géométrie variable, etc.), le tout dans un territoire multiculturel et multiethnique avec l'un des taux de précarité les plus élevés du pays.

Dans ce contexte, l'arrivée de la vaccination qui devait représenter la porte de sortie a au contraire été l'objet de toutes les polémiques. Localement, les autorités de santé et les soignants ont multiplié les interventions et les actions de façon à trouver des solutions pour acheminer les vaccins vers les lieux les plus reculés de la forêt amazonienne, tout en convainquant les multiples communautés qui constituent la Guyane de l'intérêt de cette vaccination.

La conviction ou au contraire l'hésitation vaccinales ont pris de multiples formes selon les lieux et les communautés, du littoral aux communes de l'intérieur, du fleuve Oyapock, à la frontière brésilienne, au Maroni, à la frontière surinamaise, des « Métros » aux Amérindiens, des Créoles aux Hmongs, des Bushinengués aux Brésiliens, etc.

Dans la mêlée se sont retrouvés les autorités sanitaires et administratives, les soignants, les associations, les médiateurs en santé, les épidémiologistes, les mouvements autonomistes, les mouvements antivax, les politiques guyanaise et hexagonale, aboutissant malgré tout cela à une ambiance plutôt moins délétère qu'aux Antilles, mais avec une couverture vaccinale la plus faible de France.

Ces phénomènes peu médiatisés dans l'Hexagone ont profondément bouleversé la société guyanaise. Nous vous proposons ici une présentation des déterminants de l’épidémie et de la vaccination Covid-19 en Guyane de mars 2020 à mai 2022.

# Research article: Une communication fondée sur les représentations de la population

**Published:** 2023-03-02

**Authors:** Bernard Seytre

**Affiliations:** bnscommunication, Paris, France

**Keywords:** Covid-19, Communication, Représentations

En santé publique, une communication inadaptée est souvent à l'origine d'incompréhensions et de doutes, lesquels sont susceptibles de générer méfiance, voire hostilité. Or, gagner la confiance de la population est essentiel pour l'amener aux changements de comportement souhaités en période de crise sanitaire, comme généralement pour toute question de santé publique.

Nous fournirons des exemples d'une approche de « culture en santé », qui se fonde sur les représentations de la population sur la question de santé concernée pour proposer une stratégie, un plan et des outils de communication adaptés. Nous présenterons notamment un travail mené en Afrique de l'Ouest pour l'Organisation ouest africaine de la Santé visant à renforcer l'adhésion de la population aux mesures de prévention de la Covid-19. Nous avons conduit dans 5 pays une enquête socio-anthropologique sur les représentations de la population, puis proposé une stratégie de communication et produit un ensemble d'outils de communication sur la base des résultats de cette enquête.

Nous questionnerons la pertinence des études sur l’« infodémie » centrées sur la production et la circulation en ligne d'informations fausses sur la Covid-19 et qui tracent implicitement ou explicitement un trait d’égalité entre cette circulation et les représentations de la population.

# Research article: La place de l'information dans la prise en charge des maladies tropicales négligées: cas des envenimations par morsures de serpent dans le District de Bokoro en République démocratique du Congo

**Published:** 2023-03-02

**Authors:** Jacques Ngambongo Mopili, Sophie Bokata, Pr Hyppolyte Situakibanza, Thaddée Odio

**Affiliations:** Centre antivenimeux/Ministère de la Recherche scientifique, Kinshasa, République démocratique du Congo

**Keywords:** Information, Prise en charge, Maladies négligées, Envenimation, République démocratique du Congo

## Introduction

En République démocratique du Congo, l'incidence et la gravité des morsures de serpents restent encore mal connues. Les études réalisées sur ce sujet sont rares et se limitent à quelques centres hospitaliers et ménages où la létalité annuelle varie autour de 5%. Le manque de formation du personnel de santé et de sensibilisation de la population sur les morsures de serpent et l'absence d'une sérothérapie antivenimeuse, est la principale difficulté rencontrée dans ce pays; et aussi l'un des facteurs qui explique le nombre élevé de mortalité et d'incapacités. Le district de Bokoro, dans la province du Mai-Ndombe, enregistre souvent des morsures suivies de décès. L'usage du garrot à la place de la bande Velpeau, de la pierre noire, de l'incision et d'autres pratiques tendant à aggraver la situation sont courants dans la contrée. Notre intervention (équipe du Centre antivenimeux) a consisté à former le personnel soignant et à sensibiliser la population sur la prévention et l'amélioration de la prise en charge des cas d'envenimations par morsures de serpent avec un lot de 50 flacons de sérum antivenimeux, démarche qui n'a officiellement pas eu lieu depuis 1960.

## Matériel et méthodes

Une étude rétrospective puis prospective auprès de la population et du personnel soignant des Cliniques universitaires de Mai-Ndombe à Bokoro a été réalisée entre le 10 janvier et le 12 avril 2021. L’étude a consisté en une approche comparative entre la période avant et après l'intervention de l’équipe formatrice afin de mesurer l'impact de la formation et de la sensibilisation sur la prise en charge des mordus, sur la base d'un questionnaire administré.

## Résultats

Sur un total de 151 mordus: Avant l'enquête: 46 morsures répertoriées avec 13 décès (28,26%); 89,13% ont utilisé la pierre noire, 98,02% le garrot, 63,04% l'incision, 67,39% la succion et 26,09% l'urine.

Après formation et sensibilisation: 105 mordus dont 15 (14,29%) décès; 29 (27,6%) patients ont utilisé la pierre noire, 30,48% le garrot, 6,7% l'incision, 2,86% la succion et l'urine n'a pas été utilisée.

## Conclusion

Cette étude établit de manière évidente que la sensibilisation de la population et la formation du personnel médical revêtent une importance non négligeable quant à l'amélioration de la prise en charge des personnes mordues par le serpent. Ladite étude encore parcellaire mérite d’être poursuivie dans toutes les provinces afin de diminuer très sensiblement les décès et les incapacités.

# Research article: Risque compétitif de la mortalité sur la mise sous traitement anti-tuberculeux parmi les patients hospitalisés pour tuberculose neuro-méningée dans le service des maladies infectieuses du Centre hospitalier et universitaire de Brazzaville, République du Congo

**Published:** 2023-03-02

**Authors:** Herbas Martin Ekat, Bienvenu Rolland Ossibi Ibara, Tatia Adoua Doukaga, Dr Kosso Angello, Axel Aloumba Gillius, Médard Amona, Ella Angonga, Princesse Bendent, Fred Kinga

**Affiliations:** Service des maladies infectieuses, CHUB, Brazzaville, République démocratique du Congo

**Keywords:** Traitement, Mortalité, Tuberculose neuro-méningée, VIH, Congo

## Objectif

Le diagnostic de la tuberculose neuro-méningée reste difficile dans les pays où le plateau technique est limité. La mortalité est importante entre l'admission dans les unités de prise en charge et le moment de la prise de décision de démarrer le traitement anti-tuberculeux. Cette étude cherche à déterminer le taux de mise sous traitement anti-tuberculeux chez les patients hospitalisés pour tuberculose neuro-méningée (TNM) dans le service des maladies infectieuses du Centre hospitalier et universitaire de Brazzaville, République démocratique du Congo.

## Patients et méthodes

Il s'est agi d'une étude de cohorte rétrospective. Les dossiers des patients (âgé de 20 ans et plus) hospitalisés pour TNM dans le service des maladies infectieuses du Centre hospitalier et universitaire de Brazzaville de mars 2014 à mars 2019 ont été colligés. Conformément aux schémas diagnostiques établis par la British Infection Society, le diagnostic de TNM a été retenu devant les arguments épidémiologiques, cliniques, biologiques et morphologiques. Toute méningite lymphocytaire associée à la mise en évidence du BK dans tout autre liquide biologique que le LCR et/ou associée à des anomalies radiologiques pulmonaires a été considérée comme TNM. Le critère de jugement principal est la mise sous traitement anti-tuberculeux, le décès avant le début du traitement anti-tuberculeux étant considéré comme critère de jugement secondaire. Les facteurs associés à la mise sous traitement anti-tuberculeux ont été identifiés avec le modèle de risque compétitif.

## Résultats

Au total 48 patients étaient inclus, dont 66,67% étaient de sexe féminin, l’âge médian était de 42 ans (intervalle interquartile (IIQ): 34,5-51,5). 20,8% avaient des antécédents de tuberculose et 85,11% des patients étaient VIH positifs. Après une durée d'hospitalisation médiane de 5 jours (IIQ: 2,5-9), 41 (85,42%) sur les 48 patients avaient été mis sous traitement soit une durée de participation de 291 personnes-année, et l'incidence de la mise sous traitement était de 14,09 pour 100 personnes-année (10,37-19,14). Au cours de l'hospitalisation, 14,58% des patients étaient décédés avant d’être mis sous traitement anti-tuberculeux. L'ajustement à l’évènement compétitif (le décès) augmente indépendamment l'incidence de mise sous traitement anti-tuberculeux chez les patients ayant le score de Glasgow entre 5 et 7 (aSHR: 4,51 (IC95%: 2,11-9,64), p < 0,001). Conclusion. Dans les pays à ressources limitées, la présence d'un coma stade 3 permet de débuter le traitement chez les patients suspects de tuberculose neuro-méningée et de continuer les investigations.

# Research article: Installation de laboratoires de campagne (LABOMEDCAMP) au Mali: premier bilan et pérennisation

**Published:** 2023-03-02

**Authors:** >Moussa Karim Goita, Madine Tall, Nouhoum Bouare, Abderrhamane Maiga, Mansour Sy, Odile Ouwe Missi Oukem-Boyer

**Affiliations:** Fondation Mérieux Mali, Immeuble Saibou Sylla, Quartier Hamdallaye ACI 2000, Bamako, Mali

**Keywords:** Laboratoire, Centre de santé communautaire, Populations vulnérables, Zones rurales, Mali

## Introduction

L’état de santé des populations est fortement impacté par l'accès à des soins de santé de qualité. Or c'est la qualité du diagnostic qui va déterminer la conduite thérapeutique. Dans ce contexte, le laboratoire joue un rôle essentiel dans l'amélioration de la qualité des soins, du suivi des malades, ainsi que de la surveillance des maladies chroniques et à potentiel épidémique. C'est aussi un outil clé dans la mise en œuvre des programmes de lutte contre les maladies transmissibles prioritaires (paludisme, VIH, tuberculose). L'installation au Mali de laboratoires de campagne dans les centres de santé communautaire dirigés par des médecins a pour but d'améliorer la prise en charge médicale des populations rurales, surtout des mères/enfants, à travers un diagnostic et un suivi biologique de qualité.

## Méthode

Pour garantir un fonctionnement durable, le processus d'installation d'un laboratoire comprend: la prospection/étude de faisabilité, la définition du paquet d'activités à offrir avec leurs procédures opérationnelles standardisées (SOP) et les équipements nécessaires, la formation du couple médecin-technicien de laboratoire et celle des manœuvres sur la gestion des déchets biomédicaux. De plus, un processus de suivi de formation et d’évaluation externe de la qualité est mis en place.

## Résultats

De 2011 à nos jours, 22 laboratoires ruraux ont été installés dans les régions de Kayes, Koulikoro, Sikasso et Ségou, et sont fonctionnels; et 61 ressources humaines ont été formées (24 médecins, 37 techniciens de laboratoire). Tous les examens biologiques réalisés ont été validés sur la base de SOP. Ces laboratoires ont pu réaliser 119 816 analyses biologiques dont 39 378 chez les enfants de moins de 5 ans. Pendant les consultations prénatales, 20 003 femmes enceintes ont bénéficié de bilans prénataux. 35 686 taux d'hémoglobine chez les enfants et les femmes enceintes ont permis de détecter 17 404 cas d'anémie. Dans le cadre de la recherche du paludisme, en plus des tests de diagnostic rapides (TDR), 42 261 gouttes épaisses ont été réalisées, dont 25 769 positives. Dans le cadre de la lutte contre la drépanocytose, environ 10 000 TDR ont été utilisés et plus de 350 cas de drépanocytose majeure diagnostiqués.

## Conclusion

22 laboratoires de campagne (LABOMEDCAMP) ont été installés et sont fonctionnels en zones rurales de 4 régions du Mali avec l'adhésion du personnel de santé et la participation de la population. L'intégration des activités de ces laboratoires dans le processus de suivi de formation et d’évaluation externe de la qualité ainsi que dans les activités de routine des centres de santé de référence est gage de pérennisation.

# Research article: Point sur le développement clinique d'un candidat-vaccin vivant atténué (1 dose) contre le chikungunya

**Published:** 2023-03-02

**Authors:** Vera Buerger, Martina Schneider, Robert McMahon, Sandra Hadl, Marivic Narciso, Sebastian Toepfer, Ulrike Fuchs, Romana Hochreiter, Annegret Bitzer, Karin Kosulin, Robert Mader, Oliver Zoihsl, Katrin Dubischar, Nina Wressnigg, Susanne Eder-Lingelbach, Juan-Carlos Jaramillo

**Affiliations:** Valneva Austria GmbH, Vienne, Autriche

**Keywords:** Chikungunya, Vaccin, Essai clinique, Vaccin vivant atténué, Immunogénicité

VLA1553 est un candidat vaccin monovalent vivant atténué contre le virus chikungunya (CHIKV). Il a été conçu en supprimant une partie du génome du virus chikungunya.

Un essai clinique de phase 1 randomisé en aveugle a évalué l'innocuité et l'immunogénicité de trois dosages de VLA1553, administrés en intramusculaire chez 120 participants (NCT03382964). Cela a été suivi d'une étude pivot de phase 3, randomisée multicentrique en double aveugle, *versus* placebo (NCT04546724). L'essai VLA1553-301 a recruté 4 115 adultes âgés de 18 ans et plus sur 44 sites aux États-Unis. Un autre essai de phase 3 a évalué la bio-équivalence entre 3 lots de VLA1553 chez 408 adultes en bonne santé randomisés pour chaque lot 1:1:1 (NCT04786444). Les données d'innocuité et d'immunogénicité ont été recueillies pendant 29 jours après la vaccination dans les 2 essais.

Dans l'essai clinique de phase 1, une dose unique de VLA1553 a été bien tolérée et hautement immunogène dans la population adulte. Le développement d'anticorps neutralisants contre le virus chikungunya a été observé avec un taux de séroconversion de 100% chez 120 participants sains. Ce taux a été maintenu jusqu’à 12 mois. Sur la base de ces résultats, VLA1553 est entré en développement clinique de phase 3.

Le premier essai pivot a atteint son critère d’évaluation principal avec une séroprotection de 98,9% (263 des 266 participants, IC95%: 96,7-99,8) un mois après vaccination avec une dose unique de VLA1553. La séroprotection était maintenue à 96,3% six mois après l'administration d'une dose unique. L'innocuité et la tolérance sont donc validées.

L’étude de bio-équivalence n'a montré aucune différence significative entre les lots en ce qui concerne les titres d'anticorps. VLA1553 était également hautement immunogène puisque la séroprotection a été obtenue chez 97,7% des participants. VLA1553 a également été bien toléré avec un profil de tolérance favorable dans les essais de phase 3.

La génération de titres protecteurs chez près de 100% des participants vaccinés analysés indique que le VLA1553 est un candidat efficace pour la prévention des maladies causées par le CHIKV.

# Research article: Spécificités des usagers d'une consultation hospitalière de médecine tropicale en Amazonie française

**Published:** 2023-03-02

**Authors:** Marie Poirot-Fouillet

**Affiliations:** Unité des maladies infectieuses et tropicales, CH de Cayenne, Guyane, France

**Keywords:** Guyane, Précarité, Amérique latine, Médecine tropicale, Soins infirmiers

## Introduction

Les soins prodigués en zone tropicale sont complexes du fait des spécificités épidémiologiques, climatiques, culturelles et sociales. Les soignants exerçant en zone tropicale doivent ainsi faire preuve de capacités d'adaptation permanentes. L'objectif de ce travail était de décrire les particularités du travail d'une infirmière et d'une secrétaire d'un service de consultation hospitalière en milieu amazonien à travers la description de la population consultante.

## Méthodologie

Entre le 30 septembre 2021 et le 23 mars 2022, après recueil d'un consentement écrit, ont été inclus des patients se présentant à la consultation de maladies infectieuses et tropicales du Centre hospitalier de Cayenne. Un recueil de données administratives, sociodémographiques et cliniques a été réalisé par l'infirmière et la secrétaire de la consultation. Ont été exclus les patients adressés pour prise en charge du risque rabique et pour vaccination internationale, et ceux ayant refusé de participer à l’étude.

## Résultats

Cent patients ont été inclus, parmi lesquels 42% de femmes, et 71% étaient en avance à leur rendez-vous. Parmi eux, 56% consultaient pour le suivi d'une infection virale chronique (VIH N = 38; VHB N = 10; HTLV1 N = 3; VHC N = 2) et les autres pour le suivi d'une infection aiguë (dont infection bactérienne cosmopolite (13), fièvre Q (9), Covid-19 (13)). Les pays de naissance les plus représentés étaient la France (36%, dont la Guyane 26%), Haïti (34%) et le Brésil (13%). Parmi les patients nés hors de France, 41% étaient sans-papiers et près d'un sur deux était en situation de précarité comme en témoigne leur couverture maladie (aucune (7), Aide médicale de l’État (AME) (10), Complémentaire santé solidaire (CSS) (32)). Les langues maternelles étaient par ordre de fréquence le créole haïtien (34), le français (31), le portugais (13), suivi du créole guyanais (7); 66% savaient lire et écrire en français, 76% dans leur langue maternelle et 11% rapportaient ne pas du tout parler français. Le niveau scolaire était variable avec 20 qui n’étaient pas allés à l’école au niveau primaire, 29 au collège, 26 au lycée et 26 avaient fait des études supérieures; 36% n'avaient pas de travail ou faisaient des petits jobs et 27% vivaient en squat, location non déclarée ou étaient hébergés chez un tiers. Enfin, 10% n'avaient pas accès à l'eau courante et 8% à l’électricité. Seuls 44% possédaient une voiture personnelle et 15% un deux-roues. À l'opposé, 99% disposaient d'un téléphone, 88% parlaient au moins 2 langues (34% ≥ 3) et 55% disposaient d'un courriel. Enfin, il est notable que 64% avaient recours à la pharmacopée traditionnelle.

## Discussion

Plus que la spécificité des pathologies, ce qui ressort de cette enquête est une grande précarité de la population prise en soins avec un niveau socio-éducatif faible entraînant des difficultés supplémentaires spécifiques pour les paramédicaux exerçant en zone tropicale. Ces vulnérabilités sont cependant à mettre en perspective de grandes capacités d'adaptation et de la mobilisation de ressources personnelles, de la part des patients comme des soignants.

# Research article: Facteurs associés à la Covid-19 chez les enfants de 0 à 15 ans au Niger en 2020

**Published:** 2023-03-02

**Authors:** Habibatou Idé, Pauline Yanogo, Dieudonné Tialla, Djibril Barry, Éric Adehossi, Nicolas Meda

**Affiliations:** Burkina Field Epidemiology and Laboratory Training Program (BFELTP), Université Joseph Ki-Zerbo, Ouagadougou, Burkina Faso

**Keywords:** Facteurs, Covid-19, Enfants, Base de données, Niger

## Contexte

Le 30 janvier 2020, l'OMS a déclaré comme urgence sanitaire mondiale la Covid-19. Les enfants étaient atteints avec des formes moins sévères. Face à cette pandémie, le Niger a mis en place des mesures dans un contexte où les enfants constituent une source de contamination. L'objectif de cette étude est de déterminer les facteurs associés à la Covid-19 chez les enfants au Niger de février à août 2020 à travers une analyse de la base de données nationales.

## Méthodes

Nous avons mené une étude transversale analytique incluant tous les cas négatifs et positifs à la Covid-19 de la base de données nationale durant 6 mois. Nous avons utilisé les logiciels Excel et Epi Info 7.2.4 pour l'extraction et l'analyse des données. Les fréquences et proportions ont été calculées, nous avons dans une régression logistique estimé les OR d'association avec leur intervalle de confiance à 95%, les facteurs associés à la Covid-19 au seuil de p < 0,05.

## Résultats

Nous avons trouvé 572 cas suspects de Covid-19 chez les enfants de 0-15 ans notifiés de février à août 2020 dont 11,36% étaient positifs au test RT-PCR. L’âge médian des enfants infectés était de 10 ans [EIQ: 5-13 ans]. Le sex-ratio Homme/Femme était de 2,1. Le groupe d’âge de 11 à 15 ans était le plus représenté (49,2%). Toujours parmi les cas positifs, 61,5% résidaient à Niamey, 4,6% avaient des comorbidités, 12,3% avaient une notion de voyage 14 jours avant le début des signes et 40% avaient une notion de contact avec un cas probable ou confirmé. 56,9% présentaient des symptômes dont principalement la fièvre (24,4%) et la toux (23,2%) avec 18% d'hospitalisés. Un décès a été enregistré soit une létalité à 1,5%. En analyse étiologique, les facteurs associés à la Covid-19 étaient le sexe ORa = 0,51 [0,28-0,93] p = 0,028, la présence de symptômes ORa = 2,29 [1,23-4,25] p = 0,008 et le contact avec un cas probable ou confirmé ORa = 0,32 [0,13-0,77] p = 0,011.

## Conclusion

Les enfants exposés sont généralement sensibles à la Covid-19 et toutes les tranches d’âges sont atteintes avec une prédominance masculine. Nous recommandons des mesures barrières adaptés aux plus jeunes, une précocité dans la détection et la prise en charge des enfants infectés afin de protéger les personnes vulnérables, telles que les personnes âgées et les personnes présentant des comorbidités.

# Research article: Premiers gestes et itinéraire thérapeutique des enfants victimes de sténose caustique de l’œsophage à l'hôpital du Mali

**Published:** 2023-03-02

**Authors:** Mamadou Cisse, Issa Boubacar Maiga, Abdoul Aziz Maiga, Allaye Ombotimbe, Moussa Bazongo, Mamadou Salo Koita, Soungalo Diop, Fatoumata Konaté, Ibrahim Coulibaly, Adama Issa Koné, Sanibé Dramane Koné, Souleymane Coulibaly, Seydou Togo, Moussa Abdoulaye Ouattara, Sadio Yena

**Affiliations:** Service de chirurgie thoracique, CHU Hôpital du Mali, Faladié, Bamako, Mali

**Keywords:** Œsophage, Sténose caustique, Premiers soins, Parcours

## Introduction

Les sténoses caustiques de l’œsophage chez l'enfant (SCOE) arrivent tardivement dans le service pour une prise en charge (PEC).

## Objectif

Déterminer les premiers gestes après l'ingestion caustique et le parcours de soins des victimes de SCOE prises en charge (PEC) à l'hôpital du Mali.

## Matériel et méthodes

Il s'agissait d'une étude rétrospective (janvier 2012 à décembre 2017) et prospective (janvier à décembre 2018) décrivant les soins prodigués devant une ingestion de produit caustique ainsi que l'itinéraire des cas de SCOE recensés dans le service de chirurgie thoracique à l'hôpital du Mali. Les premiers gestes de sauvetage, les relais de soins et le délai d'arrivé dans le service ont été revus.

## Résultats

Au total, 70 cas de SCOE ont été recensés avec une moyenne d’âge de 5,8 ans (0-15 ans) et un sex-ratio de 1,41. L'ingestion était accidentelle dans 95,7%. Après l'ingestion 95,7% des victimes ont eu une PEC immédiate sur le lieu de l'accident. Le premier geste était de faire boire un antidote traditionnel (lait dans 65,7%, eau dans 8,6%, faire vomir l'enfant 8,6%, etc.). Il y avait une référence secondaire dans un centre de santé moderne dans 98,6% des cas. Avant une prise en charge dans le service, les victimes sont passées par 2 centres de soins (55,7%), 3 centres (24,3%) et 4 centres (11,4%). Dans 100% des cas, aucune modalité de PEC n'est décrite dans ces centres. Nous avions enregistré 43 (61,4%) cas de guérison, 12 (17,1%) cas de décès et 15 (21,5%) étaient en cours de traitement.

## Conclusion

L'ingestion caustique chez un enfant constitue une extrême urgence médicochirurgicale. Dans notre communauté, les premiers gestes en cas d'ingestion de produits caustiques sont largement dominés par les attitudes traditionnelles ancestrales qui aggravent et retardent leur PEC adéquate. Les victimes fréquentent plusieurs structures de soins avant d'arriver dans le service. L’élaboration et la diffusion large d'un protocole de références et de prévention doivent être faites pour améliorer cette situation.

# Research article: Étude des déterminants de la couverture vaccinale complète chez les enfants âgés de 12 à 23 mois dans le district sanitaire de Podor (Sénégal)

**Published:** 2023-03-02

**Authors:** Oumar Bassoum, Coumba Ndiaye, Ndeye Mareme Sougou, Mamadou Makhtar Mbacké Leye, Adama Faye, Ibrahima Seck

**Affiliations:** Faculté de médecine, de pharmacie et d'odontologie, Université Cheikh Anta Diop, Dakar, Sénégal

**Keywords:** Couverture vaccinale, Enfants, Sénégal

## Introduction

Le Sénégal a mis en place le programme élargi de vaccination (PEV) depuis 1979 afin d'améliorer la couverture vaccinale chez les enfants. La proportion d'enfants complètement vaccinés à la fin de leur première année de vie représente un indicateur de performance du PEV. Or, selon les récentes données, cet indicateur n'est pas optimal. L'objectif de cette étude était d'identifier les déterminants de la couverture vaccinale complète chez les enfants âgés de 12 à 23 mois dans le district sanitaire de Podor.

## Méthodologie

L’étude était transversale et conduite du 19 au 22 juin 2021 dans le district sanitaire de Podor situé au Nord du Sénégal, à 405 km de Dakar. La taille de l’échantillon a été estimée à 870 enfants âgés de 12 à 23 mois. Un sondage à deux degrés était réalisé. Les données étaient collectées au cours d'un entretien individuel dans les ménages. Un questionnaire électronique était élaboré à l'aide de l'application Open Data Kit téléchargée sur smartphone. Les sources de données vaccinales sont la déclaration de la mère ou gardienne d'enfant, la fiche de santé conservée à domicile (FCD) et le registre de l’établissement de santé fréquenté par l'enfant. Les déterminants de la couverture vaccinale complète étaient identifiés à l'aide de la régression logistique. L'odds ratio ajusté (ORa) et son intervalle de confiance à 95% (IC95%) étaient calculés.

## Résultats

Au total, 832 enfants étaient inclus. Parmi eux, 53% sont de sexe masculin, 45,9% sont nés de mères non scolarisées, 72,8% résident en zone rurale et 68,8% sont nés dans les établissements de santé. Concernant le statut vaccinal, 88,9% des enfants ont été complètement vaccinés. Les facteurs positivement associés à la couverture vaccinale complète sont la satisfaction des mères ou gardiennes d'enfants vis-à-vis des services de vaccination (ORa = 2; IC95%: 1,07-3,72), la disponibilité d'une FCD (ORa = 4,78; IC95%: 1,73-13,2), quatre consultations prénatales au moins (ORa = 1,81; IC95%: 1,07-3,04) et la pesée du nouveau-né immédiatement après la naissance (ORa = 4,08; IC95%: 2,19-7,6).

## Conclusion

Cette étude montre qu'il serait nécessaire de mettre en place des stratégies pour accroître la couverture vaccinale complète. Il faudrait veiller à la satisfaction des usagers des services de vaccination en améliorant la qualité des prestations, promouvoir la vaccination infantile durant les consultations prénatales et les soins du nouveau-né comme la pesée, distribuer les FCD, y notifier l'information vaccinale et sensibiliser sur l'importance de leur bonne conservation.

# Research article: Évaluation de la résistance du VIH aux antirétroviraux chez les patients en suppression virale au Cameroun

**Published:** 2023-03-02

**Authors:** Rachel Audrey Nayang Mundo, Collins Chenwi, Alex Durand NKA, Ezechiel Ngoufack Jagni Semengue, Aude Christelle Ka'e, Willy Pabo, Désiré Takou, Aissatou Abba, Grace Beloumou, Sandrine Djupsa, Vittorio Colizzi, Carlo Federico Perno, Alexis Ndjolo, Joseph Fokam

**Affiliations:** Centre international de référence Chantal Biya pour la recherche sur la prévention et la prise en charge du VIH/SIDA (CIRCB), 3077 Yaoundé-Messa, Cameroun

**Keywords:** VIH, Suppression virale, Génotype, Résistance aux antirétroviraux, Cameroun

## Objectifs

La suppression virale (charge virale < 1000 copies/ml) est considérée comme un succès thérapeutique dans les pays à ressources limitées. Cependant, plusieurs études ont montré la présence de mutations de résistance à des charges virales résiduelles, ce qui pourrait compromettre la réponse thérapeutique au long terme des patients et compromettre leur pronostic vital. Notre objectif était d’évaluer l'effectivité du séquençage et de déterminer le profil génotypique de résistance du VIH aux antirétroviraux chez les patients en suppression virale au Cameroun.

## Méthodes

Une étude transversale et analytique a été menée au Centre international de référence Chantal Biya (CIRCB) de janvier 2020 à août 2021 chez les patients en suppression virale. Les données sociodémographiques, cliniques et immuno-virologiques ont été recueillies à partir du dossier médical. Le séquençage a été réalisé dans la région protéase-transcriptase inverse du VIH-1, le profil génotypique analysé suivant l'algorithme de Stanford HIVDB version 9.0, et la phylogénie moléculaire réalisée avec MEGAX. Le taux de séquençage et le seuil des mutations de résistance ont été évalués selon la virémie, avec toute valeur p < 0,05 considérée comme statistiquement significative.

## Résultats

Sur un total de 132 participants (âge médian [IQR] 43 [33-51] ans, 69% de femmes), la durée médiane sous traitement antirétroviral (TARV) était de 19 (IQR: 12-34,4) mois. Le taux d'amplification était de 39 (IC95%, 21,93-38,11%), et le taux de réussite du séquençage était de 28,8% (38/132), soit 97,4% des amplicons. Le génotypage était plus effectif pour les patients avec virémies ≥ 200 copies/ml, soit 47,2% (25/53) contre 16,5% (13/79) pour les virémies ≤ 200 copies/ml, p < 0,001. Sur les 38 séquences générées, le taux global de résistance était de 89,7%, avec selon les classes médicamenteuses 79,9% pour les INTI; 79,4% pour les INNTI et 15,3% pour les IP/r. Ce taux de résistance était plus élevé chez les patients avec charge virale ≥ 200 copies/ml, soit 32% contre 7,7% pour les patients à charge virale ≤ 200 copies/ml (OR = 5,65; p = 0,13). Sept sous-types viraux ont été identifiés avec une prédominance de la forme recombinante CRF02_AG (64%). Les mutations M184V (74,3%) et K103N (45,7%) étaient les plus fréquentes dans la transcriptase inverse et M46I (14,2%) dans la protéase. Le profil de susceptibilité virale révélait 41,1% (14/38) des participants sous combinaisons thérapeutiques sous-optimales.

## Conclusion

Dans un contexte de forte diversité génétique du VIH comme le Cameroun, le séquençage paraît effectif chez la moitié des patients en suppression virale avec une virémie d'au moins 200 copies/ml. De plus, l’émergence de mutations majeures de résistance chez ces patients s'avèrerait plus considérable avec une charge virale ≥ 200 copies/ml. Chez ces patients en suppression virale, prés de 4 sur 10 auraient besoin d'une optimisation de la thérapie, pour une réussite thérapeutique au long terme.

# Research article: Plateforme numérique pour soutenir la gestion et la sécurité des données des tests diagnostiques de la Covid-19 dans le contexte de ressources limitées: le cas de PlaCARD au Cameroun

**Published:** 2023-03-02

**Authors:** Jules Brice Tchatchueng-Mbougua, Loique Landry Messanga Essengue, Francis Jaudel Septoh Yuya, Vanessa Kamtchogom, Achta Hamadou, Serge Alain Sadeuh-Mbah, Paul Alain Tagnouokam Ngoupo, Maurice Tchuente, Richard Njouom, Sara Eyangoh, Mathurin Cyrille Tejiokem

**Affiliations:** Service d’épidémiologie et de santé publique, Centre Pasteur du Cameroun, Réseau international des Instituts Pasteur (RIIP)

**Keywords:** Système d'information de laboratoire, Gestion de données, Riposte, Covid-19

Au cours de la pandémie de Covid-19, la demande de tests moléculaires de Covid-19 a dépassé les capacités des laboratoires, ce qui a imposé une charge importante au personnel et aux infrastructures des laboratoires. Il est devenu impératif d'exploiter la puissance des systèmes d'information de laboratoire (SIL) pour rationaliser toutes les phases des tests de laboratoire (pré-analytique, analytique et post-analytique). L'objectif de cette étude était de décrire l'architecture, la mise en œuvre et les exigences de PlaCARD (Plateforme de collecte, d'analyse, de visualisation et de rapport de données), une plateforme logicielle permettant de gérer l'enregistrement des patients, les échantillons et le flux de données du diagnostic moléculaire de la Covid-19, ainsi que le rendu et l'authentification des résultats de diagnostic pendant la pandémie de coronavirus de 2019 (Covid-19) au Cameroun.

En s'appuyant sur son expérience en matière de biosurveillance, le Centre Pasteur du Cameroun (CPC) a développé une plateforme numérique en temps réel et open source comprenant des applications web et mobiles, appelée PlaCARD, afin d'améliorer l'efficacité et le temps des interventions de contrôle de la maladie. PlaCARD a été rapidement adapté à la stratégie de décentralisation des tests de la Covid-19 au Cameroun et déployé dans tous les laboratoires de diagnostic de la Covid-19 et dans le centre régional des opérations d'urgence après une formation spécifique des utilisateurs.

Globalement, 71% des échantillons analysés pour le diagnostic moléculaire de la Covid-19 au Cameroun du 5 mars 2020 au 31 octobre 2021 ont été saisis dans PlaCARD. Le délai médian de rendu des résultats était de 2 jours [0-2,3] avant avril 2021 et a diminué à 1 jour [1-1] après l'introduction de la notification des résultats par SMS dans PlaCARD.

L'intégration d'un SIL et de la gestion du flux de travail dans une seule plateforme logicielle complète a renforcé les capacités de la riposte à la Covid-19 au Cameroun. PlaCARD a montré sa capacité à être utilisé comme SIL pour gérer et sécuriser les données des tests pendant la riposte à une épidémie.

# Research article: LabBook 3.0, un SIL open source développé pour informatiser les laboratoires de biologie médicale et améliorer la surveillance épidémiologique

**Published:** 2023-03-02

**Authors:** Anja Mampianina Ramilson, Mafoudji Kande, Oumar Kanté, Alexandre Charles, Philippe Meurier, Philippe Brun, Aïcha Marceline Sarr, Laurent Raskine, François-Xavier Babin, Nicolas Steenkeste

**Affiliations:** Fondation Mérieux, Lyon, France

**Keywords:** Biologie médicale, Surveillance, SIL, Open source

La Fondation Mérieux s'engage à lutter, sur le terrain, contre les maladies infectieuses qui affectent les pays en développement en renforçant les capacités, notamment des laboratoires de biologie médicale afin d'améliorer l'accès au diagnostic pour une meilleure prise en charge des patients.

L'un des objectifs est de développer un système d'information de laboratoire (SIL) pour aider et guider les laboratoires passant des cahiers de laboratoire papier à une solution logicielle facile à utiliser et à maintenir. Un SIL gère l'activité du laboratoire depuis la demande d'analyse jusqu'au rendu de résultat, mais aussi la traçabilité des échantillons, des utilisateurs, des équipements, les stocks et approvisionnements.

C'est ainsi que LabBook a été créé en 2010, cofinancé par l'Agence française de développement (AFD) et la Fondation Mérieux. Le logiciel est disponible sur le site LabBook: www.lab-book.org/.

LabBook 3 a été lancé en août 2021, avec la version 3.0.13 développée par la société AEGLE. LabBook a été désigné pour répondre aux besoins des utilisateurs avec une interface intuitive et suivre le flux de travail naturel du laboratoire. Il dispose d'un module de management de la qualité pour partager les procédures ou encore suivre notamment la maintenance des équipements. LabBook comprend également un module de gestion des stocks.

Pour renforcer la capacité de surveillance épidémiologique, LabBook est désormais capable d'exporter vers deux logiciels, DHIS2 et WHONET, qui permettent respectivement d'informatiser la remontée de données aux autorités de santé, d'augmenter les capacités d'analyse, mais aussi d'analyser la résistance aux antimicrobiens en faisant le lien avec le système de surveillance de l'OMS, GLASS. LabBook est librement téléchargeable à partir de son site internet et il est déployé dans le cadre des projets de la Fondation Mérieux en partenariat avec les ministères de la Santé, notamment RESAOLAB, Réseau d'Afrique de l'Ouest des laboratoires au Bénin, Burkina Faso, Guinée, Mali, Niger, Sénégal, Togo (www.fondation-merieux.org/projets/resaolab-fr); mais également avec REDISSE (www.fondation-merieux.org/projets/redisse-fr) en Mauritanie et au Nigéria; et enfin avec RESAMAD, le réseau des laboratoires à Madagascar.

Le logiciel a déjà pu avoir 2 évolutions avec les versions 3.1 et 3.2 qui ont permis l'ajout d'un grand nombre de fonctionnalités.

LabBook 3 est basé sur une nouvelle infrastructure qui lui permet d’être plus léger et rapide utilisant MySQL, Python et Podman. En tant que solution open source, le code est disponible sur Github: https://github.com/fondationmerieux/labbook_python.

# Research article: L'outil de surveillance épidémiologique dans les armées face à la crise sanitaire liée à la Covid-19

**Published:** 2023-03-02

**Authors:** Jean-Marie Loreau, Franck De Laval

**Affiliations:** Centre d’épidémiologie et de santé publique des armées, Marseille, France

**Keywords:** Surveillance épidémiologique, OSEA, Covid-19, Santé publique, Forces armées

## Introduction

La surveillance épidémiologique dans les armées porte sur 61 évènements de santé: maladies à déclaration obligatoire communes au milieu militaire et civil et d'autres pathologies d'intérêt spécifique au milieu militaire. Le service de surveillance épidémiologique et investigation (SEI) basé à Marseille au sein du centre d’épidémiologie et de santé publique des armées centralise les déclarations de ces évènements provenant des 320 antennes médicales militaires soutenant les 300 000 militaires français postés en métropole, en outre-mer et à l’étranger. Les objectifs sont de détecter précocement les évènements sanitaires, émergences ou épidémies qui nécessitent une réaction urgente, et de fournir des données épidémiologiques utiles au pilotage des crises sanitaires ou des plans de santé publique et à l'identification des priorités de santé et de recherche.

## Méthodes

Depuis 2018, le SEI a développé et déployé un outil de surveillance informatisé, OSEA (outil de surveillance épidémiologique dans les armées), permettant une déclaration en ligne et en direct par le personnel soignant qu'il soit en métropole, outre-mer ou à l’étranger. Cet outil, collaboratif entre les déclarants, les analystes et les décideurs, permet entre autres une meilleure réactivité dans l'analyse des signaux, une sécurisation et une exploitation optimisée des données. Il a été un support indispensable dès le début de la crise sanitaire liée à la Covid-19.

## Résultats

OSEA héberge actuellement plus de 245 000 évènements de santé déclarés depuis 2003 et plus de 72 200 fiches de déclaration depuis 2012; 3000 fiches étaient déclarées annuellement. Lors de la crise sanitaire liée à la Covid-19 plus de 30 000 fiches spécifiques ont été saisies sur OSEA chaque année dont les fiches Covid-19 et évènements indésirables des vaccins. Les données étaient immédiatement disponibles pour identifier les signaux sanitaires urgents, et pour établir des indicateurs utiles au pilotage de cette crise.

## Discussion

OSEA a permis une simplification du processus de surveillance avec des déclarations au fil de l'eau et une surveillance en quasi-temps réel. Lors de la crise sanitaire, il a permis d'absorber l'augmentation d'activité de 750% et d’éditer des bulletins de rétro-information quotidiens utiles à la conduite de crise. Ce système doit néanmoins évoluer vers une meilleure interopérabilité avec le dossier médical patient et une meilleure performance en termes de disponibilité en contexte opérationnel.

# Research article: Couplage des systèmes d'information géographiques et des réseaux de Pétri dans le suivi des malades au Gabon: étude à partir de la dynamique de propagation de la Covid-19

**Published:** 2023-03-02

**Authors:** Marius Massala, Pierre Moukeli Mbindzoukou, Euloge Makita-Ikouaya

**Affiliations:** Libreville, Gabon

**Keywords:** Covid-19, Épidémie, Gabon, Réseau de Pétri, Système d'information

L’étude de la propagation de la Covid-19 a fait l'objet de plusieurs approches mathématiques. Mais ces démarches de modélisation se sont heurtées à l'instabilité des données collectées, notamment celles concernant le nombre des individus infectés. Au Gabon, de nombreuses personnes ont contracté le virus sans présenter de symptômes alors qu'elles pouvaient le transmettre. Pour comprendre la dynamique spatio-temporelle de cette maladie, nous avons proposé une approche couplant à la fois les Systèmes d'information géographique et les réseaux de Pétri. Il s'agit ici de suivre l’état clinique d'un malade, de prédire son évolution en tenant compte des différents délais de transmission du virus. Pour construire notre démarche, nous nous sommes appuyés sur les données cliniques collectées auprès du Centre hospitalo-universitaire de Libreville (CHUL). La méthodologie adoptée est la suivante: nous avons considéré que la population gabonaise non infectée par la Covid-19 se divise en deux catégories: les personnes normales et les personnes vulnérables. Un Homme (H) peut s'infecter au contact d'une personne infectieuse. La personne ainsi infectée (I), après deux à quatre jours d'incubation, devient infectieuse. Un infectieux peut être asymptomatique (A), bénin (B), modéré (M) ou encore sévère (S). Un individu asymptomatique peut soit guérir, soit devenir bénin; de ce dernier état, il peut aussi soit guérir ou devenir modéré. Lorsqu'un infectieux est modéré ou sévère, il est systématiquement hospitalisé (H) et dans ce cas, son état peut s'améliorer et il va guérir (G) ou s'aggraver pour le conduire malheureusement au décès (D). La personne guérie acquiert une immunité qu'elle peut perdre après un certain temps. À partir des différents éléments que nous venons de décrire, nous apprécions le passé d'un individu d'un état à un autre, comme un processus discret. C'est pourquoi, nous avons utilisé le réseau de Pétri pour représenter le système sous forme d'un automate d’état fini. Dans cet automate, H est l’état initial, G et D sont des états finaux. Le passage d'un état à un autre est réglé par une fonction mathématique de transition. Les résultats obtenus par cette approche ont été prometteurs car la marge d'erreur est très marginale. Sur la base des résultats de cet automate fini, nous avons construit un Système d'information géographique décrivant la situation spatio-temporelle des malades au Gabon durant cette période.

# Research article: Surveillance épidémiologique en temps réel de l'infection par le SARS-CoV-2 dans la région sanitaire Sud-Est (Haut-Ogooué) au Gabon, Afrique Centrale

**Published:** 2023-03-02

**Authors:** Pater Noster Sir-Ondo-Enguier

**Affiliations:** Unité Évolution, épidémiologie et résistances parasitaires (UNEEREP), Centre interdisciplinaire de recherches médicales de Franceville (CIRMF), Franceville, Gabon

**Keywords:** Surveillance, Maladies infectieuses, Covid-19, Détection, Gabon

## Objectifs

Décrire les caractéristiques en temps quasi réel des cas positifs de SARS-CoV-2 dans la région sanitaire Sud-Est dans le contexte de la pandémie de Covid-19, puis décrire les premiers résultats de cette surveillance.

## Patients et méthodes

Étude prospective observationnelle multicentrique incluant tous les cas confirmés de Covid-19 chez les patients résidents ayant consulté dans un hôpital de la région. Pour chaque patient nous avons recueilli l'histoire de la maladie, les données démographiques et les comorbidités.

## Résultats

Sur ces 2 880 patients, 932 (32,4%) avaient été notifiés à Lébombi-Léyou (dont 897 à Moanda), 1 874 (65%) avaient été notifiés à Passa (Franceville), 33 (1,14%) dans le département de Lékoko (Bakoumba), 12 (0,42%) à Sébé-Brikolo (Okondja), 10 (0,3%) à Lékabi-Léwolo (Ngouoni), 8 (0,28%) à Djouori-Agnili (Bongoville), 5 (0,2%) dans le département de Lékoni-Lékori (Akiéni), 4 (0,1%) dans l'Ogooué-Létili (Boumango) et 2 (0,07%) dans les Plateaux (Léconi). Sur les 60 cas ayant au moins une comorbidité, 16 (26,6%) sont décédés dans la région sanitaire du Sud-Est. Les dossiers cliniques de ces cas avaient été analysés, seul 1/16 patient décédé (6,2%) n'avait aucune pathologie associée.

## Conclusion

La détection précoce de la Covid-19 a été essentielle dans le Haut-Ogooué pour isoler les cas confirmés et prévenir une nouvelle transmission. Parmi les cas de Covid-19 confirmés en laboratoire, les patients présentant une comorbidité avaient de moins bons résultats cliniques que ceux qui n'en avaient aucune. Un plus grand nombre de comorbidités était également corrélé à de moins bons résultats cliniques.

# Research article: Recours aux outils de localisation spatiale dans la gestion des épidémies: exemple du Service de santé militaire du Gabon

**Published:** 2023-03-02

**Authors:** Laurette Guignali Mangouka, Berthe Amélie Iroungou, Ghislain Moussavou, Michel Ngui Ondo, Armel Nzue Mba, Jean Raymond N'zenze

**Affiliations:** Service de santé militaire du Gabon, Hôpital d'instruction des armées Omar Bongo Ondimba, Libreville, Gabon

**Keywords:** Épidémies, Service de santé militaire, Gabon, AGEOS, Télé-épidémiologie

Les technologies et outils de l'Agence d'observation et de surveillance spatiale (AGEOS) en télé-épidémiologie ont été d'un apport significatif durant la pandémie à Covid-19 au Gabon.

Dans cette optique, le Service de santé militaire du Gabon (SSM), fort de son partenariat avec l'AGEOS, a mis à contribution sa plateforme de gestion des épidémies.

Les objectifs de ce partenariat sont: d'une part, l'appropriation des technologies et des outils d'informations spatiales par les personnels du SSM du Gabon et d'autre part, la mise au point d'un démonstrateur de gestion des crises humanitaires.

Un démonstrateur à deux volets a été indiqué dans le contexte d’épidémie (ou de pandémie): l'un pour répondre à la gestion des accidents de masse et l'autre pour apporter une réponse à l'assistance humanitaire en cas de désastre.

La base de données du démonstrateur comprend les données relatives au désastre ou accident de masse, l'accessibilité au site sanitaire et aux voies d'accès ainsi que l'accessibilité aux ressources humaines et logistiques.

Les résultats préliminaires sont: l'intégration des agents du SSM du Gabon dans un processus d'appropriation des technologies et outils d'informations spatiales, et la mise en route du démonstrateur pour la gestion des crises humanitaires.

La mise en place d'une plateforme de gestion des épidémies utilisant des données spatiales au Gabon, représente actuellement un apport considérable dans la stratégie de riposte contre les épidémies et particulièrement en période de pandémie à Covid-19.

De plus, la réalisation des cartes de situation en temps réel durant la pandémie a permis d'optimiser la surveillance et la riposte face à cette maladie émergente au sein de notre pays.

# Research article: Systèmes d'information-alerte et contribution des comités sectoriels de prévention, de surveillance à base communautaire et de soutien social à la riposte contre la Covid-19

**Published:** 2023-03-02

**Authors:** Solange Amethier, Baba Sangaré, Niangoran Mariette Alida Christine Bakou

**Affiliations:** Comité sectoriel de lutte contre le sida du ministère de la Femme, de la famille et de l'enfant, Abidjan, Côte d'Ivoire

**Keywords:** Covid-19, Cas suspect, Alerte, Comité sectoriel

Le ministère de la Femme, de la famille et de l'enfant a mis en place des comités sectoriels de prévention, de surveillance à base communautaire et de soutien social dans le cadre de la riposte à la pandémie du Coronavirus Covid-19 en collaboration avec le Centre des opérations de santé publique (COUSP). Cette collaboration multisectorielle a permis d'assurer une bonne adhésion des populations aux implications sanitaires liées aux mesures sociales de santé publique à travers la mise en place d'un système d'information. Quelle a donc été la contribution des comités sectoriels de prévention, de surveillance à base communautaire et de soutien social installés?

Nous avons mené une enquête transversale descriptive de juin à décembre 2021 portant sur les activités des comités sectoriels installés. Ces activités ont été coordonnées par le pool chargé de la coordination et de l'appui technique des équipes de suivi des contacts et de gestion des cas.

Avant l'installation des comités sectoriels, le système d'alerte mis en place dans les localités était basé sur un mécanisme interservices centralisé au niveau de la préfecture qui s'appuyait principalement sur les structures de santé. Le circuit de transmission des informations partait des agents de santé communautaire vers les centres de santé, puis vers les directions départementales de la santé.

Après la mise en place des comités sectoriels, un système communautaire où les individus peuvent signaler/rapporter les cas (cas suspects ou alertes) a été instauré. Dans ce système, chacun se surveille et surveille l'autre connaissant les symptômes de la maladie. Les activités menées portaient essentiellement sur les sensibilisations de masse et de proximité, le respect des mesures barrières, la démonstration du lavage des mains et du port du masque de protection, le traitement des idées reçues et la promotion de la vaccination contre la Covid-19.Elles ont permis aux membres des communautés de se familiariser avec les symptômes de la maladie, de respecter les mesures barrières, de manifester une adhésion à la vaccination contre la Covid-19 et de se rendre plus prompts au référencement en cas de manifestation à travers des systèmes d'information partant de l'individu, la communauté, les associations/groupements communautaires de soutien, les membres des comités sectoriels, les ONG et les centres sociaux vers les structures sanitaires et les directions départementales de la santé.

Le choix des sites d'intervention s'est fait en fonction du risque accru de contracter la maladie à Covid-19 et de la réticence à l’égard de la vaccination.

De juin à décembre 2021, 47 comités ont été installés et ont mené 3 962 activités de plaidoyer et de mobilisation communautaire,15 881 sensibilisations de masse, 5 355 focus groupes, 5 001 visites à domicile sécurisées, 8 699 entretiens individuels, 327 810 personnes touchées, 22 cas alertes référés, 7 783 idées reçues traitées, 807 sorties communautaires dans les publics (gare, école, marché, lieux de culte), 85 631 personnes sensibilisées sur le test de dépistage et la vaccination contre la Covid-19. L'installation des comités sectoriels a permis d'améliorer les systèmes d'information-alerte existants afin de prévenir, de détecter, de référer et d'accompagner au besoin des cas suspects ou alertes dans un contexte de crise sanitaire liée à la Covid-19.

# Research article: Évaluation du fonctionnement des Équipes d'intervention rapide (EIR) et des Équipes de suivi des contacts (ESC) dans la riposte contre la Covid-19 en Côte d'Ivoire

**Published:** 2023-03-02

**Authors:** Harvey Attoh Touré, Kounangui Marie Noëlle Ano Ama, Konan Roland Oussou, Bangama Christian Akani, Konan N'guessan, Kouakou Éric Ahoussou, Joseph Bénié Bi Vroh, Issaka Tiembré

**Affiliations:** Institut national d'hygiène publique (INHP), Abidjan, Côte d'Ivoire

**Keywords:** Covid-19, Riposte, Côte d'Ivoire

## Introduction

Le 11 mars 2020, la Côte d'Ivoire a déclaré son premier cas de Covid-19. La surveillance épidémiologique avait pour objectif de détecter rapidement tous les cas, et cela grâce aux EIR et aux ESC.

## Objectif

Évaluer le fonctionnement des Équipes d'intervention rapide (EIR) et des Équipes de suivi de contacts (ESC) dans la riposte contre la Covid-19 en Côte d'Ivoire.

## Méthodes

Il s'agit d'une étude évaluative transversale, portant sur tous les membres des EIR, ESC et leurs superviseurs au niveau de l'INHP, de la DSASA et des districts des 2 régions sanitaires d'Abidjan 1 et 2 ainsi que des DDS enquêtés. La collecte des données s'est faite à l'aide d'un questionnaire digitalisé et d'une revue documentaire. La saisie et l'analyse des données ont été faites à l'aide du logiciel Epi Info version 3.5.3.

## Résultats

Au total, 149 EIR et 229 ESC ont été interrogées. Âge moyen 38 ans dont la moitié avec une expérience moyenne de 153 jours, environ 4 mois par rapport à la date d'enquête. Les enquêtés ont reçu pour la majorité (> 95%) des formations de base. La majorité (81%) des Districts sanitaires, INHP (16%) et DSASA (3%). Sur 104 alertes recensées dans notre étude, le délai maximal écoulé entre l'alerte et l'investigation était de 11,45 h et le délai minimal de 10,80 h. Moins du tiers des alertes (31%) répondaient à la définition des cas. Environ 82% ont recherché les contacts pendant l'investigation et 37% après les résultats. L'enquête a révélé qu'il y avait environ 6 contacts par cas suspect.

## Conclusion

Cette étude a permis de décrire le rôle des EIR et ESC qui constituent l'unité opérationnelle dans la riposte contre la Covid-19 en Côte d'Ivoire, et montré l'intérêt de mieux les organiser.

# Research article: Vaccination contre la Covid-19 en Côte d'Ivoire

**Published:** 2023-03-02

**Authors:** Harvey Attoh Touré, Issaka Tiembré, Joseph Bénié Bi Vroh, Kouadio Daniel Ekra

**Affiliations:** nstitut national d'hygiène publique (INHP), Abidjan, Côte d'Ivoire

**Keywords:** Vaccination, Covid-19, Côte d'Ivoire

En Côte d'Ivoire, 81 881 cas d'infection à Covid-19 et 799 décès ont été notifiés à la date du 22 avril 2022. La vaccination contre cette maladie a débuté le 1^er^ mars 2021 dans un contexte de rumeurs, de désinformation et de proximité de la date de péremption des doses de vaccins reçues dans le cadre de l'initiative COVAX. L'objectif de ce travail était de décrire la stratégie de vaccination contre la Covid-19 en Côte d'Ivoire.

## Méthodes

Il s'est agi d'une étude transversale à visée descriptive effectuée à partir des rapports de vaccination de la Direction de coordination du Programme élargi de vaccination du 1^er^ mars 2021 au 30 avril 2022.

## Résultats

La Côte d'Ivoire a reçu 21 112 120 doses de vaccins. La cible vaccinale initiale était constituée des personnes de plus de 18 ans. Au niveau national, 5 507 752 personnes étaient complètement vaccinées soit 28% de la population cible. L'engouement des populations était très faible avec moins de 2 000 doses administrées par jour avant mi-avril 2021. Le renforcement de la stratégie avancée de vaccination associé à une forte mobilisation sociale ont permis d'augmenter le nombre de doses administrées par jour à plus de 20 000. À partir du mois de décembre 2021, des campagnes de vaccination de masse de 15 jours ont été organisées, permettant d'administrer plus de 100 000 doses par jour et d'augmenter de façon significative les couvertures vaccinales.

## Conclusion

La Côte d'Ivoire a l'une des meilleures couvertures vaccinales de la sous- région. Toutefois, de nombreux défis restent à relever notamment l'accroissement de l'adhésion des populations.

# Research article: Hésitation vaccinale: les obstacles à une vaccination de masse contre la Covid-19 au Bénin

**Published:** 2023-03-02

**Authors:** Daleb Abdoulaye Alfa, Gilles Cottrell, Inès Boko, Aurore Atchade, Marc Fiogbé, Emmanuel Yovo, Sandrine Hounsa, Achille Massougbodji, Jean-Yves Le Hesran

**Affiliations:** UMR 261 IRD MERIT, Faculté de pharmacie, Paris, France

**Keywords:** Covid-19, Vaccination, Hésitation vaccinale, Bénin

## Introduction

Deux ans après le début de la pandémie de la Covid-19 qui a touché le monde, sous l’égide de l'OMS, des campagnes de vaccination de masse ont été mises en place dans tous les pays.

L'objectif est d'atteindre un seuil suffisant de vaccination pour bénéficier d'une immunité collective. Dans tous les pays, on se heurte à une part d'hésitation vaccinale. Il en est de même pour le Bénin où le ministère déclarait en novembre 2021, 3% de la population vaccinée.

Nous avons mis en place une étude en population générale pour étudier les freins à la vaccination.

## Méthodologie

La méthodologie adoptée est essentiellement qualitative. Nous avons réalisé des entretiens semi-directifs auprès des personnes non vaccinées et vaccinées. Un choix raisonné nous a permis de les identifier.

## Résultats

Nous avons interviewé 60 personnes non vaccinées et 20 personnes vaccinées. Autant d'hommes que de femmes. Il ressort des entretiens que plusieurs éléments rendent les gens méfiants. Certains s’étonnent de la rapidité avec laquelle le vaccin a été fabriqué en mettant en doute sa fiabilité. D'autres sont également dérangés par les mesures contraignantes édictées par le gouvernement telles que l'interdiction d'accès aux services publics pour les personnes non vaccinées. La majorité est rendue méfiante car le formulaire de consentement contient une clause de non-responsabilité du gouvernement et du fabricant en cas d'effets secondaires graves. Enfin, en zone rurale, les populations ont déclaré n'avoir pas assez d'informations sur les vaccins contre la Covid-19 et sur les lieux de vaccination.

## Discussion-conclusion

Notre étude confirme l'existence d'une hésitation forte à la vaccination contre la Covid-19 dans la population, d'une méfiance par rapport au vaccin lui-même et par rapport à la stratégie du gouvernement, mais aussi d'une défiance envers le gouvernement. De plus, les personnes déclarent avoir des doutes quant à la réalité de la maladie car elles et leur entourage ont été très peu concernées par la maladie.

# Research article: Mise en place d'un projet innovant « Eau, Hygiène, Assainissement » dans les quartiers informels du littoral et les communes isolées de Guyane en réponse à l’épidémie de Covid-19, 2020-2022

**Published:** 2023-03-02

**Authors:** Margot Oberlis, Étiennette Renoud-Grappin, Émilie Sevestre, Jean-Luc Bauza Attaher, Souleymane Gourgoudou, Charlène Cochet, Elena Guillen, Solène Wiedner-Papin, Karl Kpossou, Mélanie Gaillet, Florence Huber

**Affiliations:** Direction territoriale de la Croix-Rouge française, Cayenne, Guyane, France

**Keywords:** Précarité, Santé communautaire, Empowerment, Maladies hydriques, Maladies à transmission vectorielle

La Guyane française, territoire ultramarin d'Amazonie, est confrontée depuis 2 ans à l’épidémie de Covid-19. Cette dernière met à mal son système économique et sanitaire et les conditions de vie fragiles des personnes en situation de précarité. Les quartiers informels du littoral, illégaux, ne peuvent prétendre à une viabilisation par les services publics. Parallèlement, l'accès à l'eau potable dans les communes de l'intérieur est un enjeu quotidien du fait de leur situation isolée. Ainsi, maintenir un accès et assurer une gestion de cette ressource était essentiel pour éviter que des épidémies d'origine hydrique ou vectorielle ne viennent s'ajouter à la pandémie de SARS-CoV-2.

Dès septembre 2020, la Croix-Rouge a déployé un projet innovant en promotion de la santé, le projet WASH (*Water Sanitation Hygiene*, Eau, Hygiène Assainissement, EHA). Si des projets EHA sont couramment déployés dans les pays à ressources limitées, ils sont inhabituels sur le sol français.

En collaboration avec de nombreux partenaires, une équipe composée d'une cheffe de projet, de deux coordinatrices terrain et d'onze agents de santé publique issus de différentes communautés, a accompagné les populations des zones informelles du littoral et des villages isolés des communes de l'intérieur pour réduire les risques sanitaires aux niveaux individuel et collectif, *via* des actions de sensibilisation et de mobilisation sociale pour la mise en place d'actions par et pour les habitants.

Fin février 2022, 270 maraudes de sensibilisation et animations collectives sur les gestes barrières et la vaccination contre la Covid-19 ont été menées sur l'ensemble du territoire, touchant 8 800 personnes. De plus, 460 actions ont permis de sensibiliser 10 660 personnes sur le transport, le stockage et la potabilisation de l'eau, la gestion des déchets et la lutte contre les maladies entériques et vectorielles; 10 000 kits d'hygiène et 530 jerricans ont été distribués. Dix comités de quartier ont été accompagnés, de leur création à leur structuration afin de mettre en œuvre des actions collectives au sein des quartiers d'habitats spontanés.

Pensé initialement comme réponse à l'urgence sanitaire, le projet WASH a démontré, après 18 mois de déploiement, qu'il était possible d'améliorer les conditions de vie des populations précaires exclues des dispositifs de droit commun. L'implication des habitants *via* des comités de quartier a été un élément clé pour pérenniser les actions. Sorti de l'urgence Covid, le défi est aujourd'hui d'orienter le projet vers une approche de plus long terme, incluant l'EHA et d'autres thématiques prioritaires dans un objectif de promotion de la santé et d’*empowerment.*

# Research article: Développement d'un antivenin bivalent contre les envenimations scorpioniques sévères au Maroc

**Published:** 2023-03-02

**Authors:** Ines Hilal, Soukaina Khourcha, Mohamed Chablaoui, Abdelaziz Hmyene, Amal Safi, Naoual Oukkache

**Affiliations:** Institut Pasteur du Maroc, Casablanca, Maroc

**Keywords:** Envenimation, Scorpion, Antivenin, F(ab’)2, Réaction croisée

Au Maroc, l'envenimation scorpionique constitue un problème de santé publique majeur avec une incidence et une mortalité élevées dont les espèces incriminées sont *Androctonus mauretanicus (Am)* et *Buthus occitanus (Bo).* Plusieurs études ont montré que le traitement spécifique, soit l'immunothérapie, demeure la thérapeutique de choix indiquée après l'envenimation scorpionique, alors qu'au Maroc, seuls les traitements symptomatiques sont utilisés. Dans ce contexte, ces travaux visaient à développer un antivenin bivalent capable de neutraliser les envenimations létales au Maroc.

L'antivenin bivalent a été produit en immunisant les chevaux avec des venins bruts *Am* et *Bo* collectés dans différentes régions du Maroc. Les injections ont été faites par voie sous-cutanée à trois points au cou près du système lymphatique. Pour le contrôle de la production, nous avons utilisé l'immunodiffusion radiale. La capacité de neutraliser la létalité des venins *Am* et *Bo* a été estimée par ED50 chez la souris. La capacité de cet antivenin à neutraliser le venin de *lAndroctonus australis hector (Aah)* – un autre scorpion très dangereux au Maghreb – a également été étudiée.

L'antivenin bivalent produit contre un mélange de venins *Am* et *Bo* est capable de neutraliser l'effet mortel de l'Am et *Bo.* De plus, il présente une protection efficace contre le venin de scorpion *Aah* qui est incriminé dans les envenimations létales au Maghreb.

# Research article: Évaluation des pratiques de lutte contre les morsures de serpents auprès des agriculteurs à Mboka Polo, Kinshasa, République démocratique du Congo

**Published:** 2023-03-02

**Authors:** Sophie Bokata Masika, Jacques Ngambongo Mopili, Jackson Mbuango Malukisa, Hyppolyte Situakibanza, Thaddée Odio

**Affiliations:** Centre antivenimeux, Ministère de la Recherche scientifique et Université de Kinshasa, Kinshasa, République démocratique du Congo

**Keywords:** Morsure, Lutte contre les morsures de serpents (LCMS), Chapeaux à large bord, Enquête CAP, RD Congo

## Introduction

Dans le cadre de la lutte contre les morsures de serpents, une enquête portant sur la connaissance, l'attitude et la pratique a été réalisée par visite à domicile. Cette étude a permis d'identifier les principaux moyens de lutte contre les morsures de serpents.

## Matériel et méthodes

Il s'agit d'une étude prospective menée à l'aide d'un questionnaire et d'une interview destinés aux agriculteurs de Mboka Polo. Une rue sur 3 et une parcelle sur 3, et toutes les maisons dans la parcelle, ont fait l'objet de notre enquête sur une période allant du 3 juin au 3 août 2021.

## Résultats

Sur 204 enquêtés, le sexe féminin était beaucoup plus représenté avec 58,8% contre 41,2% pour le sexe masculin. L'enquête montre que l'assainissement du milieu ainsi que le port de gants, de bottes et de chapeaux à large bord représentent les moyens de lutte contre les morsures de serpents (LCMS) sous réserve d'un approvisionnement régulier, et à des prix abordables.

## Conclusion

Ce moyen de lutte représente un avantage pour se préserver contre les morsures de serpents concernant les habitants de Mboka Polo. Se limitant seulement à Mboka Polo, cette étude mérite d’être élargie en République démocratique du Congo pour prévenir les éventuelles morsures.

# Research article: Déterminants et prédicteurs de la déclaration des erreurs médicamenteuses chez les professionnels de santé à l'Institut national de nutrition et de technologie alimentaire à Tunis (INNTA): une étude transversale basée sur le Health Belief Model (HBM)

**Published:** 2023-03-02

**Authors:** Aya Ahmed El Husseiny Shaaban

**Affiliations:** Université Sédar Senghor, Alexandrie, Égypte

**Keywords:** Erreurs médicamenteuses, Perceptions des professionnels de santé, Déclaration des EM, Système de signalement des EM, Health Belief Model (HBM)

De nombreuses erreurs passent inaperçues ou ne provoquent aucun effet indésirable. Cependant, une seule erreur médicamenteuse (EM) peut provoquer un évènement grave pour le patient. La sous-déclaration des erreurs médicamenteuses par les professionnels de santé est un problème qui menace la sécurité de la prise en charge médicamenteuse du patient dans le monde entier. L'incapacité des systèmes de santé à tirer les leçons de leurs erreurs est une cause majeure de répétition de ces erreurs. Plusieurs facteurs peuvent entraver le processus de la déclaration, tels qu'une information insuffisante sur le système de déclaration, le sentiment d'inutilité du processus ou la peur de la sanction. L'objectif de notre étude est d'identifier les déterminants de la déclaration des EM et, plus précisément, d’étudier les prédicteurs de l'intention des professionnels de santé de déclarer les EM à l'Institut national de nutrition et de technologie alimentaire de Tunis (INNTA).

Une étude transversale utilisant un questionnaire auto-administré a été menée auprès d'un échantillon exhaustif (122 personnes interrogées) des professionnels de santé de l'INNTA) pendant les mois de mars à août 2021 pour évaluer leurs connaissances, leurs pratiques et leurs perceptions vis-à-vis du signalement des EM à l'aide du Modèle de croyances en santé ou Health Belief Model (HBM). Des analyses univariées en utilisant le test exact de Fisher et multivariées en utilisant le modèle de régression logistique ont été effectuées.

Le taux de réponse était de 92,42%. Le pourcentage le plus élevé de participants (38,52%) était celui des professionnels de santé ayant un niveau de connaissance moyen. Plus des trois quarts des professionnels de santé de l'INNTA (87,7%) avaient été témoins d'une erreur médicamenteuse, mais très peu l'avaient officiellement signalée (3,74%), mettant en évidence la problématique de la sous-déclaration des EM au sein de l'INNTA. Les étapes de la prescription et de l'administration du médicament étaient le plus souvent mises en cause. L'absence de système de déclaration, la crainte de la sanction et la culture du blâme étaient les obstacles le plus fréquemment perçus par les professionnels de santé. Les déterminants significativement associés à la déclaration selon l'analyse univariée étaient la profession, la vulnérabilité perçue et la gravité perçue (p < 0,05). Cependant, les prédicteurs significatifs de l'intention (p < 0,05) étaient le sexe féminin, l'auto-efficacité perçue élevée, la vulnérabilité perçue élevée et l'ancienneté à l'INNTA, qui est inversement associée.

Avec cette étude, nous avons pu déterminer les prédicteurs du comportement du personnel de santé à l'INNTA vis-à-vis du signalement des EM. Ces résultats ne sont cependant pas généralisables, ne concernant qu'un échantillon exhaustif de l'INNTA. Un programme de formation et de sensibilisation aux EM et à leur déclaration est envisagé pour les professionnels de santé ainsi que la mise en place d'un système officiel de déclaration des EM au sein de l'Institut.

# Research article: Facteurs associés à la vaccination incomplète des enfants de 15 à 23 mois dans le district sanitaire de Tominian au Mali en 2020

**Published:** 2023-03-02

**Authors:** Ousmane Boua Togola, Oumar Sangho, Yacouba Koné, Djibril Barry, Pauline Kiswensida Yanogo, Yaya Ballayira, Hanine Kéita, Bouyagui Traoré, Habibatou Idé, Souleymane Coulibaly, Ousmane Abdoul Aziz Dicko, Fadima Radhia Diallo

**Affiliations:** Sous-direction Lutte contre la maladie de la Direction générale de la Santé et de l'hygiène publique, Bamako, Mali

**Keywords:** Vaccination incomplète, Enfants, Facteurs associés, Tominian, Mali

## Introduction

La vaccination permet d'immuniser les enfants contre les maladies meurtrières et invalidantes. Selon l'Organisation mondiale de la Santé, la plupart des enfants incomplètement vaccinés se trouvent en Afrique. Au Mali, 55% des enfants de 12-23 mois étaient complètement vaccinés et 14% n'avaient reçu aucun vaccin en 2018. Notre étude avait pour objectif de déterminer les facteurs associés à la vaccination incomplète des enfants de 15-23 mois de Tominian dans un contexte d'insécurité en 2020.

## Méthodes

Nous avons mené une étude transversale analytique sur 508 mères d'enfants âgés de 15 à 23 mois. Un échantillonnage stratifié à trois degrés a permis d'identifier les aires de santé, villages et mères d'enfants. Nous avons testé l'association entre la vaccination et ses déterminants probables en utilisant une régression logistique multiple à un niveau de signification de 0,05 et un intervalle de confiance à 95% (IC_95%_) à l'aide d'Epi Info 7.2.2 et de Medcalc. Le test de Hosmer et Lemeshow a été utilisé pour juger l'adéquation du modèle de régression multiple obtenu après l'analyse multivariée.

## Résultats

L’âge moyen des mères et des enfants était respectivement de 27 ± 6 ans et de 19 ± 3 mois. La couverture vaccinale de la 2^e^ dose de vaccin antirougeoleux était de 34,84%. La méconnaissance de l'importance de la vaccination (ORa [IC_95%_] = 9,34 [2,15-40,58], p = 0,0029), la connaissance de moins de 5 maladies cibles de la vaccination (ORa [IC_95%_] = 3,80 [1,42-10,17], p = 0,0078), l'absence de revenus (ORa [IC_95%_] = 2,96 [1,68-5,21], p = 0,0002), le temps d'attente long (ORa [IC_95%_] = 2,38 [1,52-3,72], p = 0,0002), l'accouchement à domicile (ORa [IC_95%_] = 2,26 [1,46-3,50], p = 0,0002) et la non-tenue de causeries éducatives par les vaccinateurs (ORa [IC_95%_] = 1,79 [1,15-2,78], p = 0,0092) étaient statistiquement associés à la vaccination incomplète des enfants.

## Conclusion

La couverture vaccinale n’était pas associée à l'insécurité. Nous recommandons de mettre en œuvre des activités de rattrapage vaccinal de sensibiliser et de soutenir la création d'activités génératrices de revenus pour les mères afin de contribuer à l'amélioration du statut vaccinal des enfants.

# Research article: Les pharmaciens acteurs de l'alerte

**Published:** 2023-03-02

**Authors:** Jean-Loup Rey, Pascal Millet, Jean-Marie Milleliri

**Affiliations:** GISPE / ReMeD, Villeneuve (04), France

**Keywords:** Alerte, Veille, Pharmaciens

En 1981, c'est une demande accrue de pentaminide à la pharmacie des CDC qui sonne l'alerte du sida, ce médicament étant le seul capable de traiter les pneumonies à *Pneumocystis.*

En 1986, la consommation « anormale » d'amikacine dans un hôpital signe une épidémie d’*Acinetobacter baumanni* phénotype APH3’. En décembre 2013, une demande accrue d'immunoglobulines à la pharmacie du CH de Tahiti signe l'importance de l’épidémie de Zika, ce médicament étant utilisé pour les syndromes de Guillain Barré, complication du Zika. Lors de l’épidémie d'hépatite E à Zinder en 2017, la consommation des différents médicaments a été étudiée en parallèle avec le nombre de cas, certains médicaments témoignant d'une augmentation de leur utilisation avant le pic des cas. Ces faits montrent que le secteur pharmaceutique (hospitalier ou non) peut jouer un rôle important dans la veille et l'alerte. Il s'agit d'une partie de la pharmaco-épidémiologie qui est sous-utilisée actuellement et qui mérite d’être valorisée.

# Research article: Prédiction de l'adaptation urbaine d’ *Anopheles gambiae* et d’ *Anopheles coluzzii*, principaux vecteurs du paludisme, en Afrique centrale

**Published:** 2023-03-02

**Authors:** Neil Michel Longo Pendy, Claire Sangbakembi Ngounou, Pierre Kengne, Carlo Costantini, Diego Ayala

**Affiliations:** Centre interdisciplinaire de recherches médicales de Franceville (CIRMF), Franceville, Gabon

**Keywords:** Complexe *Anopheles gambiae*, Habitat larvaire, Adaptation urbaine, Prédiction, Paludisme

En Afrique centrale, la récente prolifération des anophèles en milieu urbain menace les efforts de lutte contre le paludisme. *Anopheles coluzzii* et *Anopheles gambiae*, vecteurs majeurs de paludisme en Afrique, ont surmonté les pressions anthropiques à l'exemple de la pollution, par des changements physiologiques et génétiques. Ici, nous avons étudié les facteurs écologiques et environnementaux qui ont favorisé leur expansion urbaine afin de prédire les processus adaptatifs convergents. Un échantillonnage exhaustif et systématique de 380 habitats larvaires d'anophèles a été réalisé dans quatre grandes villes d'Afrique centrale. Une large série de paramètres physico-chimiques et environnementaux a été analysée. Des analyses statistiques ont été effectuées afin d’étudier la distribution de ces deux espèces et de prédire les modèles d'adaptation urbaine. Les quatre villes offraient une grande mosaïque commune d'habitats larvaires qui étaient colonisés par les deux espèces, montrant ainsi leur extraordinaire plasticité écologique. Néanmoins, *An. coluzzii* était prédominant dans les habitats urbains côtiers, tandis qu'An. *gambiae* se trouvait à l'intérieur des terres. Cette différence était déterminée par quelques facteurs environnementaux, notamment le potentiel d'hydrogène, la densité de bâti, la proximité d'une source d'eau permanente, l'oxygène dissous et la présence d'algues. Notre approche prédictive a permis de trouver des schémas communs d'adaptation urbaine entre les villes, mais cela correspond à un modèle de sélection dépendant de la fréquence relative *d'Anopheles coluzzii* par rapport à *Anopheles gambiae* entre les habitats. Dans l'ensemble, nos résultats fournissent une base afin de comprendre le processus d'adaptation urbaine chez deux vecteurs majeurs du paludisme, avec des conséquences importantes pour les stratégies actuelles et futures de contrôle du paludisme.

# Research article: Diversité génétique et multiplicité de l'infection à *Plasmodium falciparum* durant l’épidémie du paludisme dans les régions d'Anosy, d'Atsimo-Atsinanana et d'Ihorombe, Madagascar

**Published:** 2023-03-02

**Authors:** Voahangy Andrianaranjaka, Élisabeth Ravaoarisoa, Berthin Rakotoniarivo, Tovonahary Rakotomanga, Fanomezantsoa Ralinoro, Célestin Razafinjato, Jocelyn Ratovonjato, Arsène Ratsimbasoa

**Affiliations:** Mention Biochimie fondamentale et appliquée, Faculté des sciences, Université d'Antananarivo, Antananarivo, Madagascar

**Keywords:** *Plasmodium falciparum*, Diversité génétique, Épidémie, Madagascar

Le paludisme, une maladie parasitaire curable mais mortelle, représente un des problèmes majeurs de santé publique à l’échelle mondiale. La surveillance épidémiologique moléculaire régulière de la diversité génétique des populations plasmodiales et de la multiplicité de l'infection est d'une importance cruciale pour l’évaluation des activités de lutte mises en œuvre et pour l'orientation et/ou l'implantation de nouvelles stratégies de lutte en vue de l’élimination du paludisme. En effet, notre étude a pour objectif d'estimer la prévalence de l'infection à *Plasmodium falciparum* et d’étudier la diversité génétique et la multiplicité de l'infection (MOI) à *P. falciparum* lors d'une épidémie du paludisme dans trois régions de Madagascar.

La prévalence de l'infection à *P. falciparum* a été estimée par la PCR nichée à partir de 540 prélèvements collectés lors d'une activité de riposte suite à l’épidémie du paludisme dans le district d'Amboasary Atsimo de la région d'Anosy, dans le district d'Iakora de la région d'Ihorombe et dans les districts de Vangaindrano et de Vondrozo de la région d'Atsimo-Atsinanana en 2019. Les gènes *Msp1* et *Msp2* ont été analysés pour étudier la diversité génétique et la MOI de l'infection à *P. falciparum.*

Par la PCR nichée, la prévalence de l'infection à *P. falciparum* était de 64,6% [IC95%: 60,4-68,6%] (349/540). Le typage des gènes *Msp1* et *Msp2* a montré que les trois familles alléliques MAD20, K1 et RO33 de *Msp1* et les deux familles alléliques 3D7 et FC27 de *Msp2* sont présentes dans les sites d’étude. Pour le gène *Msp1*, 267 échantillons ont été amplifiés avec succès et 54 allèles distincts ont été identifiés dont la famille allélique K1 prédomine avec une fréquence de 74,2% suivie de MAD20 à 51,7% et R033 à 50,9%. Pour le gène *Msp2*, les 255 isolats amplifiés avec succès sont regroupés en 78 allèles avec la prédominance de la famille allélique 3D7 à 70,6% contre 65,1% pour FC27. Une grande diversité génétique des isolats de *P. falciparum* analysés a été observée. Le taux des infections polyclonales est de l'ordre de 72% avec une valeur moyenne de la MOI de 2,5 ± 1,3 dans l'ensemble des échantillons analysés.

Notre étude a montré un grand polymorphisme allélique de l'infection à *P. falciparum* lors de l’épidémie du paludisme dans les districts d'Amboasary Atsimo, d'Iakora, de Vangaindrano et de Vondrozo en 2019.

# Research article: Recrudescence des cas de paludisme dans le Sud et la marge des hautes terres centrales de Madagascar en 2018 et 2019

**Published:** 2023-03-02

**Authors:** Élisabeth Ravaoarisoa, Voahangy Andrianaranjaka, Isabelle Hanitriniaina, Ginat Andoniaina Ramahazomanana, Tovonahary Angelo Rakotomanga, Fanomezantsoa Ralinoro, Célestin Razafinjato, Jocelyn Ratovonjato, Arsène Ratsimbasoa

**Affiliations:** Mention Biochimie Fondamentale et Appliquée, Faculté des Sciences, Université d'Antananarivo, Antananarivo, Madagascar

**Keywords:** *Plasmodium falciparum*, *Msp1*, *Msp2*, Recrudescence, Madagascar

## Contexte

Face à la recrudescence des cas de paludisme enregistrée en 2018 et 2019, des investigations parasitologiques ont été effectuées dans les Districts de Betroka (Sud) et Fenoarivobe (marge des hautes terres centrales). L'objectif est de caractériser et de comparer la diversité génétique des isolats de *Plasmodium falciparum Msp1* et *Msp2* circulants pendant ces périodes.

## Méthodologies

Un dépistage actif du paludisme basé sur le test rapide (TDR) a été effectué en mai 2018 et août 2019 à Betroka, en août et novembre 2019 à Fenoarivobe. Des gouttes de sang capillaire sur papiers buvards ont été collectées chez des villageois volontaires pour le diagnostic moléculaire par la PCR nichée. Les isolats confirmés positifs à *P. falciparum* ont été utilisés pour le génotypage de *Msp1* et *Msp2* avec leurs familles alléliques respectives.

## Résultats

Au total, 199 échantillons de Betroka ont été analysés dont 86 en mai 2018 et 133 en août 2019. La proportion de l'infection plasmodiale, que ce soit par le TDR ou par la PCR, est significativement élevée en 2019 par rapport à 2018 (p = 0,001). En combinant les marqueurs *Msp1* et *Msp2*, la valeur moyenne de la multiplicité de l'infection (MOI) est de 2,9 ± 1,7 et de 4,1 ± 2,5 respectivement en mai 2018 et août 2019 (p = 0,016). Pendant les deux périodes de l’étude, les familles alléliques K1 (*Msp1*) et 3D7 (*Msp2*) sont prédominantes. À Fenoarivobe, 162 échantillons ont été collectés dont 100 en août et 62 en novembre 2019. Le taux de positivité par le TDR et la PCR est faible en novembre 2019 comparé à celui du mois d'août 2019 (p = 0,001). La moyenne de la MOI est de 2,2 ± 2,0 et de 2,4 ± 1,8 respectivement en août et novembre 2019 (p = 0,584). Pour *Msp1*, la famille allélique K1 est prédominante pendant les 2 périodes de l’étude. Pour *Msp2*, la famille allélique FC27 est prédominante en août 2019 et 3D7 en novembre 2019 (p = 0,03). Conclusion. Betroka se situe dans la zone subdésertique où la transmission s'apparente au faciès sahélien. Cependant ces résultats suggèrent une transmission persistante même en saison sèche. Le changement de la prédominance des familles alléliques pourrait indiquer que les souches de *P. falciparum* circulantes à Fenoarivobe pendant les 2 périodes sont différentes. La transmission plasmodiale est dynamique, aussi une surveillance intégrée (parasitologique, entomologique, anthropologique et climatique) est nécessaire pour mieux comprendre la dynamique locale des parasites en vue d'adapter ou de proposer des interventions appropriées.

# Research article: Taux élevés de paludisme autochtone en milieu urbain à Toliara, dans la zone subaride du Sud-Ouest de Madagascar

**Published:** 2023-03-02

**Authors:** Dina Ny Aina Liantsoa Randriamiarinjatovo, Viviane Razafindravao, Rosia Yolande Nomenjanahary, Seheno Razanatsiorimalala, Milijaona Randrianarivelojosia

**Affiliations:** Unité de parasitologie, Institut Pasteur de Madagascar, Antananarivo, Madagascar

**Keywords:** Paludisme urbain, *Plasmodium falciparum*, *P. vivax*, *P. ovale*, Madagascar

Sur l’île de Madagascar, le paludisme demeure un problème de santé publique. Dans la partie subaride du Sud et du Sud-Ouest, avec une précipitation moyenne annuelle de 300 mm, le niveau de transmission du paludisme est faible. L’élimination de cette maladie dans les villes de cette partie de l’île est envisageable. Cependant, il est crucial de documenter le paludisme importé (associé à la mobilité humaine) et le paludisme autochtone afin de guider le ministère de la Santé dans le choix stratégique visant l’élimination progressive du paludisme. Ainsi, nous avons mené une étude dans la ville de Toliara, le chef-lieu de la région Sud-Ouest de Madagascar de janvier à décembre 2021. Pour les patients suspects de paludisme vus en consultation à la Clinique catholique Betela Andaboly et dans le Centre de santé de base Sanfily, des frottis sanguins (frottis minces et gouttes épaisses) colorés au Giemsa ont été examinés par des techniciens expérimentés. Sur les 805 patients suspects de paludisme vus en consultation, l'infection plasmodiale a été confirmée chez 16,7% (IC95%: 14,3-19,6%; n = 135) d'entre eux. La médiane de la parasitémie a été de 1,397 trophozoïte/µl. Le taux de portage de gamétocytes a été de 0,02 (18/805). La majorité des patients impaludés (60%) ont été âgés de 15 ans et plus. Le paludisme à *P. falciparum* a été le plus fréquent (88,1%: 119/135). Deux cas à *P. vivax* et un cas à *P. ovale* ont été détectés. En outre, 42% (54/135) des patients impaludés n'ont pas quitté la ville de Toliara au cours des 3 mois précédant la consultation médicale. Ces résultats démontrent la persistance de la transmission autochtone du paludisme à Toliara. Les cas de paludisme importés (75/135) provenaient de la zone irriguée Toliara II et des communes rurales de Sakaraha et de Morombe. Afin de rendre réelle l’élimination progressive du paludisme en milieu urbain dans la zone subaride de Madagascar, il faut certes diagnostiquer et traiter les cas de paludisme, et mettre en place un mécanisme permettant de détecter des cas secondaires au sein et autour des foyers des malades vus en consultation. Mais il est également impératif d'améliorer le taux d'utilisation des moustiquaires imprégnées d'insecticide, car une grande partie de la population urbaine vit dans des maisons traditionnelles. L'impact des passages des cyclones dans le Sud de Madagascar en 2022 sera abordé dans notre présentation.

# Research article: Prévalence des espèces de *Plasmodium* spp chez le moustique Anophèle dans les districts de Gama Tsé-tsé et Brazzaville, en République du Congo

**Published:** 2023-03-02

**Authors:** Mbama Ntabi Jacques Dollon, Abel Lissom, Jean-Claude Djontu, Steve Diafouka Kietela, Jolivet Mayel, Francis Nongley Nkemngo, Romuald Agonhossou, Daniel Nguete Nguiffo, Cyrille Ndo, Romaric Akoton, Térence Boussougou-Sambe, Luc Djogbénou, Charles Wondji, Ayola Akim Adegnika, Arsène Lenga, Steffen Borrmann, Francine Ntoumi

**Affiliations:** 37 rue Mouloki, Makazou (Mfilou), Brazzaville, République du Congo

**Keywords:** Prévalence, Vecteurs *Anophèles*, *Plasmodium* spp, République du Congo

La distribution des vecteurs Anophèles, des *Plasmodiums* et de l'homme est responsable du paludisme, une maladie prioritaire de santé publique en République du Congo. Contrairement à ces zones où l’épidémiologie du paludisme a été mieux étudiée pour la mise en place des stratégies de lutte, aucune étude n'existe sur ce sujet en République du Congo. L'objectif est de déterminer la prévalence des espèces de *Plasmodium* spp chez les Anophèles femelles. Une enquête entomologique longitudinale de 7 mois a été menée dans la région du Pool (Ntoula, Djoumouna) et la région de Brazzaville (Mayanga) de mars à septembre 2021. Les moustiques ont été collectés dans la faune résiduelle des ménages intérieurs entre 5 h et 10 h. La capture des moustiques a été réalisée à l'aide de l'aspirateur mécanique, ces moustiques ont été identifiés morphologiquement. Toutes les Anophèles femelles ont été extraites à l'aide de la méthode Livak. Certaines espèces du complexe *Anopheles gambiae* et du groupe *Anopheles funestus* ont été confirmées à l'aide de la réaction en chaîne par polymérase. L'identification des Anophèles femelles infectées a été effectuée à l'aide de la méthode TaqMan de RT-PCR. Un total de 495 moustiques vecteurs du paludisme comprenant *Anopheles gambiae* sl 96,6% (478/495), *Anopheles funestus* 2,6% (13/495), *Anopheles moucheti* 0,8% (04/495) ont été identifiés. Cent cinquantetrois extraits d'ADN de moustique choisis au hasard ont confirmé la prédominance d'An. *gambiae s.s* dans les localités étudiées. La prévalence de *Plasmodium ssp* chez les moustiques Anophèles était de 23,4% (116/495). La mono-infection à *P. falciparum* (58,6%; 68/116) était prédominante, suivie par *P. malariae* et *P. ovale* (1,7%; 2/495 pour chaque espèce). Les cas de co-infection ont été détectés à raison de 25,0% (29/116) *pour P. falciparum/P. malariae* et 7,8% (9/116) pour *P. falciparum/P. ovale.* La triple infection par *P. falciparum/P. malariae/P. ovale* a été détectée de 5,2% (06/116) chez les moustiques infectés par *Plasmodium*. Les résultats ont révélé un taux élevé d'infection à *Plasmodium* chez les principaux vecteurs Anophèles, *An. gambiae* enregistrant les taux d'infection les plus élevés. L'identification d'espèces non *Plasmodium falciparum* chez les moustiques Anophèles met en évidence la nécessité de renforcer les stratégies de lutte antivectorielle dans ces zones de la République du Congo.

# Research article: Connaissances, attitudes et pratiques des mères d'enfants de 0 à 59 mois sur le paludisme à Bangui, République centrafricaine

**Published:** 2023-03-02

**Authors:** Alexandre Manirakiza, Asaël Sakanako, Jean de Dieu Longo, Marcel Mbeko Simaleko, Henri Saint-Calvaire Diemer, Gérard Grésenguet

**Affiliations:** Institut Pasteur de Bangui & Université de Bangui, Faculté des sciences de la santé, République centrafricaine

**Keywords:** Enquête CAP, Paludisme, Mère, Enfants, Bangui

En République centrafricaine, le paludisme est un problème majeur de santé publique car il représente l'une des premières causes de morbidité et de mortalité. Ce sont les enfants âgés de moins de 5 ans et les femmes enceintes qui sont les plus vulnérables. L'objectif est d’évaluer les connaissances, attitudes et pratiques (CAP) des mères d'enfants âgés de 0 à 59 mois face au paludisme. Nous avons réalisé une étude transversale descriptive et analytique menée dans la ville de Bangui, capitale de la République centrafricaine. La taille de l’échantillon était de 152 femmes. L’âge moyen de ces femmes était de 27 ans et variait de 15 à 49 ans. Parmi ces femmes, 29,6% avaient un niveau d’étude secondaire, 68,4% étaient célibataires et 38,8% étaient des ménagères. Le signe principal (la fièvre ou corps chaud) a été cité dans 23,6% des cas. Cependant la fièvre forte était le signe du paludisme grave le plus connu (84,2%). La transmission du paludisme était citée par 94% des mères. Il existait une bonne connaissance des mesures préventives avec 94,7% des mères qui donnaient la moustiquaire imprégnée comme moyen de prévention, alors qu'une proportion de 57% des mères a cité au moins 2 des moyens suivants: moustiquaire imprégnée, spray anti-moustique, pulvérisation intra-domiciliaire et destruction des gîtes larvaires. Plus de la moitié des femmes interrogées (54%) disait avoir recours à la structure sanitaire en cas de paludisme à cause d'une meilleure prise en charge et d'un coût accessible. Il existait une relation significative entre le niveau de connaissances du paludisme en fonction de l’âge (> 25 ans) et le statut matrimonial (femmes mariées) de la mère, ainsi que le recours au centre de santé pour une prise en charge en fonction du niveau d'instruction (p < 0,05). À l'issue de cette étude, nous suggérons au programme national de lutte contre le paludisme de renforcer les stratégies de sensibilisation afin d'amener les populations à améliorer leur connaissance en matière de paludisme et à changer de comportement mais aussi de mener des enquêtes dans d'autres régions du pays pour compléter cette étude épidémiologique.

# Research article: Évaluation de la distribution aux femmes enceintes et de la bio-efficacité des moustiquaires imprégnées à longue durée d'action à l’échelle du Bénin

**Published:** 2023-03-02

**Authors:** Gilles Cottrell, Christophe Soares, Aziz Bouraima, Marc Fiogbé, Seun Egbinola, Armel Djènontin

**Affiliations:** Centre de recherche entomologique de Cotonou (CREC), Centre de recherche et de lutte contre les maladies infectieuses (CReMIT), Université d'Abomey-Calavi (UAC), Cotonou, Bénin

**Keywords:** Femmes enceintes, Consultation prénatale, Moustiquaires, Intégrité physique, Bio-efficacité

## Contexte

L'utilisation des moustiquaires imprégnées d'insecticide à longue durée d'action (MILDA) est l'un des piliers de la lutte contre le paludisme chez la femme enceinte. La présente étude a été initiée en 2018 au Bénin pour évaluer à l’échelle nationale la distribution, l'utilisation, l'intégrité physique et la bio-efficacité des MILDA utilisées par les femmes enceintes (FE) pendant leur grossesse.

## Matériel et méthodes

Les données ont été collectées auprès de 718 FE dans 12 centres de santé publics et privés en zones urbaines et rurales dans les trois régions Sud, Centre, et Nord du Bénin. Les FE ont été recrutées au centre de santé lors de leur première consultation prénatale (CPN1) et visitées deux fois à leur domicile à 1 mois d'intervalle. Au cours de ces visites, les FE ont répondu à un questionnaire permettant de collecter des données sur leurs caractéristiques générales et sur leur utilisation de la MILDA. À la deuxième visite, la MILDA retrouvée sur leur unité de couchage a été retirée pour des tests au laboratoire et remplacée par une MILDA neuve.

## Résultats

La possession et l'utilisation des MILDA ont été de 94% (intervalle de confiance à 95% (IC95%) [92%-96%]), et de 93% (IC95%, [91%-95%]) respectivement chez les FE avant leur CPN1. À la CPN1, 63% (IC95%, [59%-67%]) des FE avaient reçu une MILDA et seulement 12% (IC95%, [10%-14%]) la plaçaient sur leur unité de couchage. En outre, 67% (IC95%, [63%-71%]) des MILDA retrouvées sur les unités de couchage des FE étaient vieilles de plus d'un an. Parmi ces MILDA, 61% (IC95%, [57%-65%]) étaient en bon état physique et 39% (IC95%, [35%-43%]) étaient bio-efficaces avec une variabilité selon le statut public ou privé des centres de santé, le milieu rural ou urbain et le site Sud, Centre ou Nord.

## Conclusion

La possession et l'utilisation des MILDA par les FE avant la CPN1 se sont avérées très satisfaisantes. Par contre la distribution des MILDA aux FE dans les centres de santé lors de la CPN1 n’était pas optimale et seule une petite minorité de FE parmi celles qui avaient reçu une MILDA lors de cette CPN l'utilisait. Par conséquent, une forte proportion des FE utilisait des MILDA pas en bon état et/ou non bio-efficaces. Ces résultats suggèrent la nécessité de revoir la stratégie de sensibilisation des FE lors des CPN.

# Research article: Évolution des cas déclarés de paludisme et des décès au Togo de 2008 à 2017

**Published:** 2023-03-02

**Authors:** Tchaa Abalo Bakaï, Anne Thomas, Tina Atcha-Oubou, Tchassama Tchadjobo, Jean Iwaz, Nagham Khanafer, Muriel Rabilloud, Nicolas Voirin

**Affiliations:** Unité Surveillance épidémiologique & Recherche opérationnelle et fondamentale, Programme national de lutte contre le paludisme (PNLP-Togo), Lomé, Togo

**Keywords:** Togo, Paludisme, Surveillance, Mortalité, Épidémiologie

## Introduction

Le rapport 2019 de l'OMS sur le paludisme indiquait qu’« à l’échelle mondiale, le fardeau du paludisme était inférieur à 10 il y a quelques années, mais ces dernières années, le nombre de cas de paludisme a augmenté dans le monde ». Cette étude a rapporté des changements dans les cas de paludisme et les décès au niveau national du Togo (2008-2017) et présenté les résultats de la lutte anti-paludique et les chances d’éradication d'ici 2030 au Togo.

## Méthodes

Les cas et les décès ont été recueillis à partir des dossiers médicaux, des rapports mensuels et des notes de 6 000 travailleurs du domaine de la santé; puis résumés par région, district, année et sous-populations spécifiques. Les estimations des tendances annuelles du nombre de cas et de décès ont été obtenues au moyen de modèles linéaires généralisés.

## Résultats

Le nombre de cas a été multiplié par 4 alors que la population a augmenté de 1,3 fois (2008-2017). Les augmentations ont touché toutes les régions et les différentes sous-populations. Les augmentations relatives annuelles moyennes chez les enfants de < 5 ans, les femmes enceintes et les autres personnes étaient de 13%, 10% et 14%, respectivement. Les taux de mortalité ont diminué dans toutes les régions et les diminutions relatives annuelles moyennes des sous-populations étaient de 7%, 16% et 6%, respectivement.

## Conclusion

Les augmentations des cas de paludisme et la diminution des décès étaient associées à des investissements plus élevés et à un meilleur suivi des cas. Ce résultat est en faveur d'une amélioration de la prévention, du diagnostic et du traitement. Ces tendances devraient inciter les gouvernements et partenaires à maintenir ou intensifier les efforts actuels pour atteindre les objectifs d’élimination d'ici 2030.

# Research article: Facteurs associés à la non-évocation du paludisme d'importation: une étude monocentrique rétrospective

**Published:** 2023-03-02

**Authors:** Christine Roves, Luc Labarbe, Cécile Ficko, Pierre-Louis Conan

**Affiliations:** Service des maladies infectieuses et tropicales, Hôpital d'instruction des armées Bégin, Saint-Mandé, France

**Keywords:** Paludisme d'importation, Voyage, Médecine générale

## Introduction

Le médecin généraliste est en première ligne dans la prise en charge des accès fébriles de retour de voyage. Du fait de sa rareté et de sa clinique protéiforme, le paludisme d'importation représente toujours un défi diagnostique.

## Matériel et méthodes

Étude descriptive monocentrique rétrospective de 2007 à 2019 de tous les cas de paludisme d'importation prouvés microbiologiquement ayant été pris en charge dans notre hôpital. L'objectif était de décrire l'attitude diagnostique et thérapeutique avant la prise en charge hospitalière et de rechercher des facteurs associés au non-diagnostic de paludisme. Une régression logistique uni et multivariée a été réalisée afin de mettre en évidence les facteurs associés. Une analyse de sensibilité sur données imputées a été réalisée en analyse secondaire.

## Résultats

Cinq cent quatre-vingts patients (580) ont été inclus dans l'analyse (âge moyen 39 (± 14) ans, 406/576 (70%) d'hommes). Le motif principal de séjour était la visite à la famille ou à des amis (277/552 (50%)). Quatre cent quatre-vingt-dix patients avaient consulté un médecin généraliste ou un service d'urgences après l'apparition des symptômes.

Les populations consultant en ville ne diffèrent pas significativement de celles consultant aux urgences. En ville, le patient consulte en moyenne 12,58 jours après son retour soit 5,14 jours après l'apparition de ses premiers symptômes. Le diagnostic est évoqué en ville dans 57,2% et aux urgences dans 97,6% des cas, soit un diagnostic non évoqué chez 67 patients au total.

En ville, un bilan biologique est prescrit dans 48,1% des cas avec une goutte épaisse dans 40,6% des cas. Il n'y a pas d'impact statistiquement significatif sur la gravité clinique ou biologique si la consultation a été réalisée en ville ou aux urgences.

En multivarié, le seul facteur associé à la non-évocation du paludisme était une consultation chez le médecin généraliste (OR ajusté 48,8 [IC95%: 19-152], p < 0,001). L'analyse de sensibilité trouvait les mêmes résultats (OR ajusté 31,9 [15,6-73,3], p < 0,001). La prise d'une chimioprophylaxie et/ou une consultation préalable dans un centre de vaccinations internationales n’étaient pas associées à un meilleur taux de diagnostic.

## Discussion

Les limites de notre travail sont sa conception rétrospective et monocentrique. Nous n'avons pas pu affiner la recherche des déterminants de non-diagnostic. Il est probable que la demande systématique d'un voyage récent dans le questionnaire standardisé de l'infirmier d'accueil et d'orientation des urgences explique la proportion plus importante de diagnostic de paludisme d'importation.

## Conclusion

Un renforcement de la formation médicale initiale et continue sur les thèmes de la santé des voyageurs contribuerait probablement à l'amélioration des diagnostics des paludismes d'importation.

# Research article: Positionnement de la technique Illumigen^®^ en analyse de première intention dans le cadre du dépistage du paludisme d'importation: un retour d'expérience

**Published:** 2023-03-02

**Authors:** Lucie Menier, Bruno Pradines, Julie Plantamura, Anne-Margaux Dejean, Rémy Amalvict, Barbara Foucher

**Affiliations:** Fédération des laboratoires, Hôpital d'instruction des armées Sainte-Anne, Toulon, France

**Keywords:** Illumigen^®^, LAMP (Loop-Mediated isothermal Amplification), Paludisme, Parasitémie

## Introduction

Au sein de notre laboratoire de biologie médicale, la technique Illumigen^®^ est utilisée en première intention pour le dépistage du paludisme. En cas de résultat positif, des techniques complémentaires sont réalisées afin de déterminer l'espèce et la parasitémie. Dans le cas contraire, nous excluons le diagnostic de paludisme et un contrôle à 24 heures est réalisé si les symptômes persistent chez le patient. L'objectif de ce travail est d’évaluer la sensibilité de l'llumigen^®^ afin de confirmer son positionnement en test de dépistage du paludisme chez le patient tout-venant à l'hôpital.

## Matériels et méthodes

La technique de biologie moléculaire Illumigen^®^ Malaria repose sur le principe de *Loop-Mediated isothermal Amplification* (LAMP). Nous avons utilisé une technique de PCR en temps réel pour la détermination de nos résultats. Parmi les patients étudiés, 18 échantillons présentant un résultat positif en technique PCR en temps réel avec des Ct compris entre 30,16 et 40 ont été testés sur l'Illumigen^®^.

## Résultats

Tous les échantillons présentant des Ct inférieurs à 35 se sont révélés positifs sur l'Illumigen^®^, tandis que nous n'avons obtenu que 2 résultats positifs parmi les prélèvements présentant des Ct supérieurs à 35.

## Discussion

Ces résultats ont permis de mettre en évidence une bonne efficacité diagnostique de la technique Illumigen^®^ pour des Ct inférieurs à 35, qui correspond donc à la limite de détection de celle-ci. Tous les patients étudiés avaient reçu soit un traitement curatif avant l'analyse soit une chimioprophylaxie par doxycycline (avec observance incomplète), rendant la parasitémie négative au frottis sanguin. Ces résultats poussent donc à la prudence lorsqu'il s'agit d'un dépistage chez des patients ayant reçu une chimioprophylaxie: un contrôle à 24 heures apparaît alors nécessaire.

## Conclusion

Ce travail a permis de déterminer les limites de la technique Illumigen^®^, un défaut de sensibilité diagnostique chez des patients ayant été traités par antipaludéens. Néanmoins, ceci ne remet pas en cause le positionnement de cette analyse dans notre établissement pour le screening d'exclusion, car les patients que nous dépistons correspondent à des cas de paludisme d'importation, pour lesquels la notion de traitement préventif ou non est connu. Il est donc impératif pour le biologiste de disposer de l'ensemble des informations relatives au traitement et à la clinique du patient afin de pouvoir compléter la technique Illumigen^®^ par d'autres techniques complémentaires ou de réaliser un suivi rapproché du patient si nécessaire.

# Research article: Paludisme chez les sujets de plus de 100 kg et/ou en surpoids: étude monocentrique rétrospective

**Published:** 2023-03-02

**Authors:** Luc Labarbe, Christine Roves, Pierre-Louis Conan, Cécile Ficko

**Affiliations:** Service des maladies infectieuses et tropicales, Hôpital d'instruction des armées Bégin, Saint-Mandé, France

**Keywords:** Paludisme d'importation, Voyage, Surpoids, Thérapeutique

## Introduction

Les patients en surpoids sont à risque de sous-dosages médicamenteux et d’échecs thérapeutiques. Un échec thérapeutique a été ainsi rapporté chez un militaire de plus de 100 kilos, traité pour un accès palustre à *P. falciparum* par l'association arténimol-pipéraquine (4 cp sur 3 j), en lien avec un probable sous-dosage.

Cependant, aucune donnée dans la littérature n'est disponible pour adapter cette molécule au-delà de 100 kg. Les données concernant l'efficacité et la tolérance des antipaludéens chez les sujets en surpoids, obèses, et/ou pesant plus de 100 kg sont ainsi limitées.

## Matériel et méthodes

Étude descriptive monocentrique rétrospective de 2007 à 2019 de tous les cas de paludisme d'importation prouvés microbiologiquement ayant été pris en charge dans l'hôpital d'instruction des armées Bégin. L'objectif principal était de décrire l'efficacité de l'arténimol-pipéraquine chez les patients de plus de 100 kg. L'objectif secondaire était de décrire la tolérance des traitements antipaludiques chez les sujets en surpoids.

## Résultats

Quatre cent quarante-deux patients ont été inclus dans l'analyse, âge moyen 39 ans, 307/440 hommes (70%) dont 227/421 (54%) des patients sont en surpoids. Parmi les 29 patients de plus de 100 kg, la guérison était de 100%. L'arténimol-pipéraquine a été prescrit chez 7 patients et n’étaient pas associés à l’échec thérapeutique. La tolérance des antipaludiques était bonne chez les patients en surpoids (217/227 (96%) sans effet secondaire). Le principal effet secondaire observé sans différence significative dans les deux groupes est le vomissement lors de la prise d'atovaquone-proguanil observé dans 15 cas sur 420 dont 8 (3,5%) dans le groupe surpoids. Les autres effets secondaires sont un cas d'allergie à l'artésunate et un cas d'hépatite aiguë à la malarone. Le surpoids et l'obésité n’était pas associé au paludisme grave.

## Discussion

Les limites de notre travail sont sa conception rétrospective et monocentrique.

Contrairement à notre hypothèse initiale, l'arténimol-pipéraquine chez les sujets de plus de 100 kg n'a pas été associé à l’échec thérapeutique, peut-être par un manque de puissance causé par une rareté des échecs thérapeutiques et/ou par un effectif faible. L'absence de recueil des comorbidités est aussi une limite à la représentativité de notre étude.

## Conclusion

Il s'agit de la première étude rétrospective à comparer l'efficacité et la tolérance des antipaludiques respectivement chez les patients de plus de 100 kg et en surpoids.

D'autres études prospectives chez les sujets en surpoids sont nécessaires afin de proposer une thérapie « sur mesure » pour cette population.

# Research article: L'hésitation vaccinale et ses déterminants chez les parents d'enfants de moins de 5 ans dans la ville de Gao, au Mali en 2021

**Published:** 2023-03-02

**Authors:** Amadou Tila Kebe, Bakary Diarra, Aboubacar Sangho, René Migliani, Patrick Thonneau

**Affiliations:** Centre de santé de référence, Sankoré, Tombouctou, Mali

**Keywords:** Hésitation vaccinale, Refus de vaccination, Réticence à la vaccination, Vaccination, Mali

## Introduction

Depuis le début de la vaccination, des groupes s'y étaient opposés. Ce refus a persisté, quoique dans des groupes de personnes ou communautés peu nombreuses. Au cours des dernières décennies avec l'avènement d'Internet et des réseaux sociaux, les modes de communications se sont multipliés et le message s'est étendu à des territoires qui jusque-là acceptaient la vaccination. Vu l'ampleur de la situation, l'OMS a mis en place le Strategic Advisory Group of Experts (SAGE), qui a défini l'hésitation vaccinale par un « retard à l'acceptation d'un vaccin ou son refus, malgré une facilité d'accès ». L'objectif principal était d’étudier l'hésitation vaccinale chez les parents d'enfants de moins de 5 ans. Spécifiquement, il s'agissait de déterminer la prévalence de l'hésitation vaccinale et d’énumérer ses déterminants.

## Méthodologie

Il s'agit d'une étude transversale descriptive, réalisée de mars à août 2021 dans la ville de Gao, auprès de parents et gardiennes d'enfants de moins de 5 ans. Un sondage par quotas a permis de répartir l’échantillon minimum calculé entre les quartiers. Le recrutement des parents s'est fait sur la base de la méthode des itinéraires. Nous avons réalisé une enquête diagnostique et la technique utilisée était une enquête par entretien en face à face à l'aide d'un questionnaire.

## Résultats

Au total, 384 personnes ont répondu à notre enquête. Selon la définition du groupe de travail du SAGE, 9,11% des parents étaient « hésitants vaccinaux ». Parmi les répondants, 49% [31,38-66,01] ne pensaient pas que les vaccins soient sûrs et étaient préoccupés par les effets secondaires; 23% [10,42-40,14] hésitaient parce que quelqu'un d'autre les avait informés que les vaccins n’étaient pas sûrs et 17% [6,56-33,65] hésitaient parce qu'ils avaient entendu ou lu des avis négatifs (médias, entourage). Il existe une association statistiquement significative entre l'hésitation vaccinale des parents et le fait d'avoir moins de 30 ans, le fait de ne pas être scolarisé ou d'avoir un niveau de scolarisation bas, d'avoir moins de 3 enfants et d'avoir accès à Internet.

## Conclusion

L'hésitation vaccinale est une réalité dans la ville de Gao, en proie à l'occupation par des extrémistes religieux. L’étude nous a permis de comprendre le niveau du phénomène et d'identifier ses déterminants. En outre, elle a permis de fournir à la communauté scientifique des informations vérifiables sur le phénomène. Il serait idéal de mener une étude mixte à plus grande échelle pour comprendre le phénomène au Mali.

# Research article: Hésitation vaccinale à la pandémie de Covid-19: quelles leçons en tirer en Amérique du Nord, en Europe et en Afrique subsaharienne?

**Published:** 2023-03-02

**Authors:** Hadiatou Diallo

**Affiliations:** Université Sédar Senghor, Alexandrie, Égypte

**Keywords:** Hésitation vaccinale, Covid-19, Amérique du Nord, Europe, Afrique subsaharienne

## Introduction

La vaccination est l'une des interventions de santé publique les plus efficaces et efficientes qui a permis la réduction de la morbi-mortalité infanto-juvénile. Toutefois, son succès réside dans le niveau d'acceptation des vaccins par les communautés. Phénomène complexe, l'hésitation vaccinale fait partie des défis majeurs pour la vaccination et des grandes menaces pour la santé publique. Dans ce contexte de pandémie à Covid-19, obtenir l'adhésion des populations avec des niveaux faibles d'hésitation à la vaccination est indispensable pour garantir une immunité suffisamment importante pour lutter contre ce fléau. Ainsi, l'objectif de cette étude était de comprendre les déterminants de l'hésitation vaccinale dans les pays d'Amérique du Nord, d'Europe et de l'Afrique subsaharienne.

## Méthodes

Nous avons réalisé une revue systématique de la littérature sur l'hésitation à la vaccination Covid-19 dans les zones géographiques ciblées à travers une consultation de la base de données médicale et scientifique PubMed et en interrogeant la littérature grise. Cette recherche s'est faite du 8 au 9 juin 2021 à travers les mots-clés et en fonction des critères d'inclusion et d'exclusion constitués à partir des éléments PICO. La sélection, l'extraction et le traitement des données nous ont permis de retenir des publications conformément aux directives PRISMA.

## Résultats

Au total, 52 études ont été incluses dans cette revue dont 48 évaluées par les pairs, indexées sur PubMed, et 4 issues de la littérature grise. Après stratification, 76 enquêtes sur les taux d'hésitation/acceptation vaccinale ont été menées dans 32 pays. Les taux d'hésitation vaccinale ont varié en fonction des régions, des pays, du temps, et des propriétés des vaccins. Les deux extrêmes d'hésitation ont été enregistrés en Afrique (Cameroun 84,6% et Éthiopie 6%). Les préoccupations sur l'efficacité et l'innocuité des vaccins, la peur des effets secondaires, la perception du risque de maladie sont les principales raisons répertoriées dans notre revue. D'autres facteurs d'influence comme l'affiliation politique et l'appartenance raciale ont été notés en Amérique du Nord, les antécédents de vaccination contre la grippe en Europe et la méfiance sur l'existence de la maladie ainsi que les croyances conspirationnistes (les vaccins sont développés pour faire du mal aux Africains) ont été enregistrés en Afrique subsaharienne.

## Conclusion

Notre revue a permis d'analyser les niveaux d'hésitation vaccinale à la Covid-19 dans les zones géographiques ciblées, d'en comprendre les déterminants et de faire quelques propositions de solutions qui pourraient aider à lutter contre ce phénomène.

# Research article: Évaluation du système de surveillance de la lèpre, Niger, 2016 à 2018

**Published:** 2023-03-02

**Authors:** Zeinabou Alhassane Doudou, Fatoumata Mounkaila Issaka, Sani Karimou, Aboubacar Issiakou Gandou, Fati Sidikou, Issifou Djibo, Oumarou Batouré, Mariane Laurent, Joseph Otshudiandjeka, Fadima Diallo, Pauline Kiswendsida Yanogo, Nicolas Meda

**Affiliations:** West Africa Field Epidemiology and Laboratory Training Program, Ouagadougou, Burkina Faso

**Keywords:** Évaluation, Surveillance, Lèpre, Niger

## Introduction

La lèpre reste un véritable problème majeur de santé publique à travers le monde. Au Niger, 607 cas de lèpre ont été mis sous traitement en 2018 dont 279 cas ont été guéris de la lèpre. La détection des cas de lèpre est sensiblement stable ces dernières années.

L'objectif de notre étude était d’évaluer le système de surveillance de la lèpre au Niger du 1^er^ au 15 août 2019.

## Méthodes

Une étude descriptive transversale a été conduite. Tous les acteurs impliqués dans la surveillance, présents au moment de l'enquête et consentants ont été retenus pour l'entretien. Pour les attributs (critères de CDC) et l'utilité du système, l'analyse des quantitatifs s'est faite sous forme d'estimation de pourcentage. Les données de surveillance de la lèpre au Niger de 2016 à 2018 ont été analysées en termes de temps, lieu et personnes et présentées sous forme de tableaux, graphiques et textes. Des fréquences ont été calculées.

## Résultats

Au total, 51 agents impliqués dans la surveillance de la lèpre ont été interviewés. L'utilité du système et les attributs: simplicité, acceptabilité, représentativité, sensibilité, qualité des données, flexibilité/souplesse et réactivité ont été évaluées en fonction du niveau d'atteinte des indicateurs sous forme de pourcentage.

Pour l'attribut représentativité de 2016 à 2018, 984 cas de lèpre ont été enregistrés. Le sexe masculin représente 55,1% des cas de lèpre avec un sex-ratio H/F égal à 1,2. Les adultes constituent la tranche la plus touchée avec 96,2% des cas, dans les deux tranches la forme multi-bacillaire est la plus fréquente et représente 80% des cas de lèpre.

## Conclusion

Le système de surveillance de la lèpre au Niger est utile, simple, acceptable, représentatif, peu sensible et réactif, mais il n'est pas flexible.

# Research article: Réseau de biosûreté dans l'espace G5 Sahel au temps des épidémies et des urgences sanitaires de portée internationale

**Published:** 2023-03-02

**Authors:** Odile Ouwe Missi Oukem-Boyer, Bourèma Kouriba, Thérèse S. Kagoné, Mohamed A. Bollahi, Henry Fissou, Mohamed Sidi Laghdaf, Haoua Seini Sabo, Odilon Tanifor, Annette Bremer, Pina Knauf, Roman Woelfel

**Affiliations:** Fondation Mérieux Mali, Immeuble Saibou Sylla, Quartier Hamdallaye ACI 2000, Bamako, Mali

**Keywords:** Réseau, Biosûreté, G5 Sahel, Laboratoire mobile, Pathogènes émergents

## Introduction

La région sahélienne est régulièrement menacée par des épidémies récurrentes telles que la maladie à virus Ébola (Mali), la fièvre de la vallée du Rift (Mauritanie/Niger), la dengue (Burkina Faso/Mali), et l'hépatite E (Tchad/Niger). À l'instar des autres pays du monde, les membres du G5 Sahel (Burkina Faso, Mali, Mauritanie, Niger et Tchad) ont fait face à la pandémie de Covid-19 depuis les premiers cas importés apparus en mars 2020. Pour lutter contre ces risques épidémiques et contre un possible risque bioterroriste dans une région en proie à des menaces sécuritaires, un Réseau de biosûreté a été créé en novembre 2017, regroupant des institutions de recherche de la Mauritanie (INRSP), du Mali (CICM), du Niger (CERMES), du Burkina Faso (Centre Muraz) et le ministère de la Santé du Tchad. Il vise à renforcer la coopération entre les États membres du G5 Sahel en matière de biosûreté par le transfert de compétences pour le diagnostic d'agents hautement pathogènes afin d'améliorer la sécurité des populations. La coordination tournante de ce Réseau est actuellement assurée par le CERMES.

## Méthode

L'Institut de microbiologie de la Bundeswehr a doté le Réseau d'un laboratoire mobile de type EMlab et formé une équipe d'intervention rapide composée de scientifiques des 5 institutions membres du Réseau. Ce laboratoire mobile est hébergé par le Centre d'infectiologie Charles Mérieux du Mali. Le fonctionnement du Réseau s'appuie sur diverses activités parmi lesquelles: i) des ateliers de réflexion sur l'institutionnalisation du Réseau et le développement de procédures opérationnelles standards relatives au déploiement du laboratoire mobile; ii) des formations théoriques et pratiques de scientifiques au diagnostic des pathogènes émergents et à l'utilisation du laboratoire mobile, et iii) des exercices de déploiement du laboratoire mobile sur le terrain.

## Résultats

Une équipe de scientifiques a été formée dans chaque pays. Chaque institution a reçu des équipements permettant de réaliser le diagnostic de plusieurs pathogènes émergents tels que les virus Ébola, Marburg, Zika, Lassa, dengue, fièvre jaune et SARS-CoV-2. Les procédures relatives au déploiement du laboratoire mobile ont été développées et mises en œuvre. La demande d'institutionnalisation du Réseau auprès du Secrétariat exécutif du G5 Sahel a été soumise.

## Conclusion

La pandémie de Covid-19 a permis de réaliser que chaque pays avait besoin de disposer de son propre laboratoire mobile et d’équipes nationales d'intervention rapide. La reconnaissance officielle du Réseau de biosûreté par le Secrétariat exécutif du G5 Sahel, sa pérennité et son opérationnalité transfrontalière constituent des défis.

# Research article: Identification et suivi des personnes contacts à la Covid-19, à Tombouctou (Mali) en 2020

**Published:** 2023-03-02

**Authors:** Bocar Mahamane Traoré, Oumar Almouloud, Aissata Tandina, Dr Odile Clausse, Zinsou Rodrigue Ahodegnon, Patrick Thonneau

**Affiliations:** Université Sédar Senghor, Alexandrie, Égypte

**Keywords:** Covid-19, Cas contacts, Suivi, Tombouctou

## Introduction

La recherche des personnes contacts, leur isolement, un dépistage rigoureux et la prise en charge des cas constitue une stratégie clé pour interrompre les chaînes de transmission du SARS-CoV-2 et réduire la mortalité associée à la Covid-19. L'objectif de cette étude était de contrôler l'efficacité du suivi des personnes contacts à la Covid-19 à Tombouctou en 2020.

## Matériels et méthodes

Cette étude prospective s'est déroulée du 27 avril au 15 juillet 2020 dans le district sanitaire de Tombouctou. La population d’étude était constituée de toutes les personnes contacts auprès des cas positifs à la Covid-19 et identifiées *via* un processus mené par des agents de traçage. Ces personnes contacts ont été suivies pendant 2 semaines avec une visite biquotidienne par des agents de suivis (infirmiers/aides-soignants formés). La visite a permis de collecter les variables sociodémographiques ainsi que la température et de mener un interrogatoire clinique (test PCR réalisé si t° > 38,5 °C, toux, dyspnée, myalgie, céphalées). Le statut (vivant, décédé) a été notifié à la fin de l’étude. La confidentialité et l'anonymat des données ont été respectés.

## Résultats

Au total, 1 049 personnes contacts ont été investiguées avec un sex-ratio H/F de 1,2 et un âge moyen de 25 ± 16 ans (15 à 24 ans = 30%). Les élèves représentaient 40%, les ménagères 16%, les artisans 13% et les fonctionnaires 11%. Parmi les personnes contacts, on retrouve les membres de la famille (73%), les conjoint(e)s (10%) et les amis/proches (9%). Au niveau de Tombouctou, le lieu de résidence principal était le quartier Hamabangou (1/3) suivi du quartier d'Abaradjou (1/4). Les personnes contacts étaient en majorité des contacts étroits et prolongés de plus de 15 minutes avec le malade (92%). Sur les 1 049 personnes contacts, 632 ont été testées parmi lesquelles 69 (11%) sont revenues positives. Deux décès ont été enregistrés, soit 0,2% sur l'ensemble des personnes contacts (test PCR post-mortem positif).

## Conclusion

Notre étude a permis de mettre en évidence l'importance de la contamination des personnes contacts (1 personne contact positive sur 9 testées). Nous retrouvons aussi un risque de décès de 2 cas sur 1 000 relativement important. Ces résultats mettent en avant l'importance majeure d'identifier et de tester les personnes contacts pour une meilleure riposte.

# Research article: Apport du scanner dans le diagnostic des lésions séquellaires pulmonaires à Covid-19 à la Polyclinique Pasteur

**Published:** 2023-03-02

**Authors:** Mamoudou Camara, Abdoulaye Chomba Koné, Youssouf Koné, Cheick Oumar Touré, Moussa Konaté, Siaka Sidibé

**Affiliations:** CHU du Point G, Bamako, Mali

**Keywords:** Scanner, Lésions séquellaires, Poumon, Covid-19, Pasteur

## Introduction

La maladie provoquée par le virus nommé « SARS-CoV-2 » s'est rapidement propagée dans le monde et a été déclarée pandémie mondiale le 11 mars 2020. En République du Mali, le premier cas de Covid-19 a été confirmé le 25 mars 2020 et à la date du 27 octobre 2021, le Mali a cumulé plus de 15 879 cas confirmés pour 14 597 cas guéris, 559 décès et 723 cas sous traitement.

Le but de cette étude était de déterminer la fréquence des lésions séquellaires pulmonaires à Covid-19 au scanner à la Polyclinique Pasteur de Bamako et de décrire les aspects scanographiques des lésions séquellaires pulmonaires à Covid-19.

## Matériels et méthode

Il s'agit d'une étude prospective, descriptive, transversale sur les patients ayant réalisé un scanner thoracique initial et de contrôle à 6 mois après la guérison dans le service d'imagerie médicale de la clinique pendant la période d’étude de 12 mois, de janvier à décembre 2021.

## Résultats

Pendant la période d’étude, nous avons noté une fréquence d'apparition des lésions séquellaires à Covid-19 de 48,2%. La tranche d’âge de 40 à 60 ans était la plus représentée soit 51,7%, avec une moyenne de 47 ans. On a noté un sex-ratio de 2,2 en faveur des hommes. 72,4% de nos patients avaient un facteur de comorbidité avec une prédominance de l'association avec le diabète dans 32,7% des cas. La toux était le signe clinique le plus retrouvé soit 67,2%. Nous avons remarqué que 31% de nos patients avec toux ont développé des séquelles pulmonaires post-traitement Covid-19. 50% de nos patients avec séquelle pulmonaire post-traitement Covid-19 faisaient de la fièvre à l'admission. Le signe TDM initial le plus retrouvé dans notre étude a été les opacités en verre dépoli soit 62%. La fibrose pulmonaire a été retrouvée comme séquelle pulmonaire post-traitement Covid-19 la plus représentée durant notre période d’étude soit 35,7%, suivie des HTAP avec 17,8%.

## Conclusion

La pneumonie à Covid-19 est une nouvelle pandémie mondiale dont la présentation clinico-radiologique n'est pas spécifique. La TDM thoracique a une bonne sensibilité pour le diagnostic des lésions séquellaires pulmonaires à Covid-19.

# Research article: L'efficacité des tests moléculaires dans le diagnostic de la syphilis: revue systématique et méta-analyse

**Published:** 2023-03-02

**Authors:** Abibou Simporé, Valérie Bazié, Abdou Azaque Zouré, Abdoul Karim Ouattara, Rebeca Compaoré, Alice Kiba-koumaré, Yooda A. Paul, Florencia W. Djigma, Hermann Sombié, Cyrille Bisseye, Jacques Simporé

**Affiliations:** Laboratoire de biologie et de génétique moléculaire, Université Joseph Ki-Zerbo, Ouagadougou, Burkina Faso

**Keywords:** Tests moléculaires, Syphilis, Méta-analyse, PCR, Polymérase A, *tpp47*

## Contexte

La syphilis « the great imitator » continue d’être un problème de santé publique. Son diagnostic clinique et biologique comporte toujours des limites. Le diagnostic moléculaire constitue une alternative pour une prise en charge rapide et efficace.

## Objectif

Déterminer la précision des tests dans le diagnostic moléculaire de la syphilis chez l'homme.

## Stratégie de recherche documentaire et méthodologie

Nous avons recherché dans PubMed et Web of Sciences, des articles portant sur la détection moléculaire de la syphilis au cours de la période du 1^er^ janvier 2009 au 31 décembre 2019. L'outil QUADAS-2 (Quality Assessment of Diagnostic Accuracy Studies) a été utilisé pour évaluer la qualité des études et nous avons suivi les recommandations de la Collaboration Cochrane pour effectuer les analyses statistiques de type « Diagnostic Test Accuracy Review ». Le modèle bivarié de Reitsma et le modèle hiérarchique de courbe caractéristique de fonctionnement du récepteur (ROC: Receiver Operating Characteristic) ont permis d’évaluer les performances diagnostiques des tests moléculaires à un intervalle de confiance (IC) de 95%. Une méta-analyse en sous-groupe a été effectuée pour explorer les sources d'hétérogénéité.

## Résultats

47 articles ont été identifiés pour la synthèse qualitative, dont 26 répondaient aux critères d'inclusion de la méta-analyse de l’étude. Les sensibilités poolées dans la PCR conventionnelle et la PCR en temps réel étaient respectivement de 81,12% [66,43-90,32] et 62,38% [48,96-74,13]. Les spécificités poolées étaient de 97,83% [93,39-99,31] et 98,83% [92,22-99,83] respectivement. Les échantillons d'ulcères (lésions génitales ou autres) avaient un meilleur rendement (sensibilité = 81,67 [70,60-89,20] et spécificité = 98,23 [94,93-99,40] et les gènes cibles majoritaires étaient le gène de la *Polymérase A* et le gène *tpp47.*

## Conclusion

Notre travail a montré que la PCR conventionnelle était plus utilisée que la PCR en temps réel dans le diagnostic de la syphilis, et que les ulcères constituaient les meilleurs prélèvements. Les types d’échantillons ainsi que les gènes cibles sont autant de facteurs pouvant influencer la qualité des différents tests. Ces résultats pourraient fournir des preuves de travaux dans le sens de l'amélioration de proposer un test de diagnostic plus performant.

# Research article: Diversité génotypique de l'infection à papillomavirus humain chez les femmes au Cameroun: implications sur la stratégie vaccinale

**Published:** 2023-03-02

**Authors:** Michel Carlos Tommo Tchouaket

**Affiliations:** Centre international de référence Chantal Biya, 3077 Yaoundé-Messa, Cameroun

**Keywords:** HR-HPV, Taux de positivité, Génotypes, Vaccination, Cameroun

## Contexte

L'objectif de cette étude était de déterminer le taux de positivité et les facteurs de risque du papillomavirus humain à haut risque (HR-HPV) et de décrire la distribution des génotypes du HR-HPV au Cameroun.

## Méthodes

Une étude transversale a été menée chez les femmes consultant en routine entre juin 2020 et mai 2021 dans 2 hôpitaux de référence au Cameroun. La détection du HR-HPV a été réalisée par PCR en temps réel sur des écouvillons cervico-vaginaux. Un modèle de régression logistique a été utilisé pour identifier les facteurs indépendamment associés à la positivité du HR-HPV; avec p < 0,05 considéré comme statistiquement significatif.

## Résultats

Sur les 364 femmes inscrites, l’âge médian [intervalle interquartile, IQR] était de 41 [34-50] ans, 3,0% étaient fumeuses et 26,09% déclaraient avoir plus de 3 partenaires sexuels. Globalement, le taux de positivité au HR-HPV était de 21,43% (IC95%: 17,21-25,64). Selon le modèle de régression, les facteurs associés étaient le jeune âge (c'est-à-dire < 41 ans; ORa [95%CI]: 0,408 [0,194-0,862]; p = 0,018); le tabagisme (ORa [95%CI]: 5,199 [1,314-20,575]; p = 0,018) et le fait d'avoir plus de 3 partenaires sexuels ORa [95%CI]: 2,335 [1,133-4,811]; p = 0,022). Au total, 12 génotypes HR-HPV ont été identifiés: 18 (31,7%), 16 (20,6%), 39 (17,5%), 58 (14,3%), 66 (9,5%), 59 (7,9%), 35 (6,3%), 52 (6,3%), 33 (3,2%), 45 (3,2%), 56 (1,6%) et 68 (1,6%). Environ 22,22% des femmes étaient co-infectées par au moins 2 HR-HPV, dont une triple co-infection (HR-HPV 18, 39, 58). Conclusion. La positivité des HR-HPV au Cameroun semble actuellement faible et prédominante chez les jeunes, les fumeuses et les femmes ayant plus de trois partenaires. Cependant, la grande diversité génétique appelle des investigations approfondies afin d'adapter la stratégie de vaccination camerounaise aux génotypes circulants.

# Research article: Résistance d'Aedes *aegypti* aux insecticides au Niger: Première détection des mutations *kdr* V1016I, F1534C et V419L et leurs associations à la résistance aux pyréthrinoïdes

**Published:** 2023-03-02

**Authors:** Abdoul-Aziz Maiga, Aboubacar Sombié, Zéphirin Somda, Félix Yaméogo, Souleymane Iro, Jean Testa, Antoine Sanon, Ousmane Koita, Hirotaka Kanuka, David Weetman, Philip J. McCall, Athanase Badolo

**Affiliations:** Université Joseph Ki-Zerbo, URF-SVT, Laboratoire d'entomologie fondamentale et appliquée (LEFA), Ouagadougou, Burkina Faso

**Keywords:** *Aedes aegypti*, Résistance aux insecticides, Mutations *kdr*, Pyréthrinoïdes, Niger

## Introduction

*Aedes aegypti* est le vecteur de certaines arboviroses incluant la dengue, Zika, le chikungunya et la fièvre jaune. Dans un contexte de manque de vaccins efficaces et de médicaments spécifiques, la lutte antivectorielle reste le principal moyen de lutte contre ces maladies. Nous avons investigué le statut de résistance aux insecticides et les mécanismes impliqués au sein des populations d'Aedes *aegypti* de Niamey, en vue de fournir des données de base pour la prévention de la dengue qui est l'arbovirose la plus répandue en Afrique de l'Ouest.

## Méthodes

Les échantillons d'Aedes *aegypti* sont obtenus par utilisation des pondoirs pièges. Les œufs ont été éclos au laboratoire et suivis jusqu’à émergence. Les tests de bio-essai ont été réalisés sur les moustiques femelles de génération F1 et F2 afin d’évaluer leur sensibilité aux insecticides utilisés en santé publique. Les mutations *kdr* F1534C, V1016I et V419L ont été recherchés.

## Résultats

Les tests de bio-essai ont montré clairement que les populations urbaines d*’Aedes aegypti* de Niamey sont fortement résistantes aux pyréthrinoïdes mais restent sensibles aux organophosphates et aux carbamates. Les mutations *kdr* F1534C, V1016I et V419L ont été détectées et toutes ont été associées à la résistance aux perméthrines alors qu'uniquement F1534C a été associée à la résistance aux deltaméthrines.

## Conclusion

Nous rapportons pour la première fois des données sur le profil de sensibilité des populations d'Aedes *aegypti* de Niamey et les mécanismes associés, notamment les mutations *kdr*. Notre étude fournit des données substantielles pour le contrôle de la dengue et des autres arboviroses transmises par ce vecteur à Niamey, et donne aussi une idée sur le statut de résistance chez le vecteur dans le reste des zones urbaines du Niger.

# Research article: Recherche d'un réservoir de la fièvre Q dans la faune sauvage amazonienne: présentation préliminaire de l’étude Faunacox en Guyane

**Published:** 2023-03-02

**Authors:** Loïc Epelboin, Édith Guilloton, Mona Saoût, Magalie Demar, Roxane Schaub, Timothée Bonifay, Félix Djossou, Aurélie Couesnon, Élodie Rousset, Olivier Duron, Benoît De Thoisy

**Affiliations:** Unité des maladies infectieuses et tropicales et CIC INSERM 1424, Centre hospitalier de Cayenne Andrée-Rosemon, avenue des Flamboyants, 97300 Cayenne, Guyane, France

**Keywords:** Fièvre Q, *Coxiella burnetii*, Guyane, Amazonie, Zoonose

## Contexte

La fièvre Q, infection liée à la bactérie *Coxiella burnetii*, est une zoonose ubiquitaire de répartition mondiale. La Guyane, territoire français d'Amazonie, présente une situation hyperendémique avec le taux d'incidence annuel le plus élevé au monde, lié à une souche unique MST 117, considérée comme plus virulente que la souche européenne de référence. Les cas humains sont principalement concentrés sur Cayenne et ses environs, et un tel phénomène n'est pas retrouvé dans le reste de l'Amérique latine. Les professions à risque (vétérinaire, agriculteur, personnel d'abattoir) et le réservoir ovin et bovin habituel ne sont pas retrouvés, et le réservoir animal reste un mystère, bien que certains individus isolés d'espèces de mammifères sauvages aient été retrouvés porteurs de la bactérie: paresseux à trois doigts, cabiaï, suidés sauvages, notamment. Les études sur la fièvre Q dans la faune sauvage dans le reste de l'Amérique latine sont peu nombreuses et peu contributives. L'objectif principal de notre étude est donc la recherche du réservoir animal de *C. burnetii* dans la faune sauvage. L'un des objectifs secondaires de cette étude est de voir si une espèce en particulier représente le réservoir chez l'humain, comme cela a pu être affirmé pour le paresseux à trois doigts, ou bien si la bactérie circule au sein de nombreuses espèces de mammifères de façon anecdotique, sans qu'une espèce en particulier soit responsable de l’épidémiologie particulière retrouvée chez l'homme en Guyane.

## Méthodologie du projet

L’étude repose sur la recherche de *C. burnetii* par qPCR dans de multiples prélèvements d'animaux sauvages. Des collections de tissus provenant d’études antérieures sur d'autres zoonoses et conservés au sein de la collection Jaguars de l'association Kwata et de celle du laboratoire TBIP de l'Université de Guyane seront exploitées. De multiples prélèvements sont en cours de réalisation entre octobre 2021 et juillet 2022: fèces des mammifères du zoo en pleine nature de Guyane, collecte de fèces des animaux sauvages de 2 centres de sauvegarde, collecte de selles de félins (jaguar, puma) et d'herbivores sauvages (biche, tapir) auprès de l'Office français de la biodiversité, capture de chauves-souris avec recueil de fèces et écouvillons anaux et vaginaux en différents points de Cayenne où des épidémies humaines ont été rapportées, et campagne de récoltes de mammifères issus de la mortalité routière suite à un appel à contribution sur les réseaux sociaux. L’étape laboratoire de l’étude commencera à partir de mai 2022 avec extraction d'ADN suivie de réalisation de qPCR *Coxiella burnetii* sur les multiples prélèvements obtenus lors de la première phase.

## Résultats attendus

Les XXVII^e^ Actualités du Pharo seront l'occasion de présenter les résultats des qPCR *C. burnetii* réalisées sur des centaines d’échantillons de plusieurs dizaines d'espèces différentes de mammifères sauvages de la faune amazonienne.

# Research article: Manger sain, bouger plus, vivre mieux: une action d’éducation nutritionnelle auprès de la population égyptienne d'Alexandrie

**Published:** 2023-03-02

**Authors:** Odile Clausse, Rémy Mwanba Kalenga, Zinsou Rodrigue Ahodegnon, Mina Guirguis, Kadiatou Barry, Thierno Taibou Diallo, Patrick Thonneau

**Affiliations:** Université Sédar Senghor, 1 place Ahmed Orabi, Mancheya, BP 415, 21111 Alexandrie, Égypte

**Keywords:** Éducation nutritionnelle, Activité physique, Coopération internationale, Surpoids/obésité, Égypte

En Égypte, comme dans de nombreux pays de la ceinture méditerranéenne, le surpoids et l'obésité sont devenus un problème majeur de santé publique en quelques décennies et touchent particulièrement les femmes et les enfants. Si toutes les catégories d’âge sont concernées, les données récentes de la Banque mondiale alertent sur le poids de l'obésité chez les plus jeunes. Cette situation est notamment une conséquence d'une alimentation inadéquate et de l'inactivité physique.

Le département santé de l'Université Senghor à Alexandrie, en partenariat avec la faculté d’éducation sportive d'Abouqir de l'Université d'Alexandrie, a organisé une marche éducative afin de sensibiliser la population à l'alimentation saine et l'activité physique.

Cette communication a pour objectif de présenter un retour d'expérience sur l'organisation d'une action d’éducation nutritionnelle à destination du public en Égypte. Ce travail pourrait permettre d'améliorer la prise en compte des spécificités égyptiennes dans la préparation et la mise en œuvre de projets de santé publique en Égypte.

Après 6 semaines de préparation logistique (parcours, recherche de sponsors, autorisations) et technique (préparation de slogan, tracts et banderoles), cette marche a eu lieu le 18 mars 2022 le long de la Corniche à Alexandrie. Cet évènement a permis de réunir près de 350 participants et d'apporter une bonne visibilité des messages lors du rassemblement et tout au long du parcours. L’évènement a bénéficié d'une couverture médiatique à travers un reportage radio et télédiffusé. L’équipe a distribué environ 500 tracts aux passants et aux participants. L'ensemble de ces actions a permis de relayer des messages simples promouvant l'alimentation saine et l'activité physique. Une communication à la conférence internationale du 20 mars à la faculté d’éducation sportive a complété auprès des étudiants cette sensibilisation au problème de l'obésité chez l'adolescent en Égypte associant une alimentation inadaptée, une inactivité physique et une exposition croissante aux écrans.

Cette expérience a également présenté quelques écueils. Le projet initial devait comporter plusieurs points de chute intermédiaires sur le parcours avec des aménagements permettant des échanges plus aisés avec la population. Ils n'ont pas été autorisés par la sécurité.

Cette première édition de la marche pour la santé a permis de mener à bien une action de sensibilisation de la population d'Alexandrie à l'alimentation saine et à l'activité physique. Plutôt bien accueillie par la population, cette action réalisée en partenariat avec l'université d'Alexandrie pourrait être réitérée avec pour objectif de sensibiliser davantage les familles et les adolescents.

# Research article: Prévention des ingestions accidentelles de produits caustiques chez l'enfant dans les ateliers de teinturerie à Bamako au Mali: le cas de la commune VI

**Authors:** Mamadou Cisse, Issa Boubacar Maiga, Allaye Ombotimbe, Fatoumata Konaté, Abdoul Aziz Maiga, Moussa Bazongo, Mamadou Salo Koita, Soungalo Diop, Ibrahim Coulibaly, Adama Issa Koné, Sanibé Dramane Koné, Souleymane Coulibaly, Seydou Togo, Moussa Abdoulaye Ouattara, Sadio Yena

**Affiliations:** Service de chirurgie thoracique, CHU Hôpital du Mali, Faladié, Bamako, Mali

**Keywords:** Œsophage, Accident caustique, Teinture, Prévention

## Introduction

La survenue d'une ingestion accidentelle de produit caustique chez l'enfant constitue un drame social, économique et sanitaire.

## Objectif

Ce travail a pour but de contribuer à la réduction des risques d'ingestion accidentelle de produits caustiques (PC) chez l'enfant.

## Patients et méthode

Il s'agissait d'une étude prospective et descriptive qui s'est déroulée du 10 janvier au 31 mars 2021 dans la commune VI de Bamako au Mali. Elle décrit les conditions d'exercice des teinturiers qui exposent les enfants aux accidents d'ingestion de produits caustiques. Les données ont été recueillies par une enquête CAP sur la base d'un questionnaire qui a été testé avant sa mise en œuvre. Les données ont été ensuite enregistrées sur Excel 2016 puis par Epi Info avec p < 0,05. Le travail a été financé par un fond compétitif de l'ONG Solidarité Santé Navale.

## Résultats

Il y avait au total 458 teinturiers recensés avec une moyenne d’âge de 33 ans (14-75 ans) et un sex-ratio de 0,11. Dans 53,3% des cas, les teinturiers travaillaient seuls. Dans 80% des cas, ils n'avaient reçu aucune formation sur les dangers des PC. Dans 65% des cas, ils n'avaient aucune connaissance sur les dangers des PC sur l'humain, l'environnement et la nature. Dans 89,7% des cas, l'aménagement des sites n’était pas aux normes de sécurité et 85,8% des teinturiers exerçaient leur métier sans équipements de protection adéquats. Dans 56,5% des cas, les PC étaient conservés à domicile et les baignoires de teintures constituaient les récipients de conservation les plus fréquents avec 41,4%. Les enfants avaient accès aux sites de teinture dans 31% des cas et 9,2% des teinturiers affirmaient avoir recensé au moins un accident caustique sur leur site.

## Conclusion

La survenue des accidents d'ingestion de produits caustiques chez l'enfant est dû principalement à une ignorance et une négligence de la part de leurs utilisateurs, notamment dans le cadre de la teinturerie. Une politique de lutte axée sur l’éducation pour un changement de comportement de ces utilisateurs est une voie dans la prévention de ces accidents domestiques.

# Research article: Score prédictif d'infection urinaire chez les patients diabétiques hospitalisés à l'Hôpital principal de Dakar

**Published:** 2023-03-02

**Authors:** Moustapha Diop, Papa Samba Ba, Fatimata Wone, Daye Ka, Adja Ndèye Rokhaya Dione, Awa Ndao Fall, Mathilde Ndèye Sarr, Mouhamadou Ndiaye, Pape Moussa Diouf, Tracie Youbong, Abdou Khadre Fall, Abdourahmane Niang, Sara Boury Gning, Fatou Fall

**Affiliations:** Service des maladies infectieuses et tropicales, Hôpital principal, BP 3006, Dakar, Sénégal

**Keywords:** Infection urinaire, Diabète, Score prédictif, Dakar

## Introduction

L'infection urinaire (IU) chez les patients diabétiques peut être à l'origine de complications aiguës de diabète et engager ainsi le pronostic vital. L'objectif de notre étude était de proposer un score prédictif d'IU chez les patients diabétiques hospitalisés dans le département de médecine de l'Hôpital principal de Dakar (HPD).

## Matériels et méthode

Il s'agissait d'une étude rétrospective descriptive et à visée analytique allant du 1^er^ Janvier 2016 au 31 décembre 2019 portant sur les patients diabétiques hospitalisés au département de médecine de l'HPD. Après un échantillonnage aléatoire de la population d’étude en base d'apprentissage et base de test, nous avons créé un score prédictif de l'IU à partir d'un modèle de régression logistique multivariée. La courbe des caractéristiques de fonctionnement du récepteur (ROC) et son aire sous la courbe (AUC) ont permis de déterminer le pouvoir discriminant du score.

## Résultats

Nous avons colligé 573 patients diabétiques, majoritairement de type 2 (95%) avec une ancienneté moyenne du diabète de 11 ± 9 ans. Leur âge moyen était de 63 ± 14 ans et le sex-ratio était de 1. Un antécédent d'IU était présent chez 37 patients (6,5%). L'IU était diagnostiquée chez 157 patients (27%) avec comme principaux symptômes: la pollakiurie chez 34 (22%), la dysurie chez 24 (15%) et les brûlures mictionnelles chez 24 (15%). La base d'apprentissage était constituée de 372 patients (65%) et la base de test de 201 (35%). En analyse multivariée sur la base d'apprentissage, le sexe féminin (OR =1,9; IC95% [1,1-3,3]; p = 0,016), l'antécédent d'IU (OR = 3,7; IC95% [1,4-9,7]; p = 0,009), la neuropathie diabétique (OR =1,8; IC95% [1-3,2]; p = 0,04), la présence d'un signe urinaire (pollakiurie, dysurie ou brûlures mictionnelles) (OR = 6,9; IC95% [3,6-13,3]; p < 0,001), une CRP > 6 mg/L (OR = 2,9; IC95% [1,5-5,7]; p = 0,002) et l'anémie (OR = 1,8; IC95% [1-3,2]; p = 0,04) étaient indépendamment associés à la présence d'une IU. L’équation score est: Score_IU = -3,3 + 0,65*sexe (féminin) + 0,18*(Âge > 65 ans) + 1,3*Antécédent d'IU + 0,6*neuropathie diabétique + 1,9*signe urinaire + 1,1*(CRP > 6 mg/L) + 0,6*Anémie. Les AUC étaient de 78%, 72% et 76% sur les groupes d'apprentissage, de test et sur la population totale, respectivement.

## Conclusion

L'IU reste fréquente chez les patients diabétiques, un bon score de prédiction pourrait aider à son diagnostic précoce et à prévenir les complications aiguës du diabète.

# Research article: Étude des facteurs associés aux décès chez les patients atteints de tuberculose suivis dans deux centres de prise en charge à Dakar

**Published:** 2023-03-02

**Authors:** Moustapha Diop, Papa Samba Ba, Jean Augustin Diegane Tine, Ndèye Maguette Fall, Tracie Youbong, Mouhamadou Ndiaye, Ndèye Mouminatou Mbaye, Mouhamed Bouye Nestor Diatta, Alassane Sarr, Sokhna Walo Ndiaye, Ibrahima Cisse, Louise Fortes Déguénonvo, Abdourahmane Niang, Adama Faye, Moussa Seydi

**Affiliations:** Service des maladies infectieuses et tropicales, Hôpital principal, BP 3006, Dakar, Sénégal

**Keywords:** Tuberculose, Décès, Facteurs associés, Dakar

## Introduction

La tuberculose représente la première cause de décès d'origine infectieuse seule dans le monde. Les facteurs prédictifs de décès lié à la tuberculose au Sénégal sont rarement évalués. L'objectif de cette étude était d'identifier les facteurs associés aux décès chez les patients atteints de tuberculose suivis dans deux centres de prise en charge à Dakar (Sénégal).

## Matériels et méthode

Il s'agit d'une étude prospective descriptive et analytique portant sur les patients atteints de tuberculose suivis dans les services des maladies infectieuses et tropicales (SMIT) du Centre hospitalier national universitaire de Fann et de l'Hôpital principal de Dakar (HPD) et dans le service de pneumologie de l'HPD du 1^er^ mars 2019 au 29 février 2020. La régression logistique multivariée a été réalisée pour identifier les facteurs associés au décès avec un seuil de significativité statistique de 0,05.

## Résultats

Deux cent quatre-vingt-deux (282) patients hospitalisés au SMIT de Fann (57%), au SMIT de HPD (31%) et en pneumologie de HPD (12%) avaient été inclus. Leur âge moyen était de 39 ± 16 ans et le sex-ratio était de 2,2. Les patients étaient majoritairement mariés (50,7%), scolarisés (72%) et leurs niveaux d'instruction étaient primaire, secondaire, universitaire respectivement chez 28,1%; 43,8% et 28,1% des cas. Les patients tabagiques représentaient 23% des cas. Une notion de contage tuberculeux était retrouvée chez 25,9% des cas et 8.5% avaient une tuberculose antérieure. L'infection à VIH était présente chez 28,4% des cas. La majorité des patients étaient de nouveaux cas de tuberculose (91,1%). Les présentations cliniques étaient constituées de 33,3% de formes pulmonaires seules; 30.5% de formes extrapulmonaires seules; et 36,2% de formes mixtes. Les localisations extrapulmonaires qui prédominaient étaient ganglionnaire (36,1%) et pleurale (11,7%). La durée médiane d'hospitalisation était de 15 jours avec des extrêmes de 1 et 73 jours. Vingt-deux (22) patients étaient décédés, soit un taux de létalité de 7,8%. En analyse multivariée, les facteurs indépendamment associés au décès étaient l’âge ≥ 60 ans (OR = 26,2 IC95% [3,6-191,2] comparé à la tranche d’âge 16-40 ans), la présence d'une infection à VIH (OR = 7,23 IC95%[1,41-36,96]), la localisation neurologique de la tuberculose (OR = 13,19 IC95%[3,2-54,32]) et un taux d'hémoglobine < 10 g/dl (OR = 5,52 IC95% [1,28-23,86]).

## Conclusion

La tuberculose reste une affection mortelle malgré les avancées thérapeutiques. Une meilleure connaissance des facteurs associés aux décès liés à la tuberculose pourrait permettre de réduire sa létalité.

# Research article: Caractérisation exhaustive du venin de deux serpents venimeux au Maroc: *Daboia mauritanica* et *Cerastes cerastes*

**Published:** 2023-03-02

**Authors:** Soukaina Khourcha, Ines Hilal, Mohamed Chablaoui, Amal Safi, Abdelaziz Hmyene, Naoual Oukkache

**Affiliations:** Laboratoire des venins et toxines, Institut Pasteur du Maroc, 1 place Louis Pasteur, 20250 Casablanca, Maroc

**Keywords:** *Cerastes cerastes*, *Daboia mauritanica*, Venin de serpent, Caractérisation toxicologique, Activités biologiques, Réaction croisée

Les accidents d'envenimation ophidienne constituent un grave problème de santé publique dans de nombreux pays du monde. Plus de 5 millions de cas d'accidents de ce type se produisent chaque année, causant plus de 100 000 décès. En Afrique, plus de 20 000 décès par an sont enregistrés tandis que 400 000 victimes d'envenimations conservent des séquelles fonctionnelles graves et permanentes. Au Maroc, les morsures de serpent sont fréquentes et d'une plus grande gravité chez les enfants. Ils se produisent principalement dans les zones rurales. L'incidence de ces morsures reste mal connue et largement sous-estimée. Les données épidémiologiques sont mal connues du fait de l'absence de registre national, alors qu'une partie importante des envenimations ne bénéficie que des modes de traitement traditionnels en réanimation non médicale. Cela nous a incités à étudier les propriétés enzymatiques et biologiques des constituants biochimiques du venin de deux des venins de serpent les plus dangereux au Maroc: *Cerastes cerastes* (Cc) et *Daboia mauritanica (Dm).* De plus, nous avons étudié la réactivité croisée immunitaire des venins *Cc* et *Mm* par rapport à celle d'une autre vipère marocaine dangereuse, *Bitis arietans (Ba)*, afin d'identifier les meilleurs candidats (venin ou mélange de venins) pour produire le plus efficace et protecteur des anti-venins. Dans la présente étude, nous rapportons une caractérisation préliminaire du venin de *Cc* et *Dm* et la réactivité croisée qui peut exister entre leurs venins et le venin *Ba.* Ces venins sont connus pour être hautement toxiques et contiennent plusieurs protéines qui diffèrent par leur poids moléculaire. Fait intéressant, les venins *Cc* et *Dm* sont caractérisés par des activités hémorragiques et phospholipase A_2_ intenses et leur capacité à dégrader les chaînes α et γ du fibrinogène. Ils affichent une protéolyse très faible grâce au test à la caséine. Après injection chez la souris, *Cc* et *Dm* induisent une myonécrose des muscles squelettiques, ce qui reflète très probablement l'action directe des myotoxines et l'action indirecte des molécules hémorragiques présentes dans ces venins. Chez la souris, cette myonécrose diminue les taux sériques de créatine phosphokinase (CPK). Comme prévu, le venin *Cc* est immunogène et avec un anti-venin hautement protecteur contre les antigènes de venin *Dm* et *Ba.* Cette capacité protectrice est similaire à celle de l'antivenin produit contre le venin de *Dm*.

# Research article: Premier cas d'angiostrongylose abdominale à *Angiostrongylus costaricensis* en Amazonie française

**Published:** 2023-03-02

**Authors:** Anissa Desmoulin, Alessia Melzani, Céline Dard, Mathieu Nacher, Félix Djossou, Mohamed Kinan Drak Alsibai, Loïc Epelboin

**Affiliations:** CHU de la Réunion, allée des Topazes, 97400 La Réunion, France

**Keywords:** *Angiostrongylus costaricensis*, Guyane française, Voyageur, Parasitose intestinale, Rapport de cas

L'angiostrongylose abdominale est une maladie parasitaire causée par *Angiostrongylus costaricensis* au stade larvaire (L3) dont le premier cas a été décrit au Costa Rica par Pedro Morera et Rodolfo Cespedes en 1971. Elle a ensuite été rapportée du Texas au sud de l'Argentine, y compris les régions amazoniennes et les Caraïbes. Rares sont les cas identifiés en Amazonie, et aucun cas n'a été notifié sur le Bouclier des Guyanes.

Nous présentons le cas d'un homme de 34 ans qui a vécu pendant 7 ans en Guyane française et séjourné entre 2018 et 2020 en Guadeloupe (10 jours), Martinique (3 jours) et île de Tobago (21 jours). Il faisait fréquemment des randonnées dans la forêt amazonienne et consommait souvent du poisson cru pêché en rivière. Il a consulté en mai 2020 à Cayenne pour des douleurs abdominales, des diarrhées récurrentes et des céphalées depuis 15 jours. L'hémogramme a montré une leucocytose avec une éosinophilie à 10,9 G/L (54,8% des leucocytes totaux) et une cytolyse hépatique avec ALAT 162 UI/L et ASAT 84 UI/L. Le sérodiagnostic de *Taenia solium, Fasciola hepatica, Schistosoma mansoni, Toxocara canis, Trichinella spiralis, Gnathostoma* spp *et Strongyloides* spp était négatif par ELISA. Les tests pour les filaires et *Ascaris lumbricoides* étaient positifs par ELISA. Le sérodiagnostic *dEchinoccoccus* spp était positif par ELISA mais non confirmé par le Western blot. La sérologie *Angiostrongylus* spp était revenue positive par ELISA et confirmée par le Western blot. Une coloscopie a été réalisée et l'histopathologie du côlon a montré un infiltrat éosinophile avec des œufs et un agent intravasculaire allongé très évocateur d'angiostrongylose abdominale. Le patient a été traité par albendazole 400 mg une fois par jour pendant 14 jours et une dose unique d'ivermectine 200 µg/kg. Après un an de suivi, la symptomatologie s’était amendée et l’éosinophilie avait diminué à 1,5 G/L (14,8%).

Il s'agit du premier cas décrit dans la partie orientale de l'Amazonie. Quelques cas ont été rapportés dans les régions amazoniennes de la Colombie, de l’Équateur et du Pérou. Dans le cas présent, le lieu de contamination de ce voyageur français est probablement la Guyane française ou la Guadeloupe, mais la Martinique et Tobago ne sont pas à exclure. Les hôtes définitifs, *Rattus rattus* et *Rattus norvegicus* sont présents sur le Bouclier des Guyanes rendant possible la transmission humaine. Des études complémentaires sont nécessaires pour déterminer la présence d’*A. costaricensis* chez les rongeurs du Bouclier des Guyanes.

# Research article: Facteurs associés à la méningite bactérienne aiguë avant et après l'introduction du vaccin conjugué A en 2017 au Mali, Bamako, 2021

**Published:** 2023-03-02

**Authors:** Toumani Sidibé, Oumar Sangho, Djibril Barry, Assetou Dembele, Souleymane Coulibaly, El Hadj Issa Amaguiré Sy, Jean Kabore, Pauline Yanogo, Nicolas Meda

**Affiliations:** Direction générale de la Santé et de l'hygiène publique, Bamako, Mali

**Keywords:** Facteurs, Méningites bactériennes aiguës, Vaccins, Mali

## Introduction

La méningite bactérienne aiguë constitue un problème de santé publique, du fait de son potentiel épidémique malgré les stratégies de vaccination adoptées. Le but était d’étudier le profil épidémiologique et les facteurs associés aux méningites bactériennes aiguës avant et après l'introduction du vaccin conjugué A au Mali.

## Méthodes

Il s'agit une étude transversale à visée analytique. Tous les prélèvements reçus au Laboratoire national de référence pour suspicion de méningite de 2014 à 2019 ont été inclus dans cette étude.

## Résultats

Nous avons enregistré 646 patients atteints de méningite bactérienne aiguë dont 330 avant et 316 après l'introduction du vaccin conjugué A. Les enfants de moins d'un an étaient les plus touchés. Le *S. pneumoniae* était le plus souvent responsable: 186 (56%) des méningites bactériennes aiguës avant contre 176 (56%) après l'introduction du vaccin conjugué chez les enfants de moins d'un an. L'introduction du vaccin conjugué A a entraîné la disparition du méningocoque A. Cependant d'autres souches de méningocoques – *Neisseria meningitidis* C, X et W135 – restent le plus responsables de la méningite bactérienne aiguë chez les enfants de plus de 5 ans. Les tranches d’âge de moins d'un an (p < 0,001), 5-14 ans, 14-29 ans (p < 0,001), les personnes vivant en milieu rural (p < 0,001) et l'aspect trouble du LCR (p < 0,001) étaient indépendamment associés à la méningite bactérienne aiguë.

## Conclusion

La méningite bactérienne aiguë reste un problème de santé publique. Une bonne couverture vaccinale demeure la seule solution pour l’élimination de la méningite en tenant compte des changements des sérotypes à l'origine des épidémies.

# Research article: Toxoplasmoses « amazoniennes » hospitalisées ou vues en consultation en Guyane française de l'adulte immunocompétent: étude de cohorte rétrospective de 2002 à 2019

**Published:** 2023-03-02

**Authors:** Nolwenn Vidal, Jean-François Faucher, Romain Blaizot, Audrey Valdes, Vincent Pommier De Santi, Aurélien Mercier, Raymonde Taibi, Marie-Laure Dardé, Magalie Demar

**Affiliations:** CHU Limoges, INSERM U1094, Institut de Neuroépidémiologie tropicale, GEIST, Limoges, France

**Keywords:** Toxoplasmose amazonienne, Immunocompétent, Hôpital, Guyane française, Cohorte

## Introduction

La toxoplasmose « amazonienne » est connue depuis les années 1990 pour ses tableaux cliniques atypiques et en particulier sa sévérité chez le patient immunocompétent. L'objectif de ce travail est de faire la synthèse des cas de toxoplasmose acquise en Guyane française chez l'adulte immunocompétent.

## Matériels et méthodes

Cetravail rétrospectif (2002-2019) a inclus les patients non immunodéprimés et âgés d'au moins 18 ans. Les femmes enceintes asymptomatiques avec une séroconversion ont été exclues. Le diagnostic de toxoplasmose était retenu sur une clinique compatible associée à une PCR toxoplasmose positive ou une séroconversion objectivée ou une sérologie avec IgM positives.

## Résultats

Sur un recueil de 174 patients, le sex-ratio était de 1,76 avec une moyenne d’âge de 35,8 ans chez des patients peu comorbides. Les formes pauci-symptomatiques représentaient 42,5% (74/174) avec une atteinte ganglionnaire fébrile prédominante. L'atteinte pulmonaire associée ou non au syndrome de détresse respiratoire aiguë est significativement plus importante chez les patients qui présentent une forme sévère que ceux qui présentent des formes pauci-symptomatiques (p < 0,0001). En analyse multivariée, l'ethnie amérindienne est un facteur protecteur contre les formes graves (p 0,017 OR 0,011 [0-0,438]). L'analyse des souches génotypées montre une homogénéité des souches malgré leur grande diversité à l'intérieur d'un pool génétique commun. Aucun facteur pronostique en lien avec la génétique des souches n'a pu être mis en évidence.

## Conclusion

Ce travail fait ressortir la part non négligeable des formes pauci-symptomatiques dans la toxoplasmose « amazonienne », jusque-là peu décrites en dehors des atteintes oculaires isolées. La diversité génétique des souches de toxoplasmose « amazonienne » n'explique pas à elle-seule l'atypie des tableaux, et les facteurs de l'hôte y participent. Les Amérindiens semblent être protégés vis-à-vis des formes graves, sans pouvoir déterminer la part du degré de l'exposition particulier à leur mode de vie ou celle du patrimoine génétique en lien avec une coévolution avec les souches « amazoniennes » pour expliquer cette protection.

# Research article: Profil épidémiologique des leishmanioses cutanées prises en charge à Tlemcen, Algérie

**Published:** 2023-03-02

**Authors:** Samira Benbekhti Abdrebbi, Kaouel Meguenni

**Affiliations:** Faculté de médecine, Université Abou Bekr Belkaid, Tlemcen, Algérie

**Keywords:** Leishmaniose cutanée, Épidémiologie, Endémie, Habitat, Tlemcen

## Introduction

L'Algérie est considérée comme un pays à forte charge de leishmaniose cutanée. Elle est devenue une maladie à déclaration obligatoire depuis 1990. Son mode d'expression est endémo-épidémique. L'objectif de l’étude était de décrire le profil épidémiologique des leishmanioses cutanées (LC) prises en charge à Tlemcen.

## Matériel et méthodes

L’étude descriptive à recueil prospectif sur 5 ans, à partir de l'année 2012 jusqu’à 2016 sur la base de la déclaration systématique des cas de LC parvenue des différents services du Centre hospitalo-universitaire (CHU) de Tlemcen. La saisie et l'analyse des données ont été effectuées par le logiciel Epi Info 6.

## Résultats

Trente-quatre (34) cas de leishmaniose cutanée, dont 27 cas autochtones ont été rapportés au CHU de Tlemcen pendant 5 ans avec une prédominance féminine; sex-ratio (H/F): 0,79. La tranche d’âge la plus touchée était celle de 40-50 ans. Six (6) cas ont survenu chez des enfants de moins de 16 ans. 80% des déclarations ont été faites par le service de dermatologie. Le pic le plus élevé a été enregistré en 2013; une prédominance automnale était notée dans 30% des cas. Tous les patients vivaient ou avaient vécu dans des zones endémiques.

## Discussion

Les deux premiers cas autochtones ont été signalés en 1986 dans la ville de Tlemcen. La LC s'est étendue à Tlemcen et depuis, cette parasitose a été ajoutée à la carte de répartition géographique en Algérie.

La leishmaniose cutanée représente 90% des maladies à transmission vectorielle signalées par les services de santé. En Algérie, tous les âges sont concernés du nourrisson au vieillard, les enfants de moins de 5 ans représentant 27,4%. L'incidence la plus élevée a été observée en automne et au début de l'hiver.

# Research article: Épidémiologie de la fièvre boutonneuse méditerranéenne à Tlemcen, Algérie

**Published:** 2023-03-02

**Authors:** Samira Benbekhti Abdrebbi, Kaouel Meguenni

**Affiliations:** Faculté de médecine, Université Abou Bekr Belkaid, Tlemcen, Algérie

**Keywords:** Fièvre boutonneuse méditerranéenne, Zoonose, Prévention, Lutte, Tlemcen

## Introduction

La fièvre boutonneuse méditerranéenne (FBM) est l'une des plus anciennes maladies infectieuses vectorisées décrites. C'est une zoonose urbaine et péri-urbaine endémique sur le pourtour méditerranéen et en Afrique. Dans la région méditerranéenne, l'incidence de la FBM est estimée à environ 50 cas pour 100 000 habitants.

L'objectif de notre étude était de décrire le profil épidémiologique de la fièvre boutonneuse méditerranéenne à Tlemcen.

## Matériel et méthodes

Entre 2001 et 2018, une étude prospective a été réalisée à partir des déclarations faites par les différents services du CHU de Tlemcen. La saisie et l'analyse des données ont été effectuées à l'aide du logiciel SPSS 17.

## Résultats

Sur une période de 18 ans, 257 cas de FBM ont été notifiés avec une nette prédominance masculine, soit 76,3% des cas, le sex-ratio s’établissant à 3,21. La moyenne d’âge des patients est de 46,04 ans, l'adulte âgé de plus de 50 ans étant le plus touché (45% des cas). Les cas pédiatriques représentent 4% des cas. L'incidence annuelle de la FBM est en hausse durant la décennie 2011-2018 par rapport à celle de 2001-2010 avec un passage du nombre annuel moyen de 11 à 18 cas. La FBM sévit durant la période chaude entre les mois de mai à octobre, avec un pic entre mai et août.

## Discussion

À Tlemcen comme en Algérie, très peu d’études épidémiologiques ont été menées sur la fièvre boutonneuse méditerranéenne. Les résultats retrouvés concordent avec la littérature: la différence entre les sexes peut être liée à l'exposition professionnelle ou une susceptibilité spécifique masculine. La FBM est une maladie saisonnière à caractère estival. Le chien reste le principal réservoir chez les patients.

## Conclusion

L’épidémiologie de la fièvre boutonneuse méditerranéenne est peu connue dans la wilaya de Tlemcen. Le nombre réel de cette pathologie est sous-estimée car elle est sous-déclarée.

La présente étude permet de mieux connaître l’épidémiologie de la FBM qui reste préoccupante et endémique dans la wilaya de Tlemcen par ses tendances croissantes depuis ces dernières années, d'où la nécessité de mettre en place une stratégie de lutte basée essentiellement sur la lutte antivectorielle et la prévention.

# Research article: Cancer du col de l'utérus en milieu tropical: prise en charge au service d'oncologie médicale du CHME Le Luxembourg (Bamako), à propos de 200 cas

**Published:** 2023-03-02

**Authors:** Madani Ly, Kalilou Madani Ouattara, Cheick Mansour Diarra, Mahamadou Coulibaly, Zakari Saye, Abdoul Karim Konaté, Kalifala Samake, Aphou Salle Koné, Ramata Ly, Yeyia Djiré, Safiatou Kalle

**Affiliations:** Service d'oncologie médicale du Centre hospitalier mère-enfant Le Luxembourg, Bamako, Mali

**Keywords:** Cancer, Col de l'utérus, Radiothérapie, Chimiothérapie, Mali

## Introduction

Le cancer du col utérin représente 10% des cancers féminins. L'infection à HPV est le facteur de risque principal avec une prévalence de 21% à 35% en Afrique.

Le diagnostic est le plus souvent tardif. Malgré l’évolution des moyens diagnostiques et thérapeutiques, la vaccination efficace contre l'HPV, la prise en charge demeure un véritable défi en milieu tropical. C'est ainsi que nous avons mené cette étude dans le but d’évaluer la prise en charge des cancers du col dans le service d'oncologie médicale du CHME « Le Luxembourg » de Bamako.

## Méthodologie

Notre étude a été réalisée dans le service d'oncologie médicale du CHME Le Luxembourg du 1^er^ janvier 2017 au 31 décembre 2020. Il s'agissait d'une étude descriptive à collecte de données rétrospectives de 2017 à 2019 et prospectives en 2020, soit une période de 4 ans.

Au total, nous avons colligé 199 patientes ayant un cancer du col de l'utérus confirmé par la cytologie et/ou l'histologie.

## Résultats

Pendant la période d’étude, nous avons enregistré 2 750 nouveaux patients atteints de cancer dont 1 673 de sexe féminin. Parmi ces patientes, 199 ont présenté le cancer du col de l'utérus, soit une fréquence de 11,89%. L'atteinte de la totalité des paramètres a été retrouvée chez 25,1% des patientes, 20,6% pour les 2/3 des paramètres et 10,6% pour 1/3.

L'histologie a été dominée par les carcinomes épidermoïdes, 92% et 49% étant moyennement différenciés. À leur admission au service, 55% étaient au stade IV de la maladie.

Plusieurs techniques chirurgicales ont été utilisées pour seulement 21,1% des patientes; la CHEL avec 1,5%, l'hystérectomie avec 18,1%, la myomectomie avec 0,5%, la tumorectomie du vagin avec 0,5% et la pose de sonde JJ avec 0,5%. La radiothérapie a été pratiquée chez seulement 16 patientes soit 8%. La chimiothérapie a été pratiquée chez 152 patientes soit 76%; 72,9% ont eu la première ligne, 10,1% la deuxième ligne et 0,5% la troisième ligne. Le protocole paclitaxel-carboplatine a été le plus administré avec 75,4%. Au terme de cette prise en charge, 4,5% des patientes ont eu une rémission complète et 173 patientes soit 87% sont décédées.

## Conclusion

Le cancer du col de l'utérus est un cancer de la femme jeune diagnostiqué le plus souvent au stade métastatique. L'histologie était dominée par les carcinomes épidermoïdes. Le traitement précoce et multimodal permet une survie meilleure des patientes.

# Research article: Dépistage moléculaire de l'infection à *Anaplasma marginale* chez des bovins répartis du nord au sud de la Tunisie et évaluation des facteurs de risque associés

**Published:** 2023-03-02

**Authors:** Meriem Ben Abdallah, Hanène Belkahia, Rachid Selmi, surname> Romdhane, Moez M'hadhbi, Mohamed Aziz Darghouth, Mourad Ben Said, Lilia Messadi

**Affiliations:** Service de microbiologie et immunologie, École nationale de médecine vétérinaire, Université de la Manouba, Sidi Thabet, Tunisie.

**Keywords:** Dépistage moléculaire, *Anaplasma marginale*, Bovins, Facteurs de risque, Tunisie

L'anaplasmose bovine est une maladie à transmission vectorielle due essentiellement à l'infection des érythrocytes par l'espèce *Anaplasma marginale.* Cette maladie est largement répandue dans les pays tropicaux, subtropicaux et tempérés du monde. La forme aiguë de la maladie se traduit par un syndrome fébrile, une anémie hémolytique sans hémoglobinurie (anémie extravasculaire), une dyspnée, une tachycardie, et une hypotonie du rumen.

La production laitière chute de façon foudroyante et les femelles gestantes peuvent avorter suite à l'anoxie fœtale. Les animaux qui survivent à l'infection aiguë développent une infection persistante caractérisée par une bactériémie cyclique et deviennent des non-valeurs économiques.

Dans ce contexte, la présente étude a eu pour objectif d'estimer la prévalence moléculaire de l'infection à *A. marginale* chez 545 bovins asymptomatiques, appartenant à 89 élevages répartis dans 11 gouvernorats de la Tunisie (Bizerte, Ariana, Manouba, Siliana, Jendouba, Béja, Kairouan, Kasserine, Zaghouan, Sousse, Gabès) et 8 zones bioclimatiques (subhumide, humide supérieur, humide inférieur, semi-aride supérieur, semi-aride moyen, semi-aride inférieur, aride supérieur, aride inférieur), et de déterminer les facteurs de risque associés. Une PCR spécifique du gène *Msp4* a permis de confirmer l'infection de 14 bovins par *A. marginale* soit une prévalence globale de 2,57% (14/545). Une analyse statistique a été réalisée afin de comparer la prévalence de l'infection par l'espèce *A. marginale* chez les bovins étudiés, en fonction des gouvernorats, des délégations, des étages bioclimatiques, de la race, du sexe, de l’âge et de l'infestation par les tiques.

Il s'est avéré que les bovins appartenant à l’étage subhumide étaient statistiquement les plus infectés par *A. marginale*. Cette constatation est probablement corrélée à l'effet des conditions bioclimatiques sur la phénologie et la distribution des vecteurs notamment les tiques.

# Research article: Leishmaniose cutanée: un exanthème maculopapuleux généralisé induit par le glucantime. Lorsque la pharmacovigilance ne doit pas se relâcher

**Published:** 2023-03-02

**Authors:** Omar Boudghene Stambouli

**Affiliations:** Service de dermatologie, Faculté de médecine, Laboratoire Toxi Med 32, Université Abou Bekr Belkaid, Tlemcen, Algérie

**Keywords:** Exanthème maculopapuleux, Glucantime, Leishmaniose cutanée

## Introduction

Les médicaments les plus souvent incriminés dans les réactions cutanées sont l'allopurinol, l'aminopénicilline, les céphalosporines, les antiépileptiques et les sulfamides antibactériens. Les toxidermies correspondent aux effets secondaires médicamenteux à expression cutanéomuqueuse. La réaction cutanée induite par le glucantime est rarement signalée et nous rapportons un exanthème maculopapuleux dû à la prise de glucantime par voie générale.

## Observations

Patient âgé de 15 ans sans antécédent pathologique admis au service de dermatologie le 23 mars 2017 pour prise en charge d'une leishmaniose cutanée siégeant dans la partie antérieure de la main. Le patient avait reçu 10 infiltrations de glucantime mais sans amélioration. Devant la persistance de la lésion et la résistance au traitement local, on préconise un traitement par glucantime par voie générale à la dose de 70 mg/kg/j soit 12 ml/j.

Après un bilan pré-thérapeutique, le traitement a été instauré à une dose progressive (1/4 de la dose totale puis 1/2 puis 3/4 et la dose totale à J4). Au 7^e^ jour de traitement, il a développé une éruption cutanée prurigineuse au niveau des faces antérieures et internes des avant-bras ainsi qu'au niveau de la partie supérieure du dos évoluant dans un contexte d'apyrexie. À l'examen, il avait un visage rouge, une éruption érythémato-papuleuse associée à des lésions de grattage au niveau des faces antérieures et internes des avant-bras, au niveau de la partie supérieure du dos et au niveau du tiers inférieur des jambes sans atteinte des muqueuses.

## Résultats

L'arrêt de traitement était indiqué. L’évolution a été marquée par la disparition des lésions au bout de 3 jours.

## Discussion

La pharmacovigilance ne doit jamais se relâcher même pour un médicament largement prescrit comme le glucantime, utilisé en première intention dans la leishmaniose cutanée dans les pays du Maghreb. Le risque pour le patient est celui de l'excès de confiance du médecin.

## Conclusion

La vigilance s'impose quant au risque de toxidermie sévère avec cette molécule de plus en plus souvent prescrite.

# Research article: Ectropion bilatéral révélant une maladie de Hansen. Encore bien des mystères. Un dépistage sérieux doit-il être entrepris?

**Published:** 2023-03-02

**Authors:** Omar Boudghene Stambouli

**Affiliations:** Service de dermatologie, Faculté de médecine, Laboratoire Toxi Med 32, Université Abou Bekr Belkaid, Tlemcen, Algérie

**Keywords:** Macules achromiques, Ectropion bilatéral, Léprides, Griffe cubito-médiane, Lèpre

## Introduction

Depuis la nuit des temps, la lèpre accompagne l'humanité et son importance dépasse infiniment le nombre de malades. Motif de rejet brutal, cette pathologie est aussi un thème mobilisateur de toutes les bonnes volontés.

Considérée avant tout comme une pathologie d'importation, la maladie de Hansen demeure toutefois rare en Algérie..

## Observation

Il s'agit d'un patient S. A. né en 1922, demeurant à Hennaya (ex-Eugène Étienne) à 12 km au nord de la ville de Tlemcen (Ouest de l'Algérie), à 70 km de la frontière marocaine, et qui n'avait jamais quitté le pays. Son affection avait débuté 8 années auparavant. Lorsqu'il a été vu, étaient notées des achromies du front, des mains et des jambes. Des léprides du tronc et un ectropion bilatéral. Sur le plan neurologique étaient retrouvés un steppage, une hypo-anesthésie en gants et en chaussettes, une hypertrophie des troncs nerveux (radical, cubital, etc.), une griffe cubito-médiane bilatérale, une amputation de plusieurs phalanges, une paralysie faciale périphérique et un mal perforant plantaire. Le bilan suivant a été pratiqué:

La recherche du bacille de Hansen retrouvait 1 BAAR/100 champs au niveau du frottis de biopsie de l'oreille, mais s’était révélée infructueuse au niveau du mucus nasal.

L'examen anatomo-pathologique de la biopsie d'un lépride montrait un granulome tuberculoïde sans nécrose caséeuse.

L'IDR à la tuberculine était positive à 20 mm alors que l'IDR à la lépromine était négative. Finalement, a été retenu le diagnostic de lèpre interpolaire tuberculoïde BT.

L'enquête sur l'entourage ne retrouvait pas de cas similaires.

## Discussion

En Algérie, la lèpre n'est pas un problème de santé publique et par rapport aux pays limitrophes d'Afrique du Nord (Maroc, Tunisie) les cas recensés sont nettement moins nombreux.

Si dans le passé, l'origine étrangère de nombreux patients et l'existence du seul foyer kabyle ont rassuré, la description de cas sporadiques devrait peut-être amener à faire des enquêtes de dépistage. L'Ouest algérien, proche du Maroc, devrait en bénéficier, Oughanem y retrouvant 4 cas. De plus, cette rareté peut paraître surprenante si nous prenons en considération les 25 cas répertoriés par Rollier, cité par Kara-Zaitri en 1975, dans la seule province d'Oujda (Maroc), limitrophe de la région de Tlemcen. Historiquement en outre, la frontière n'a jamais été un barrage aux mouvements de population.

## Conclusion

La lèpre autochtone a toujours été rare en Algérie. Si l'existence d'un foyer ancien en Kabylie a toujours été reconnue, les cas sporadiques de l'Ouest algérien et particulièrement dans la région de Tlemcen, méritent l'intérêt des léprologues.

La situation géographique de nos pays impose une surveillance continue du fait des échanges multiples avec les pays à forte endémicité lépreuse.

# Research article: Aspect affichant, invalidant, handicapant d'un cas inhabituel de chromoblastomycose en zone à climat tempéré avec éléphantiasis bilatéral associé à un spondylolisthésis type 1 de L5/S1 sur lyse isthmique

**Published:** 2023-03-02

**Authors:** Omar Boudghene Stambouli

**Affiliations:** Service de dermatologie, Faculté de médecine, Laboratoire Toxi Med 32, Université Abou Bekr Belkaid, Tlemcen, Algérie

**Keywords:** Chromoblastomycose, Éléphantiasis bilatéral, Cellules fumagoïdes, *Fonsecaea pedrosoi*, Climat tempéré

## Introduction

La chromomycose (chromoblastomycose) est une dermatose chronique, localisée, due à des champignons dématiés appartenant principalement aux genres *Phialophora, Fonsecaea* et *Cladosporim.* Ces espèces sont représentées *in vivo* par une seule forme végétative pathognomonique, la cellule fumagine, dont la couleur spontanément brune, couleur de fumée, est à l'origine de la maladie. L’évolution rapide d'un cas de chromoblastomycose vers un éléphantiasis bilatéral, entraînant une dermatose affichante, handicapante et invalidante dans une zone à climat tempéré associée à un spondylolisthésis type 1 de L5/S1 sur lyse isthmique, nous incite à rapporter cette observation inhabituelle et exceptionnelle par l'aspect clinique et par l'association.

## Observation

Il s'agit de la patiente M. F. âgée de 47 ans, demeurant à Tlemcen (Ouest de l'Algérie), non mariée, femme au foyer, issue d'un milieu au niveau socio-économique et intellectuel bas; aux antécédents de spondylolisthésis type 1 de L5/S1 sur lyse isthmique et une attitude scoliotique avec bascule du bassin sur une coxarthrose droite sévère, rendant la marche impossible, obligeant la patiente à vivre confinée dans un fauteuil roulant.

Le début du trouble semble remonter à 2 ans, marqué par l'apparition de l’œdème des membres inférieurs et par la suite d'une lésion papulo-nodulaire unique sur la face dorsale du pied gauche puis une extension avec atteinte du pied droit également.

## Examen mycologique

L'examen microscopique direct des squames prélevées de la lésion cutanée et éclaircies par la potasse montre la présence des cellules fumagoïdes. La culture des squames cutanées et de prélèvements histologiques sur milieu de Sabouraud additionné d'antibiotiques met en évidence *Fonsecaea pedrosoi.*

## Discussion

La chromoblastomycose (chromomycose) est surtout observée dans les régions tropicales et subtropicales à cause du climat sec, mais elle peut cependant se voir en dehors de ces climats, surtout en ces périodes de réchauffement et de sécheresse au sud de la Méditerranée.

# Research article: Étude de la continuité des soins liés au VIH en période de pandémie Covid-19 entre mars 2020 et février 2021 au Sénégal

**Published:** 2023-03-02

**Authors:** Mamadou Makhtar Mbacké Leye, Ibrahima Mamby Keita, Ngone Mbaye

**Affiliations:** Service de médecine préventive et santé publique, Institut de santé et développement, Université Cheikh Anta Diop, Dakar, Sénégal

**Keywords:** Continuité des services, Prévention VIH, Covid-19, Sénégal

## Introduction

La pandémie à VIH/Sida est un des problèmes majeurs de santé publique. La pandémie de Covid-19 a impacté le système sanitaire au niveau mondial et national. Face à cette situation, il a été jugé nécessaire d’étudier la continuité de services liés au VIH au Sénégal en période de pandémie de Covid-19 entre mars 2020 et février 2021.

## Méthodologie

Une étude transversale à visée analytique a été menée entre mars 2020 et février 2021. La population d’étude était représentée par l'ensemble des PVVIH ayant adhéré à la file active entre mars 2020 et février 2021 et qui résident au Sénégal. Un sondage à plusieurs degrés a été effectué au niveau de chaque district. Des analyses univariée et multivariée ont été effectuées grâce aux logiciels Epi Info 3.3.2 et R 3.4.4.

## Résultats

L’âge moyen des enquêtés était de 43,32 ans ± 12,71 avec des extrêmes de 16 et 80 ans. Le sexe féminin prédominait soit 66,94% des cas, le sex-ratio H/F était de 0,49. Parmi les enquêtés, la majorité (63,35%) exerçaient une activité génératrice de revenu et ils s'inséraient pour la plupart dans le secteur informel qui représentait 68,69%. Depuis le début de la pandémie de Covid-19, 75,15% des enquêtés ont bénéficié d'une sensibilisation sur le nouveau coronavirus. Un peu moins de la moitié des enquêtés (47,98%) a reçu un accompagnement communautaire depuis le début de la pandémie. Les enquêtés qui avaient bénéficié de suivi à domicile représentaient 3,22%.

Les PVVIH âgés de 35 ans et plus (ORa 1,97 [1,16-3,33]) étaient plus à risque de subir une discontinuité des services liés à la prise en charge du VIH par rapport aux sujets âgés de moins de 35 ans. Les patients qui mettaient entre 30 minutes et 2 heures (ORa 0,31 [0,14-0,71]) ou moins de 2 heures (ORa 0,35 [0,14-0,94]) pour se rendre à la structure sanitaire étaient moins à risque de subir une discontinuité des services pour leur prise en charge. Les PVVIH ayant bénéficié d'un suivi à domicile (ORa 3,75 [1,23-11,49]) avaient 3,75 fois plus de risque de subir une discontinuité des services pour leur prise en charge que les personnes n'ayant pas bénéficié d'un suivi à domicile.

## Conclusion

Les résultats de ce travail suggèrent aux autorités sanitaires de réorganiser le système sanitaire et d'adopter une politique basée sur l'approche communautaire, et aux populations de surmonter la « peur de la Covid-19 ».
